# Refined Design and Liquid-Phase Assembly of GalNAc-siRNA Conjugates: Comparative Efficiency Validation in PCSK9 Targeting

**DOI:** 10.3390/molecules31030476

**Published:** 2026-01-29

**Authors:** Nikolai A. Dmitriev, Petr V. Chernov, Ivan S. Gongadze, Valeriia I. Kovchina, Vladimir N. Ivanov, Artem E. Gusev, Igor P. Shilovskiy, Ilya A. Kofiadi, Musa R. Khaitov

**Affiliations:** 1 National Research Center Institute of Immunology Federal Medical-Biological Agency of Russia, 115522 Moscow, Russia; 2 Department of Immunology, Faculty of Medicine and Biology, Pirogov Russian National Research Medical University, 117513 Moscow, Russia

**Keywords:** RNAi, siRNA, GalNAc, *PCSK9*, hypercholesterolemia, liquid-phase conjugation

## Abstract

The development and application of therapeutic oligonucleotides, such as siRNA, miRNA, ASOs and aptamers, is a rapidly growing field in biomedicine. These molecules are undergoing extensive preclinical and clinical testing, and the market for synthetic RNA drugs is expanding. However, several challenges remain, including targeted delivery and high costs associated with development, screening and production. One significant advance has been the creation of GalNAc-conjugates, which selectively target ASGPR and deliver oligonucleotides to hepatocytes. Although these conjugates have shown promising results, their widespread use is limited by the lack of effective synthesis methods. Thus, the development of new methods for the synthesis of ligand-oligonucleotide conjugates is an important task to which this study is devoted. In this study, we created a library of siRNA conjugates with the GalNAc L-96 ligand to suppress the expression of the *PCSK9* gene associated with elevated LDL and an increased risk of developing cardiovascular diseases. The selection of the most effective siRNA molecules was carried out using an algorithm previously developed by our research group, which considers thermodynamic stability, predicted specificity and effectiveness. To experimentally confirm the effectiveness of conjugates, an in vitro model based on the cultivation of hepatocyte cells was developed. Optimization of the conjugate synthesis process has significantly reduced the cost of manufacturing technology, which creates the potential for efficient scaling of synthesis for transfer and application in the pharmaceutical industry. The results of the study showed that the development of the siRNA sequence optimized in silico resulted in a significant increase in the inhibitory effect of the GalNAc-siRNA conjugate compared to a compound similar to a commercial drug.

## 1. Introduction

The possibility of short-term or reversible suppression of gene activity associated with disease pathogenesis is an undeniable advantage of therapeutic strategies based on synthetic oligonucleotides. However, the discovery of mechanisms involved in the negative regulation of gene expression, particularly RNA interference, drew attention not only to the benefits but also to the challenges of this approach. The main concerns include off-target effects, stability, immunogenicity and shortage of methods for selective delivery of oligonucleotides to target tissues and organs [1,2,3,4]. These factors must be considered in the development of modern therapeutic oligonucleotide treatments. Understanding the molecular mechanisms of interaction of therapeutic oligonucleotides with their targets opens up new possibilities for their practical use.

In silico modeling of therapeutic oligonucleotides using computational methods allows us to predict the effectiveness of molecules, evaluate their stability and minimize off-target effects [5]. In particular, when choosing effective small interfering RNAs (siRNAs), the key step is selecting the correct target sequence. This directly depends on the primary and secondary structure of the messenger RNA (mRNA) fragment to which the siRNA binds. It is also important to characterize the non-covalent bonds that provide the best suppressive effect. To solve these problems, an integrated approach is used, combining empiric rules, effective information search on proven bioinformatics resources and machine learning methods [6,7,8]. This allows us to shift the focus from synthesizing and testing potential sequences to computer modeling, and thus, we can predict their effectiveness with increased accuracy.

The main challenge for the therapeutic oligonucleotide industry is to establish a comprehensive pipeline for drug development, including the design and synthesis of compound libraries. Moreover, since conjugates with oligonucleotides are widely used, it is essential to integrate the synthesis and screening stages of a large number of different conjugates in order to efficiently find the optimal ligand for delivering to target tissues.

The use of GalNAc conjugates capable of tissue-specific therapeutic oligonucleotide delivery to hepatocytes via binding to the ASGPR receptor, which is almost exclusively expressed on their surface, is a significant approach to RNA therapy for liver diseases. This has drastically increased the effectiveness and safety of these treatments [9,10]. This uncovers vast prospects for the development of new therapeutic agents due to the cornerstone position of the liver in numerous metabolic pathways. However, there is a number of challenges and hurdles associated with the production of GalNAc conjugates. These include the multi-step synthesis process and the difficulty of purification and validation, which increase the cost and consequently the availability of these drugs [11]. This situation encourages exploration and optimization of new methods for synthesizing these compounds. Since this type of ligand-oligonucleotide conjugate is the most widely studied and there are drugs in clinical practice that belong to this class [9], the use of this ligand is justified for a meaningful comparison of synthesis approaches.

**Figure 1:**
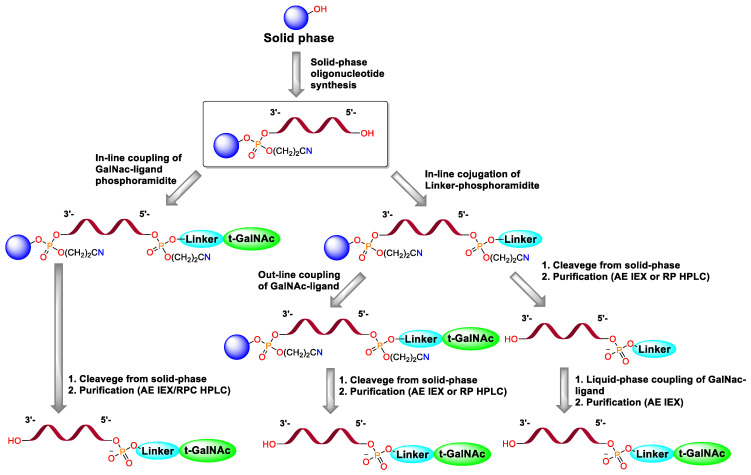
Strategies for the synthesis of 5′-conjugates in the solid and liquid phases.

Based on the means of ligand introduction, three main approaches to conjugate synthesis can be distinguished: in-line conjugation, out-line solid-phase conjugation and post-synthetic liquid-phase conjugation (Figure 1) [12,13]. Despite the fact that solid-phase conjugation approaches are simpler, the synthesis efficiency is lower and the consumption of necessary ligands is significantly higher than in the case of liquid-phase ligand addition [11,14]. If we are talking about in-line conjugation via a phosphodiester bond, the use of GalNAc-phosphoramidite is necessary, which imposes restrictions on the type of ligands used and requires linear synthesis of phosphoramidite, which can be a difficult task. In cases of out-line conjugation and post-synthetic liquid-phase conjugation, it is possible to divide triantennary GalNAc into a linker and a ligand itself, the synthesis of which can be realized in parallel, which can be used to create libraries containing various ligands. In case of conjugate syntheses in the solid phase, there is a restriction on the conditions for the stage of cleavage from the solid phase, since degradation of the ligand can be observed [14]. In addition, the separation of synthesis into solid and liquid phases leads to significant simplification of the purification, since the final reaction mixture will not contain minor N-1 impurities that appear at the stage of solid-phase synthesis and which are difficult to remove by chromatography.

Combining new synthesis technologies with existing design methods opens up new opportunities for the search for therapeutic candidates, and in this case, can be used to create libraries of conjugates with other ligands and therapeutic molecules, including small molecules and peptides. In this study, we developed a new synthesis strategy and tested it on the GalNAc–L96 model ligand, demonstrating increased efficiency and reduced cost of synthesis, as well as effective suppression of the *PCSK9* model gene.

## 2. Results

### 2.1. Design of siRNA Sequences

We selected the *PCSK9* gene as a target. An mRNA transcript of this gene (NM_174936.4M) was used to select optimal small interfering RNAs aimed at suppressing *PCSK9* gene expression. A total of 815 candidate siRNA duplexes complementary to the *PCSK9* mRNA sequence were generated.

**Figure 2:**
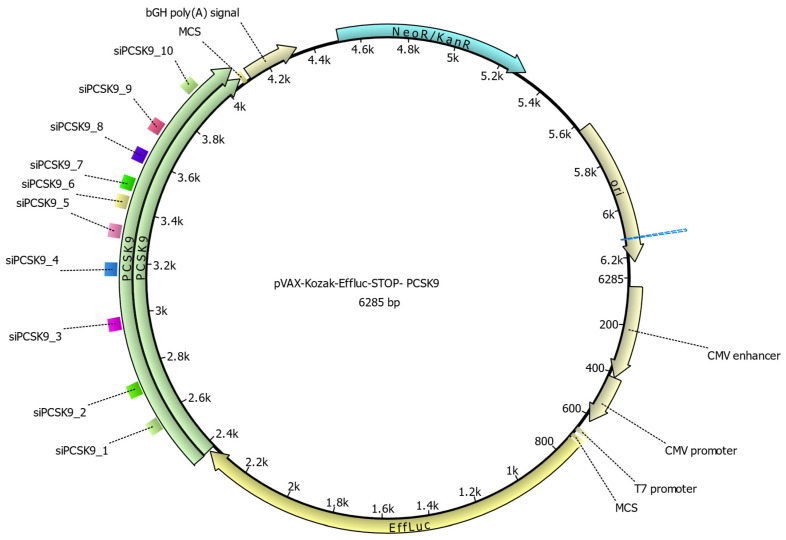
A map of the pVax-Kozak-Effluc-STOP-*PCSK9* reporter plasmid with marked interference sites of choice molecules.

Based on the results of the initial processing of the obtained sequences, 126 siRNA duplexes were selected and processed using a more rigorous algorithm to identify the most promising sequences for in vitro testing. In the format of this trial, 10 of the most promising duplex sequences were selected, one of which coincides with the drug Inclisiran [15]; therefore, this sequence was used as a positive control in subsequent in vitro tests (Figure 2).

The criteria and algorithms are described in more detail in the Materials and Methods Section and in the Supplementary Materials.

### 2.2. Synthesis and In Vitro Testing of Unmodified siRNA Sequences

A total of 20 unmodified RNA sequences were synthesized, which were used to form 10 siRNA duplexes. The simplices of the target sequences were characterized using MALDI-TOF MS.

The RNA sequences and selection criteria are described in more detail in the Materials and Methods Sections and in the Supplementary Material.

**Figure 3:**
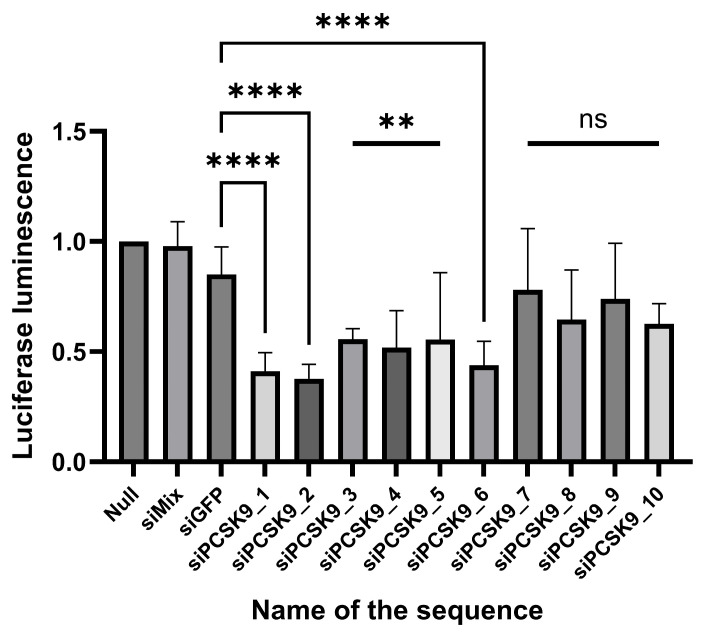
The activity of unmodified siRNA against the human *PCSK9* gene in vitro on HEK293T cell culture in a non-infectious mRNA expression model of the corresponding gene. Proportion of luciferase luminescence, relative to control (null): siPCSK9_1 = 0.3964, siPCSK9_2 = 0.3524, siPCSK9_3 = 0.5485, siPCSK9_4 = 0.4790, siPCSK9_5 = 0.5900, siPCSK9_6 = 0.4237, siPCSK9_7 = 0.8080, siPCSK9_8 = 0.6768, siPCSK9_9 = 0.7186, siPCSK9_10 = 0.6071, within the margin of error of SD. The statistical analysis was performed using the Kruskal–Wallis criterion. Statistically significantly different from “null”—statistically significantly different from “siMIX” at *p* < 0.001. N = 7. (ns—statistically non-significant difference, **—*p* < 0.01, ****—*p* < 0.0001).

An in vitro experiment on HEK293T cell culture transfected with expression plasmids containing a full-length insertion of the target gene and a reporter gene (see Materials and Methods) revealed the ability of all developed siRNA duplexes (siPCSK9) to suppress *PCSK9* gene expression (Figure 3). For this purpose, the change in the expression level of the target *PCSK9* gene in cells after their treatment with siRNA molecules was calculated relative to cells that were not subjected to such treatment—“null”. Nonspecific siRNA molecules were used as negative controls: a mixture of siMix (UTR regions of the hepatitis C virus genome) and siGFP (green fluorescent protein gene). Data analysis showed that the siRNA variants siPCSK9_2 and siPCSK9_6 provided the greatest silencing of the target gene expression (Figure 3). Treatment of cells with these siRNA variants resulted in a decrease in *PCSK9* gene expression of more than 2-fold compared to non-specific siRNAs (siMIX). The maximum decrease in the expression of the target gene was provided by the siPCSK9_2 variant.

The effectiveness of suppression of *PCSK9* gene expression by siPCSK9_2 and siPCSK9_6 duplexes was comparable to the effectiveness of the positive control, a sequence analog of the siRNA sequence of Inclisiran [15], which is designated as siPCSK9_1.

### 2.3. Synthesis and In Vitro Testing of Modified siRNA Sequences

At the next stage, 12 antisense chain modification patterns as well as 4 sense chain modification patterns were designed; 12 modified duplexes were synthesized for each selected highly efficient unmodified siRNA, and the siPCSK9_2_N and siPCSK9_6_N series were formed. Sequences and modifications are presented in the Supplementary Materials.

**Figure 4:**
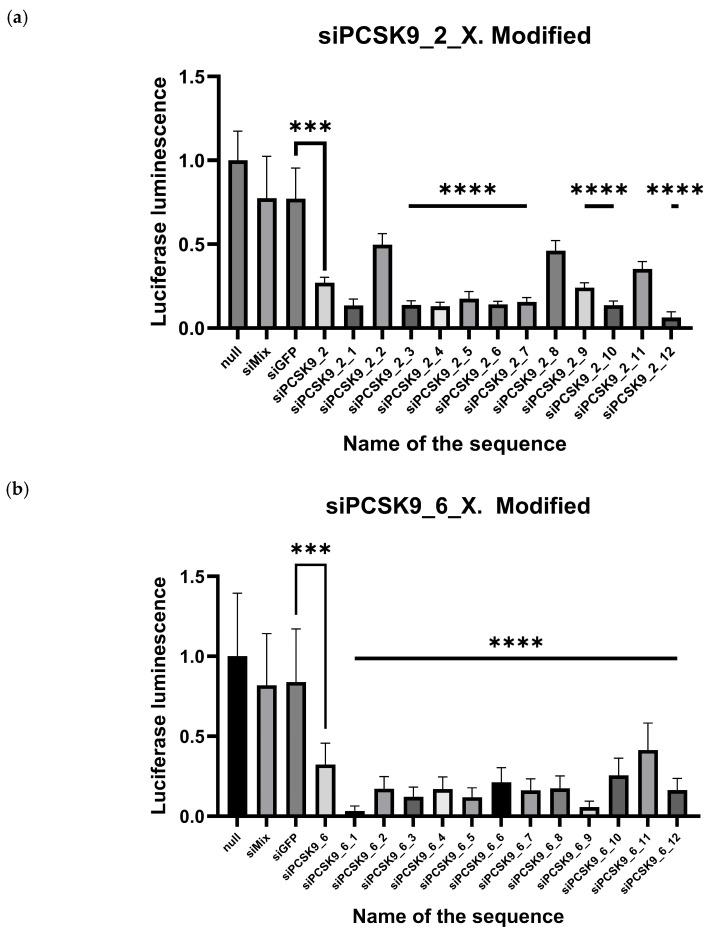
Results of in vitro testing of the activity of modified siRNA sequences on HEK293T cell culture: (**a**) The activity of siRNA of the siPCSK9_2_X series against the human *PCSK9* gene in vitro in a non-infectious mRNA expression model of the corresponding gene. Proportion of luciferase luminescence, relative to control (null): Proportion of luciferase luminescence, relative to control: siPCSK9_2 (unmodified) = 0.2706 siPCSK9_2_1 = 0.1358, siPCSK9_2_2 = 0.4982, siPCSK9_2_3 = 0.1383, siPCSK9_2_4 = 0.1303, siPCSK9_2_5 = 0.1761, siPCSK9_2_6 = 0.1424, siPCSK9_2_7 = 0.1574, siPCSK9_2_8 = 0.4620, siPCSK9_2_9 = 0.2415, siPCSK9_2_10 = 0.1368, siPCSK9_2_11 = 0.3534, siPCSK9_2_12 = 0.0628 within the margin of error of SD. The statistical analysis was performed using the Kruskal–Wallis criterion. Statistically significantly different from “null”—statistically significantly different from “siMIX” at *p* < 0.0001. N = 7, (**b**) The activity of siRNA of the siPCSK9_6_X series against the human *PCSK9* gene in vitro in a non-infectious mRNA expression model of the corresponding gene. Proportion of luciferase luminescence, relative to control (null): Proportion of luciferase luminescence, relative to control: siPCSK9_6 (unmodified) = 0.3249, siPCSK9_6_1 = 0.0334, siPCSK9_6_2 = 0.1719, siPCSK9_6_3 = 0.1224, siPCSK9_6_4 = 0.1693, siPCSK9_6_5 = 0.1192, siPCSK9_6_6 = 0.2132, siPCSK9_6_7 = 0.1617, siPCSK9_6_8 = 0.1744, siPCSK9_6_9 = 0.0574, siPCSK9_6_10 = 0.2562, siPCSK9_6_11 = 0.4146, siPCSK9_6_12 = 0.1628, within the margin of error of SD The statistical analysis was performed using the Kruskal–Wallis criterion. Statistically significantly different from “null”—statistically significantly different from “siMIX” at *p* < 0.0001. N = 7. (***—*p* < 0.001, ****—*p* <0.0001).

Afterwards, the synthesized molecules were subjected to an in vitro study under the conditions of the same model. The study showed that all modified siRNA duplexes of the siPCSK9_2_X and siPCSK9_6_X series significantly reduced *PCSK9* gene expression compared with negative controls (siMix and siGFP). The duplexes were separated into two groups: “odd” (X in the name of the sequence is an odd number) and “even” (X in the name of the sequence is an even number). The introduction of modifications made it possible to increase the biological activity of siRNA duplexes relative to their unmodified analogs (siPCSK9_2 and siPCSK9_6) (Figure 4).

According to the results of the in vitro study, it appears that the majority of odd variants of modifications greatly increase the efficiency of gene silencing, whereas the majority of even variants have lost their efficacy. In some cases, chemical modification decreased siRNA activity to such an extent that it became worse than the activity of unmodified siRNA.

Notably, siRNA variants whose antisense strands possess a more significant difference at 10–11 nucleotides as well as 5′mXfX moieties and 3′mXmX or 3′fYmY (siPCSK9_2_1, siPCSK9_2_3, 2_12, siPCSK9_6_1, siPCSK9_6_5, siPCSK9_6_9) show a higher RNAi efficacy (Table 1).

Candidate molecules for the formation of GalNAc conjugates were selected based on the obtained data on biological activity. The selected sequences are presented in the Supplementary Materials.

### 2.4. Synthesis of GalNAc-siRNA Conjugates

Based on the results of in vitro tests, sequences were selected for the formation of conjugates with GalNAc. Two in-line approaches were implemented at both the 3′-end and the 5′-end, solid-phase synthesis and post-synthetic liquid-phase conjugation, in order to compare the effectiveness of these approaches.

**Figure 5:**
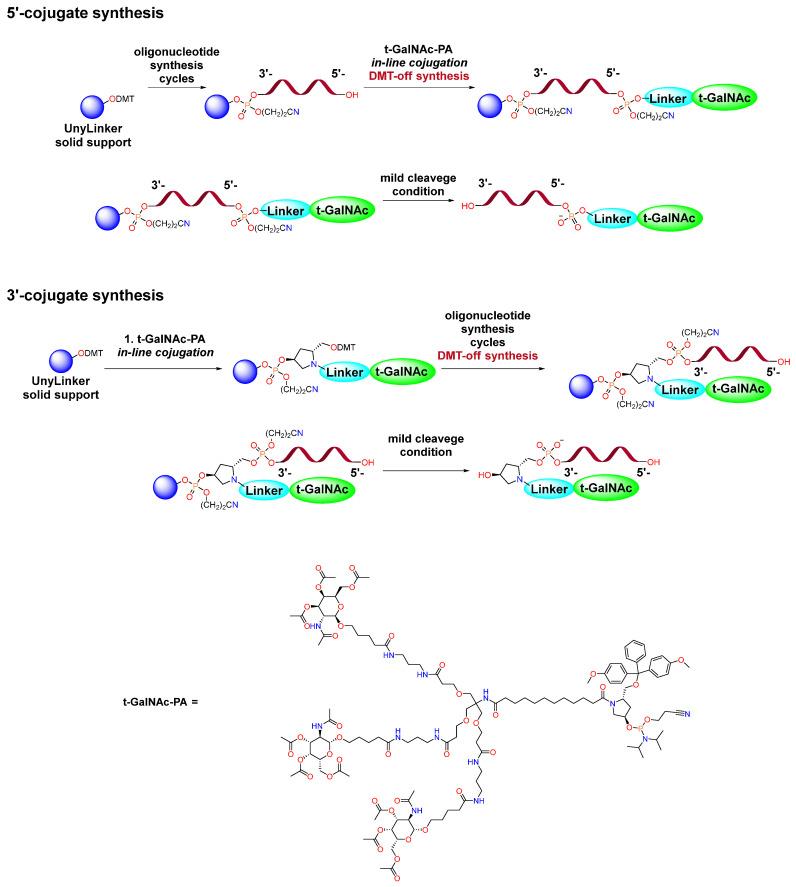
Scheme of in-line solid-phase synthesis of GalNAc-oligonucleotide conjugate.

Solid-phase oligonucleotide synthesis was performed using standard protocols [14,16]. In the case of the in-line approach, commercially available GalNAc-L96 Phosphoramidite was used. The synthetic scheme is shown in Figure 5.

Standard methods were used to remove conjugates from the solid phase (Table 2). The removal of acetyl protectors from the GalNAc fragment was carried out at the stage of conjugate removal from the solid phase. The structure of the obtained conjugates was confirmed using mass spectrometry methods.

In case of post-synthetic liquid-phase modification, the synthesis of oligonucleotide sequences was carried out using standard solid-phase synthesis protocols [14,16].

**Figure 6:**
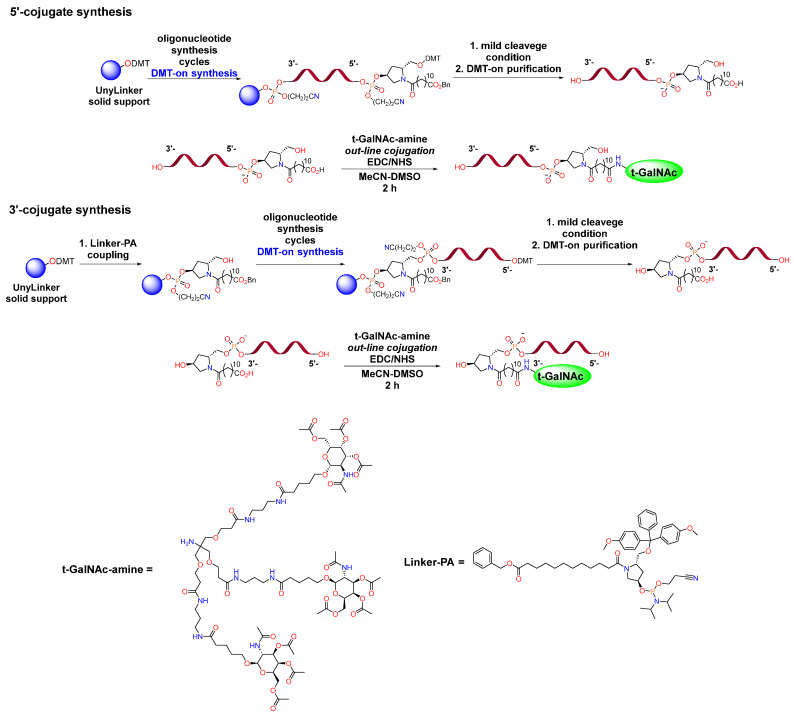
Scheme of synthesis of GalNAc-oligonucleotide conjugate through post-synthetic liquid-phase modification.

The introduction of a modified fragment containing an ester group at the 3′- or 5′-ends was also carried out using standard solid-phase oligonucleotide synthesis protocols. This was followed by the cleavage of modified oligonucleotide sequences from the solid phase, and conditions were found in which, in addition to cleavage, hydrolysis of the ester group occurs. The synthesis was carried out DMT-on, which effectively removed N-1 impurities. The amidation reaction with a purified modified oligonucleotide containing a carboxyl group was carried out to obtain target conjugates in the liquid phase (Figure 6).

For the stage of cleavage of the modified oligonucleotide, optimal reagents and conditions were selected. The key stages of the reaction were as follows:

- Cleavage from the solid phase;
- Removal of protective groups from nucleotides;
- Hydrolysis of the ester group.

The results of optimization of this stage in order to meet all requirements are presented in Table 3.

In the case of liquid-phase post-synthetic conjugation due to the amidation reaction between modified oligonucleotides containing a carboxyl group and an amine containing a GalNAc fragment, optimal reaction conditions were also selected. The optimization results of this stage are presented in Table 4.

After the key amidation step, the target conjugates were purified using AE IEX. The structure of the obtained compounds was confirmed using MALDI-TOF mass spectrometry. AE IEX chromatography allows the removal of acetyl protective groups due to the basicity and composition of the subphase phase. The structures of the conjugates obtained were confirmed using mass spectrometry methods.

This approach made it possible to obtain target conjugates with a low level of degradation and a high degree of purification, which were later used for in vitro testing.

### 2.5. Results of In Vitro Testing of Target GalNAc-Oligonucleotide Conjugates

The biological activity of the 5′-GalNAc conjugate siPCSK9_2_12-C was studied in a series of in vitro experiments on a culture of human hepatocellular carcinoma cells of the Huh7 line. The 3′-GalNAc conjugate siPCSK9_1-C, which is similar in modifications to the commercial drug, was used as a reference. The cells were transfected with the studied complexes at a concentration of 1 µM. Transfection was performed without the use of transfection agents.

Following 24, 48 and 72 h of transfection, total RNA was collected and used in the reverse transcription reaction to obtain cDNA. Quantitative PCR (qRT-PCR) was used to analyze the effectiveness of *PCSK9* gene suppression. β-actin (*ACTB*) was selected as the reference gene (housekeeping gene). The relative expression level (RQ) was calculated using the ΔΔCq method.

Treatment of cells with siPCSK9_1-C and siPCSK9_2_12-C complexes had no statistically significant effect on the expression of the *ACTB* reference gene, whose Ct values remained stable and averaged 25.00 ± 0.04 in all experimental groups. This indicates the specificity of the drugs and the stability of the experimental conditions.

On the first day of the experiment, the PCR product of the *PCSK9* gene in cells treated with siPCSK9_1-C was determined, on average, at 25.38 cycles and with siPCSK9_2_12-C at 25.53. When calculating ΔΔCq/K, the expression of the target gene after treatment with siPCSK9_1-C was 0.719 and with siPCSK9_2_12-C it was 0.674. On the second day of the experiment, the PCR product of the *PCSK9* gene in cells treated with siPCSK9_1-C was determined, on average, at 26.77 cycles, and with siPCSK9_2_12-C, at 27.26. When calculating ΔΔCq/K, the expression of the target gene after treatment with siPCSK9_1-C was 0.325 and with siPCSK9_2_12-C it was 0.245. On the third day of the experiment, the PCR product of the *PCSK9* gene in cells treated with siPCSK9_1-C was determined, on average, at cycle 27.02 and with siP-CSK9_2_12-C at 28.43. When calculating ΔΔCq/K, the expression of the target gene after siPCSK9_1-C was 0.246 and with siPCSK9_2_12-C it was 0.111.

**Figure 7:**
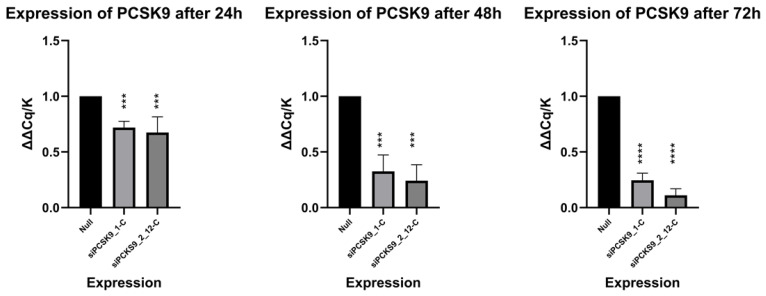
qRT-PCR results. The studied samples are located along the abscissa axis. On the ordinate axis is the value ΔΔCq/K. Statistical processing was performed using the two-way ANOVA and nonparametric Kruskal–Wallis criterion for N = 3. For the first and second graphs (Expression of *PCSK9* after 24 and 48 h), the differences are statistically significant at *p* < 0.0001. For the third (Expression of *PCSK9* after 72 h), the differences are statistically significant with a *p* value < 0.0001. (***—*p* < 0.001, ****—*p* < 0.0001).

According to the results, the developed conjugate siPCSK9_2_12-C demonstrated comparable effectiveness at all time points (24, 48, 72 h) compared to siPCSK9_1-C. The maximum effect of suppressing the expression of the target *PCSK9* gene for both compounds was observed after 72 h. The siPCSK9_1-C compound reduced the *PCSK9* mRNA level to 24.6% compared with the control (cells without treatment), while the siPCSK9_2_12-C complex under study decreased to 11.1% (Figure 7).

## 3. Discussion

Based on the results obtained in this study, we can conclude that our research group has managed to implement a successful in silico design that has allowed us to obtain potential therapeutic candidates. However, due to the fact that the therapeutic use of oligonucleotides requires the presence of a fragment responsible for delivery, we needed to synthesize conjugates for further in vivo studies. Thus, an experimental validation of the algorithm for sequence design was carried out, and a new effective method for synthesizing GalNAc conjugates was proposed. Since both of these issues are relevant to the scientific community, we have combined them into one work.

### 3.1. Additional Criteria for Selecting Sequences and Modifications

Our previous research [3] allowed us both to supplement the algorithm with additional selection criteria and to better understand the principles of siRNA modification (Table 5).

**Figure 8:**
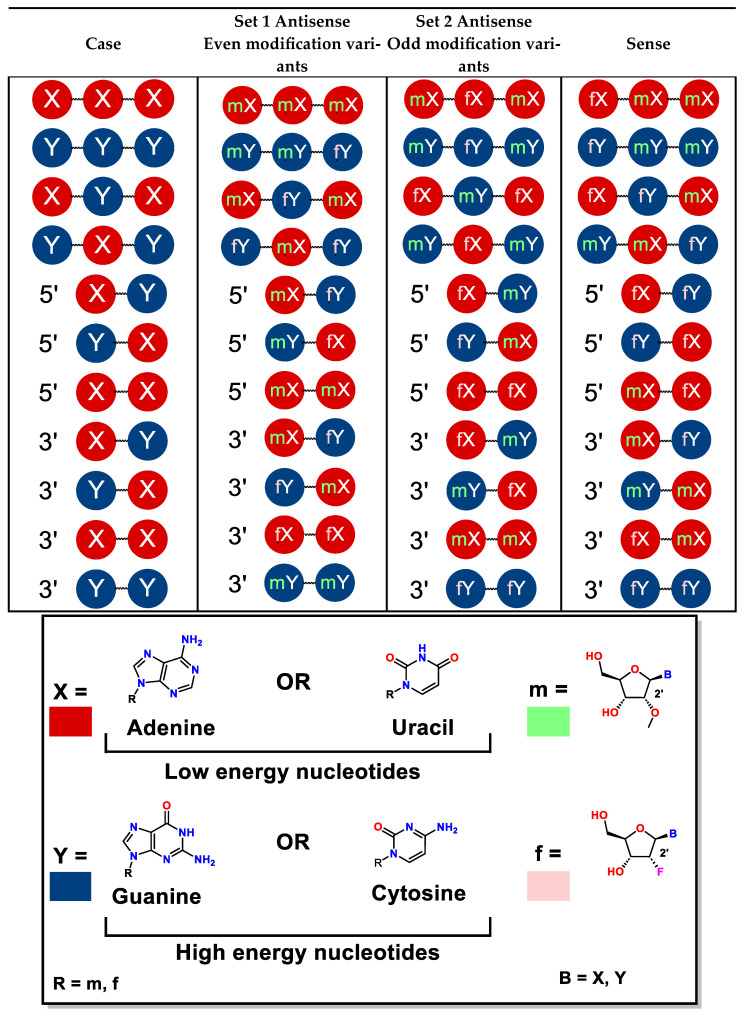
Variants of modification patterns and chemical structures of 2′-F-substituted and 2′-OMe-substituted nucleotides.

Since siRNAs show functional segmentation for more optimal binding to mRNA in the complex with RISC, the specificity and activity of siRNAs are largely determined by the distribution of functions between these segments, which is important to consider in the design [17]. The seed region (2–8 positions of nucleotides) provides effective specific recognition through complementarity and serves as the main binding factor to the mRNA target. In recent years, functional subsegments have been identified that are sensitive to chemical modifications in the seed region [18]. The central region (9–12 nucleotide positions) and the 3′-terminal regions ensure the stabilization of the complex and promote the cleavage of the target [19]. Thus, understanding the structural and functional organization of siRNA is critically important in creating more selective and safe therapeutic candidates. Based on this, in the context of developing modification patterns, we divided monomers into two groups A/U and G/C. Since the energy of interaction of complementary pairs directly depends on the number of hydrogen bonds formed between them, it is possible to symbolize the nucleotides A/U as “low-energy” (X) and G/C as “high-energy” (Y) (Figure 8). In this study, we have carefully studied the introduction of 2′-F-substituted and 2′-OMe-substituted nucleotides, as these modifications are the most common for siRNA.

Despite some siRNAs decreasing their efficiency after modification, it is obvious that the majority of odd modification variants are more favorable to the overall activity of siRNAs, whereas even approaches tend to decrease the efficiency of the interference. It turned out that in the case of alternating high- and low-energy monomers, one should assign them different modifications, so the fragment of sequence XYX increased the interference efficiency if it was composed of mXfYmX monomers. In the case of the formation of islands of equal-energy monomers in the sequence, the modifications mYfYfY or mYmYfY and fXfXmX or fXmXmX were more effective. In positions 10 and 11, the presence of both high-energy and low-energy monomers, modified in accordance with the adjacent fragments of the antisense chain, was critically important. When a fragment with a length of more than four nucleotides is formed in the sequence, its division into smaller segments, for example, mXfXfXmX or fYmYmYfY, turned out to be significant. The segments of communication between high- and low-energy islands were of particular importance. In this case, the mYfYmXfX patterns showed the greatest effectiveness.

These results are in accordance with the latest fundamental research [20] and prompt a curious hypothesis considering the intricacies of RNA-dependent machinery. Apparently, the most impactful features of the patterns of modifications are the high energy difference between nucleotides 10 and 11 of the antisense strand, the reduction of overall energy density on the 5′-end and the increase of energy density on the 3′-end. This not only encourages AGO2 for the selection of an antisense strand [21] but also facilitates its proper positioning against mRNA, which enhances RNAi potency.

Using these methods, we managed to achieve a more than 5-fold increase in interference efficiency with a relatively small number of attempts, combined with the formation of significant resistance to exonucleases and endonucleases.

### 3.2. Experimental Validation of siRNA Sequences Design

The algorithm described in this work made it possible to carry out a comprehensive bioinformatic analysis of the *PCSK9* gene, which is associated with the pathogenesis of atherosclerosis and an increased risk of developing cardiovascular diseases. The result of the analysis is an effective shrinking of the candidate library from 815 to 14 sequences, which, after two rounds of selection, received the highest scores (>12 out of 15), of which 10 were selected for in vitro validation. The experimental results confirmed the high accuracy of the approach used since four of the ten samples studied reduced gene expression by more than 50% (siPCSK9_1, siPCSK9_2, siPCSK9_4, siPCSK9_6), three samples reduced expression by more than 40% (siPCSK9_3, siP-CSK9_5 siPCSK9_10) and only the remaining three samples were found to be insufficiently effective and reduced expression by less than 30% (siPCSK9_7, siPCSK9_8, siPCSK9_9). These results illustrate the effectiveness of integrating existing algorithms into the developed evaluation mechanism, which imposes additional requirements on the physico-chemical properties of sequences, thermodynamic parameters and risk assessment of inappropriate interactions. Automation of data processing and visualization of binding sites significantly reduced labor costs and accelerated the transition from the in silico stage to the in vitro stage.

Despite the good efficiency results, the proposed approach has a number of limitations. As with most computational methods, this approach does not always successfully reflect biological activity, and the possibility of limited access to certain databases may reduce the effectiveness of off-target effects assessment [22]. In addition, a number of criteria described in this work require additional adjustments and empirical validation, as well as additions and adaptations to specific types of cells, target genes and siRNA sequences.

To increase the versatility and predictive accuracy of this method, as part of further research, it is possible to expand the number of parameters by considering epigenetic and transcriptomic data from specific cell lines, integrating data on the availability of mRNA sites and implementing machine learning models based on the results of in vitro and in vivo studies. The development of this method in this direction will not only improve accuracy and efficiency but will also reduce the number of false positive prediction results, which is critically important to accelerate the process of developing and creating new therapeutic candidates. An important area of development is the integration of a module for predicting the optimal chemical modifications of siRNAs, aimed at increasing activity, stability and reducing immunogenicity [20,23]. In the future, the integration of this section will not only increase the efficiency of candidate prediction and the accuracy of selection but will also allow for more precise modeling of the pharmacokinetic properties of the developed molecules in advance.

Current scientific knowledge of the intricacies of the RNAi apparatus does not allow for the concrete delineation of the causative factors of siRNA efficacy. Therefore, it does not seem reasonable to compare the impact of chemical modifications on the efficacy of different siRNA sequences. Nevertheless, it stands to reason that within the framework of the same model, the extent of RNA interference of the different siRNAs may be compared. In this article we report on the entire development cycle of the siRNA preparation and compare our design’s efficacy to the effect of an already approved and tested sequence of the drug Inclisiran. Notably, the sequence of Inclisiran has more thiolated phosphodiester bonds, which greatly increase the stability of the duplex and play an important role during intracellular GalNAc separation [24]. Yet, we do not observe a significant impact of PS-linkages on the effectiveness of a certain siRNA sequence. Therefore, it is challenging to attribute the change in efficacy to the number of PS-linkages introduced and we are inclined to believe that the major impact on the efficacy is provided by the sequence itself [24].

Admittedly, the chosen siRNA sequences have significant off-targets presented in Materials and Methods. Nevertheless, these genes are neither exclusively expressed by the liver nor carry out significant liver-related functionality. As for the suppression of the activity of these genes in other tissues, it remains a significant risk and may be a difficult hurdle to overcome. However, GalNAc carrier molecules possess distinct hepatotropic properties due to being ligands for ASGPR, which is almost exclusively expressed by the hepatocytes. Therefore, it is reasonable to claim that the implementation of GalNAc effectively removes the concern for possible extrahepatic off-target effects. Nevertheless, we will be testing this claim in our future in vivo biodistribution experiments.

Thus, the presented approach demonstrates good reproducibility, scaling potential and is a contribution to the improvement of rational design tools for drugs based on RNA interference.

### 3.3. Comparison of Approaches to Synthesis of GalNAc-siRNA Conjugates

In this study, two main approaches to the synthesis of GalNAc-siRNA conjugates were implemented: in-line solid-phase and post-synthetic liquid-phase conjugation, both at the 5′- and 3′-ends. Based on the results obtained in this study, a number of advantages and disadvantages for each approach can be identified.

**Figure 9:**
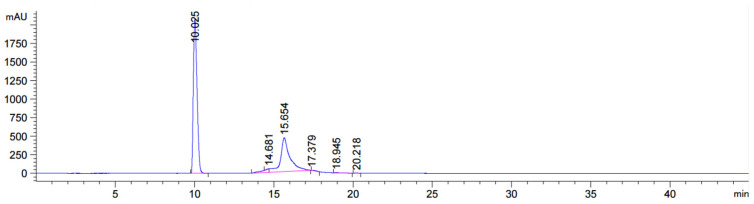
RP HPLC for crude 5′-conjugate synthesized by solid-phase synthesis. The main product is the initial sequence without the GalNAc (Ret.Time: 10.025 min, Area: 62.8101%) fragment; the target product is a minor.

To a large extent, the standard approach with the introduction of a GalNAc fragment during the solid-phase synthesis of an in-line oligonucleotide may be characterized by greater simplicity of synthesis, since this does not require the implementation of an additional stage and, as a result, additional purification steps, which allows to automate this process; however, this method is rather more applicable for the 3′ conjugate, due to the commercial availability of the solid phase, which already contains the GalNAc fragment; therefore, synthesis takes place using standard protocols [14,16]. Unfortunately, the commercially available solid phase containing a preloaded carrier molecule does not have a high capacity, which hinders solid-phase synthesis at an industrial scale. In the case of using sterically loaded phosphoramidite containing a triantennary GalNAc fragment for conjugation at the 5′-end, a significant decrease in efficiency was observed along with a significant increase in reaction time, which was not resolved by an increased concentration of the amidite. It is also important to note that the standard conditions for conjugate cleavage from the solid phase can also significantly contribute to conjugate degradation, thereby reducing the efficiency of synthesis (Figure 9). Even though DMT-on synthesis enables simplified purification from the main N-1 impurities, attempting it leads to degradation of the target conjugate during detritylation. Thus, regardless of the position of the GalNAc fragment on the chain, it was decided to use AE IEX to purify from N-1 impurities. All in all, it can be noted that despite the potential automation and fewer stages, the difficulties associated with these stages significantly reduce the potential for scaling, which makes this approach primarily a laboratory synthesis method.

On the contrary, combining in-line solid-phase synthesis of an oligonucleotide modified by a fragment containing a functional group necessary for conjugate formation, as well as post-synthetic liquid-phase conjugation, requires a larger number of stages and, consequently, purification steps. However, these processes are much simpler due to the large difference in the physical-chemical properties of the products and impurities.

Also, there were no differences between the addition methods in the use of phosphoramidites containing an ester group during the solid-phase synthesis, which indicates that this phosphoramidite is not inferior in reactivity to the standard phosphoramidites of modified nucleotides. Moreover, synthesis using the linker can be carried out DMT-on, both in the case of location at the 3′- and 5′-ends. Unfortunately, the standard methods of nucleotide cleavage in the solid phase were not applicable, since at this stage it was important to consider all the criteria for the reaction. The key problem is the potential for the formation of an amide due to the reactivity of the ester group, as well as incomplete hydrolysis, since the benzyl ether is quite stable. We optimized the conditions that allow for complete separation from the solid phase with the complete removal of protective groups, including the hydrolysis of benzyl ether, from which we successfully obtained a fragment of the carboxyl group, but no chain degradation was observed. However, this stage also requires additional optimization, since there was a slight formation of an amide bond hydrolysis byproduct in the linker, but this did not have a strong effect on the synthesis efficiency. Within the framework of this study, our main goal was to show the fundamental possibility of such an approach on a model substrate, and this problem can be successfully solved using other commercially available phosphoramidites containing an ester group. The obvious fact is that the presence of a dimethoxytrityl protective group greatly simplifies the purification of the target sequence from N-1 impurities. In this way, we obtained the target sequence of an oligonucleotide containing a carboxyl group, which made it possible to use it in the key stage of amidation in the liquid phase. The main problem of this stage was the poor solubility of the modified oligonucleotide sequence in standard organic solvents such as DCM and acetonitrile. Therefore, an approach was implemented using standard polar aprotic solvents for the amidation reaction, such as DMF and DMSO mixed with acetonitrile. In this case, according to the results obtained in this study, at this stage the most important part is the selection of a suitable capping reagent since there are no protective groups on the oligonucleotide in free form and it is important to maintain a balance between the activation of the carboxyl group and the absence of side effects with free functional groups. Due to this problem, standard capping reagents such as HBTU, BOP, CDI and EDC largely failed to produce the target product. The best result was demonstrated when using the EDC/NHS combination, since EDC itself is extremely reactive, a significant excess of NHS (50 eq.) relative to the oligonucleotide was used to form the target NHS ester and reduce the likelihood of the formation of by-products. The NHS ether obtained in situ is known to be resistant to water and hydroxyl groups. On the other hand, due to the steric loading of the carboxyl group, there was sometimes a lack of reaction; this problem was also solved due to a significant excess of reagents relative to the oligonucleotide.

No degradation of the target molecule was observed during the conjugate formation and purification. A significant advantage of solid-phase synthesis is the ability to control the reaction with the addition of an amine, if necessary, for complete conversion. After the reaction is complete, it is possible to remove acetyl protective groups from the GalNAc fragment using a solution of potassium carbonate [25] under milder conditions, relative to its use in solid phase separation. However, when using AE IEX for purification, the removal of these protective groups is carried out due to the basicity of the eluent. In this case, it is not a significant problem to purify the target compound using AE IEX, since the product and impurities have a large difference in physico-chemical properties and, as a result, retention times in the stationary phase.

**Figure 10:**
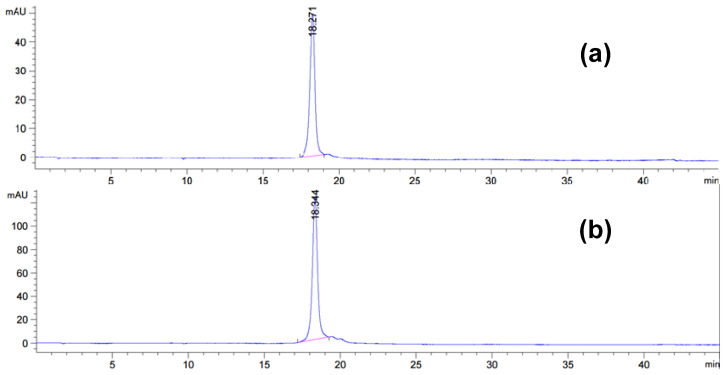
Analytical RP HPLC chromatograms after preparative AE IEX for 5′-end conjugate synthesized by solid-phase synthesis (**a**) and 5′-end conjugate synthesized by post-synthetic liquid-phase synthesis (**b**).

Thus, based on the totality of the results obtained, it can be concluded that the use of DMT-on protocols for synthesis and purification is not the most significant advantage, since we were able to achieve the production of high-purity conjugates in each of the cases using AE IEX (Figure 10), but, at the same time, DMT-on protocols can be applied in case of other ligands.

The key difference is observed in the total efficiency of the entire synthesis, which is presented in Table 6. Despite the greater number of stages, the use of post-synthetic liquid-phase conjugation shows better results relative to standard solid-phase synthesis. Moreover, the possibility of varying different amines, in this case, may be more applicable to create libraries containing different ligands.

The efficacy (yield) of each stage in the in-line and out-line conjugations was determined using data from HPLC, MALDI-TOF MS and spectrophotometry. All syntheses were performed at a scale of 1–5 µmol. Yields from HPLC were calculated as the percentage ratio of the target product peak area to the total area of all oligonucleotide-containing peaks in the crude mixture. MALDI-TOF MS was used to confirm fraction purity after preparative chromatography, based on the relative intensities of target and side-product peaks. For purification stages, yields were assessed spectrophotometrically by UV absorbance at 260 nm: concentrations before and after the stage were measured in triplicate using sequence-specific extinction coefficients. Reported values are averages from HPLC and spectrophotometric methods. Total yield was obtained by multiplying the efficacies of all stages.

Thus, this approach opens up the possibility of efficient synthesis of both 3′- and 5′-GalNAc conjugates, and since all the reagents used are commercially available, this approach has the potential to scale to gram and potentially kilogram scales. And in the case of laboratory use, it makes it possible to build libraries for screening more quickly in order to optimize the fragment responsible for delivery.

In direct comparison with the examples in the literature, discrepancies were found with the efficiency of solid-phase conjugate synthesis [14], this may be explained by the use of various resins used in oligonucleotide synthesis, which additionally indicates the importance of this aspect for in-line conjugation, but the effectiveness of the described method, in any case, does not exceed the proposed method with post-synthetic liquid-phase conjugation.

When compared with other described methods of post-synthetic liquid-phase conjugation, in each case, a similar quantitative range of effectiveness is observed [11,14]. But the key difference is the use of RNAs modified with a terminal amino group. Despite the fact that this approach shows excellent results, in our case, an attempt was made to supplement synthetic tools with a simpler and cheaper method with the inversion of the nucleophilic group to the electrophilic one on the sequence itself.

In conclusion of the comparative analysis, it should be noted that, when assessing the contribution of the ligand moiety to the overall cost of GalNAc-oligonucleotide production, the specialized reagents used to introduce the tri-GalNAc unit represent one of the major cost drivers: at the laboratory scale, the indicative price of the starting amine—Tri-GalNAc(OAc)_3_·TFA is about USD 1800/g, whereas the ready-to-use building block for in-line 5′ conjugation—GalNAc-L96 phosphoramidite, is about USD 2700/g. Accordingly, a modular post-synthetic route employing the amine offers comparable practical feasibility while reducing the direct expenditure on the ligand component, and therefore can be considered a preferable strategy for lowering the cost of GalNAc-containing conjugates without altering the oligonucleotide portion.

### 3.4. Results of In Vitro Testing of Target GalNAc-Oligonucleotide Conjugates

In this study, two conjugates were selected from the library for in vitro evaluation: siPCSK9_1-C, which has the same sequence and chemical modifications as Inclisiran and is a 3′-GalNAc conjugate, and siPCSK9_2_12-C, which is a 5′-GalNAc conjugate. This design enabled a direct comparison with an established reference and allowed us to evaluate the effectiveness of the design and the reproducibility of the synthesis.

**Figure 11:**
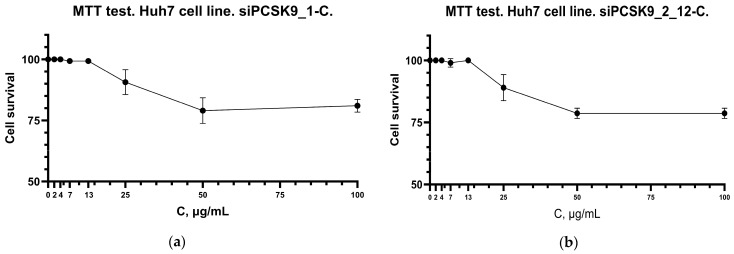
Cytotoxicity assessment of *PCSK9*-targeted siRNA conjugates in Huh7 cells using MTT assay. (**a**) MTT-test for siPSCK9_1-C. (**b**) MTT-test for siPSCK9_2_12-C.

A preliminary assessment of the cytotoxicity of the obtained siPCSK9_2_12-C complex on Huh7 cell cultures using the MTT test showed no significant reduction in cell viability in the studied concentration range, CC_50_ more than 100 µg/mL (Figure 11), which corresponds to the literature data on the safety of GalNAc conjugates [10,26]. Huh7 cells were chosen because they exhibit consistently high expression of the asialoglycoprotein receptor (ASGPR), making them a widely accepted standard model for preclinical evaluation of ASGPR-mediated uptake and thus enabling direct assessment of the impact of GalNAc conjugation on hepatocyte delivery. In addition, Huh7 cells actively express and secrete *PCSK9*, allowing reliable quantification of target knockdown at both the mRNA and protein levels; this cell line is broadly used in studies of *PCSK9* regulation and lipid metabolism, ensuring comparability of our results with the published literature. Further in vitro testing on the Huh7 cell line confirmed the specific biological activity of the studied complex, while its effectiveness was higher than that of siPCSK9_1-C. 

Evaluation of the *PCSK9* gene expression level by quantitative PCR (RT-qPCR) showed steady and increasing suppression of the mRNA of the target *PCSK9* gene over time (24, 48, and 72 h), which confirms the effectiveness of ASGPR-dependent cellular uptake provided by the GalNAc ligand.

Based on the RT–qPCR data, the unmodified sequences exhibited similar activity, suggesting that the observed differences are primarily attributable to sequence modifications, which are known to significantly influence siRNA silencing efficiency [20]. At the same time, the location of the GalNAc fragment at the 5’ end may further enhance silencing efficiency, which is consistent with previous findings [27].

Overall, the primary in vitro results indicate that the 5′-GalNAc–oligonucleotide conjugate siPCSK9_2_12-C is a promising therapeutic candidate for downregulating *PCSK9* expression and, consequently, for a potential reduction of LDL levels.

## 4. Materials and Methods

### 4.1. Materials

Commercially available reagents and solvents, unless otherwise stated, were purchased from either Sigma-Aldrich (Saint Louis, MO, USA), ACROS (Geel, Belgium) or Honeywell (Seelze, Germany) and were used as received. Reactions were monitored by thin-layer chromatography (TLC) carried out on Merck TLC silica gel plates (60 F254) (Merck, Darmstadt, Germany). Visualization of TLC plates was performed using UV light, 10% phosphomolybdic acid solution in ethanol, aqueous potassium permanganate or iodine fumes as staining reagents. Flash column chromatography purifications were carried out using 60 silica gel (particle size 0.040–0.063 mm). All reactions were carried out under an argon atmosphere unless otherwise stated.

All ^1^H and ^13^C NMR spectra were acquired using a Bruker Avance 400 spectrometer (Bruker BioSpin GmbH, Rheinstetten, Germany) with an operating frequency of 400 and 100 MHz, respectively, and calibrated using residual undeuterated chloroform (δH = 7.28 ppm) and CDCl_3_ (δC = 77.16 ppm), or undeuterated DMSO (δH = 2.50 ppm) and DMSO-d6 (δC = 39.51 ppm) as internal references. NMR data are presented as follows: chemical shift (δ ppm), multiplicity (s = singlet, d = doublet, t = triplet, q = quartet, m = multiplet, br. = broad), coupling constant in Hertz (Hz), integration.

Sequences of siRNA and corresponding conjugates high-resolution mass spectra (HRMS) were recorded on a Bruker maXis TOF mass spectrometer using electrospray ionization (ESI) and Bruker Microflex MALDI-TOF mass spectrometer (Bruker Daltonics GmbH, Bremen, Germany).

### 4.2. Oligonucleotide Synthesis Protocols

Oligonucleotide synthesis was performed on a 12-column DNA synthesizer (PolyGen GmbH, Langen, Germany) using a 1 µmol synthesis scale on Unylinker-functionalized polystyrene (PS) solid support (loading: 350 µmol/g).

The following solutions were used:

- Deblock: 3% (*w*/*v*) trichloroacetic acid (TCA) in anhydrous toluene
- Activator: 0.3 M 4,5-dicyanoimidazole (DCI) in anhydrous acetonitrile
- Cap A: 20% (*v*/*v*) 1-methylimidazole (NMI) in anhydrous acetonitrile
- Cap B: 30% (*v*/*v*) propionic anhydride and 30% (*v*/*v*) 2,6-lutidine in anhydrous acetonitrile
- Oxidation: 0.05 M iodine in pyridine/water (9:1, *v*/*v*)

Phosphoramidite solutions were prepared under an argon atmosphere in anhydrous acetonitrile at a concentration of 0.05 g/mL.

Synthesis was carried out using PolyGen GmbH high-quality protocol with DMT-ON mode to enable downstream purification with Glen-Pak RNA purification cartridges (Glen Research, Sterling, VA, USA). Cleavage from the solid support and base deprotection were achieved using AMA (1:1 *v*/*v* mixture of 32% aqueous ammonia and 40% aqueous methylamine) at 65 °C for 30 min.

For sequences containing a linker, cleavage was realized using 0.4 M NaOH in a mixture of EtOH-H2O (*v*/*v* = 4:1) at 80 °C for 5–10 min.

For RNA oligonucleotides containing 2′-O-TBDMS-protected phosphoramidites, the 2′-protecting groups were removed by triethylamine trihydrofluoride (TEA·3HF) treatment at 65 °C for 90 min.

The MALDI-TOF MS data for both the unmodified and modified oligonucleotide sequences are provided in the Supplementary Materials.

### 4.3. siRNA Design

Bioinformatic analysis of the *PCSK9* gene was performed in order to select candidate siRNA sequences. The *PCSK9* gene, associated with the development of atherosclerotic lesions and an increased risk of cardiovascular disease, was chosen as the target. siRNA design was implemented using the siDirect web application [28].

siDirect is a web application that selects siRNAs for a given mRNA transcript based on one or a combination of methods by K. Ui-Tei [29], A. Reynolds [8] and M. Amarzguioui [30]. Upon completion of the design, siDirect exports the data as a table containing 21-nucleotide 5′–3′-oriented guide (antisense) and passenger (sense) strands, indicating their localization on the target mRNA transcript, strand annealing temperatures and potential off-target effects of each siRNA.

The obtained data were collected and processed using a custom algorithm developed by our group. The algorithm employs built-in MS Excel formulas and a Google Apps Script to iteratively evaluate each siRNA sequence. Points are added to a predicted efficiency score when the following criteria are met: GC content of 36–52% (1 point), GC content in positions 2–7 of the antisense strand ≤ 19% and in positions 8–18 ≥ 52% (1 point), presence of an energy valley in positions 9–14 of the sense strand (2 points), GC repeats fewer than 3 times and AT repeats fewer than 4 times (1 point), TT overhang at the 3′-end (1 point), A or U at the 5′-end of the antisense strand (1 point), G or C at the 5′-end of the sense strand (1 point), A in position 6 of the antisense strand (1 point), A in positions 3 and 19 of the sense strand (1 point), absence of G or C in position 19 of the sense strand (1 point), absence of G in position 13 of the sense strand (1 point) and U in position 10 of the sense strand (1 point) [6].

Secondary structure formation probability and thermodynamic stability were further assessed using the Mfold algorithm (1 point) [31,32,33]. This criterion evaluates the likelihood of stable secondary structures within the mRNA target regions containing the complementary sequence. Off-target effects were evaluated using the NCBI BLAST algorithm (BLAST+ 2.17.0) (2 points) [34] with a homology threshold of ≤85%. This criterion reduces the frequency of off-target effects of the siRNA duplex, thereby minimizing undesired RNAi outcomes. Thus, siRNAs can achieve a maximum score of 15 points. Sequences scoring more than 10 points and showing favorable Mfold and NCBI BLAST results were considered as promising candidates (Supplementary Materials). Significant off-target effects are presented in the table below (Table 7).

The combination of the multiple oligonucleotide search and evaluation algorithms enables filtering of siRNA libraries such that approximately 80% of the sequences suppress target gene expression by more than 100-fold. To facilitate visualization of siRNA target sites on the mRNA transcript, the Clustal Omega alignment algorithm (v1.2.4), integrated into the Unipro UGENE software (v53.0) [35], was used. This approach significantly reduces the labor costs associated with validating siRNA efficacy, accelerates the identification of candidate molecules and optimizes the transition from in silico to in vitro testing, thereby reducing the burden on subsequent research and production stages.

### 4.4. siRNA Modification

In the given research we explore a novel method for the chemical modification of siRNAs. One siRNA duplex fully consisted of 2′OMe-substituted monomers. The other siRNA monomers were divided into 2 groups: high-energy monomers—G/C (designated as Y)—and low-energy monomers—A/U (designated as X). The non-terminal sequence of siRNAs was subdivided into triplets and modified into 3 variations as follows (Table 8).

### 4.5. Post-Synthetic Liquid-Phase GalNAc Conjugation

General procedure for 1 μmol scale:

NHS (50 eq.) and EDC (10 eq.) were added to a prepared solution of the oligonucleotide (1 eq.) in 600 μL DMSO–Acetonitrile (1:1, *v*/*v*). The mixture was left for 15 min, followed by adding amine (10 eq.). The reaction was stirred for an additional 2 h. Solvents were evaporated, and the residue was purified by AE IEX.

Chromatography purification was performed by using IEX chromatography Cytiva Akta explorer 100 (Cytiva, Uppsala, Sweden).

Column Cytiva Capto-HiresQ (Cytiva, Uppsala, Sweden).

Solid phase is YMC smartSep-Q30 (YMC Co., Ltd., Kyoto, Japan).

Liquid phase is as follows: Buffer A—0.01 M Tris, Buffer B—0.01 M Tris and 2 M Tris·HCl.

Program for purification:

- 2 cv of pure buffer A
- Sample addition
- 3 cv of pure buffer A
- 20 cv of gradient 0–100% B
- Column wash by 100% buffer B
- Column equilibration by pure buffer A

MALDI TOF MS data for synthesized GalNAc-oligonucleotide conjugates:

- siPCSK9_1-C-aS: calculated Mw (H+ form) = 7710.25, founded Mw = 7772.123
- siPCSK9_1-C-S: calculated Mw (H+ form) = 8657.57, founded Mw = 8631.495
- siPCSK9_2_12-aS: calculated Mw (H+ form) = 6889.34, founded Mw = 6873.91
- siPCSK9_2_12-S: calculated Mw (H+ form) = 8761.82, calculated Mw (Na salt) = 9221.82, founded Mw = 9006.2

### 4.6. Screening of Biological Activity of Unmodified siRNA Sequences

To test unmodified siRNA sequences, HEK293T cells (human embryonic kidney) in the amount of 100 thousand cells per well were seeded into a 24-well plate (SPL, Pocheon, Republic of Korea) in a complete DMEM nutrient medium (PanEco, Moscow, Russia) with the addition of 10% embryonic veal serum, 300 mg/L L-glutamine (PanEco, Moscow, Russia), 25 mM HEPES (PanEco, Moscow, Russia), 50 mcg/mL gentamicin (Gibco, Waltham, MA, USA), 10 mM sodium pyruvate (Servicebio, Wuhan, China). The tablet was incubated for a day at 37 °C in a 5% CO_2_ atmosphere. Next, the cells were transfected with a mixture of 0.2 micrograms of pVax-Kozak-Effluc-STOP-*PCSK9* plasmid, 0.4 micrograms of the tested siRNA and 1 microgram of the commercial reagent Lipofectamine 3000 (Thermo Fisher Scientific, Waltham, MA, USA) in a volume of 100 micrograms of OptiMem medium (Gibco, Waltham, MA, USA). Non-specific siRNA molecules were used as negative controls: a mixture of siMix (UTR regions of the hepatitis C virus genome) and siGFP (green fluorescent protein gene). siPCSK9_1 (the siRNA sequence of a commercial drug [15]) was used as a positive control. After incubation of the tablet at 37 °C in a 5% CO_2_ atmosphere for a day, the cells were lysed and luciferase activity was determined by luminescence intensity using a commercial reagent kit Bright-Glo™ Luciferase Assay System (Promega, Madison, WI, USA).

### 4.7. Screening of Biological Activity of Modified siRNA Sequences

To test the modified siRNA sequences, HEK293T cells (human embryonic kidney) in the amount of 100 thousand cells per well were seeded into a 24-well plate (SPL, Republic of Korea) in a complete DMEM nutrient medium (PanEco, Moscow, Russia) with the addition of 10% embryonic veal serum, 300 mg/L L-glutamine (PanEco, Moscow, Russia), 25 mM HEPES (PanEco, Moscow, Russia), 50 µg/mL gentamicin (Gibco, Waltham, MA, USA), 10 mM sodium pyruvate (Servicebio, Wuhan, China). The plate was incubated for a day at 37 °C in a 5% CO_2_ atmosphere. Next, the cells were transfected with a mixture of 0.2 µg of pVax-Kozak-Effluc-STOP-*PCSK9* plasmid, 0.4 µgof test siRNA and 1 mL of commercial reagent Lipofectamine 3000 (Thermo Fisher Scientific, Waltham, MA, USA) in a volume of 100 mL of OptiMem medium (Gibco, Waltham, MA, USA). Non-specific siRNA molecules were used as negative controls: a mixture of siMix (UTR regions of the hepatitis C virus genome) and siGFP (green fluorescent protein gene). An unmodified version of the siRNA sequence for the corresponding siPCSK9_2 and siPCSK9_6 series was used as a positive control.

After incubation of the plate at 37 °C in a 5% CO_2_ atmosphere for a day, the cells were lysed and luciferase activity was determined by luminescence intensity using a commercial reagent kit Bright-Glo™ Luciferase Assay System (Promega, Madison, WI, USA).

### 4.8. In Vitro Cytotoxicity of GalNAc-siRNA Conjugate

In preliminary experiments, the cytotoxicities of the GalNAc conjugate siPCSK9_2_12-C and the modified siRNA duplex were studied in a wide range of concentrations from 100 to 2 µg/mL. For this purpose, HEK293T and Huh-7 cells in the amount of 35,000 cells/well were seeded into a 96-well plate (SPL, Republic of Korea) in 100 µL of complete DMEM nutrient medium (PanEco, Moscow, Russia) with the addition of 10% fetal veal serum, 300 mg/L of L-glutamine (PanEco, Moscow, Russia), 25 mM of HEPES (PanEco, Moscow, Russia), 50 µg/mL of gentamicin (Gibco, Waltham, MA, USA), 10 mM of sodium pyruvate (Servicebio, Wuhan, China). The tablet was incubated for a day at 37 °C in a 5% CO_2_ atmosphere to achieve 75% confluence. Next, the corresponding amounts of synthesized siRNAs were added at the indicated concentrations, after which they were titrated by transferring 100 µL of medium from a well with a concentration of 100 µg/mL to the final wells with a concentration of 2 µg/mL. Subsequently, the tablet was incubated for a day at 37 °C in a 5% CO_2_ atmosphere. The next day, 25 µL of MTT solution (NeoFroxx, Einhausen, Germany) with a concentration of 4 mg/mL was added to each well and the tablet was incubated for 4 h. The reaction was stopped by adding a 20% sodium dodecyl sulfate (SDS) solution (BioFroxx, Einhausen, Germany), followed by incubation. The next day, the optical density of the samples was measured on a spectrophotometer (Multiskan GO, Thermo Scientific, Waltham, MA, USA) at wavelengths of 570 nm and 650 nm. Based on the data obtained, the CC_50_ value was calculated.

### 4.9. Study of the Biological Activity of the GalNac-siRNA Complex in In Vitro Experiments

To test the studied complex, cells of the Huh-7 line (human hepatocellular carcinoma) in the amount of 50, 60 and 80 thousand cells per well (for experimentation on the first, second and third days, respectively) were seeded into a 24-well plate (SPL, Republic of Korea) in a complete DMEM/F12 nutrient medium (PanEco, Moscow, Russia) with the addition of 10% fetal veal serum, 300 mg/L of L-glutamine (PanEco, Moscow, Russia), 25 mM of HEPES (PanEco, Moscow, Russia), 50 µg/mL of gentamicin (Gibco, Waltham, MA, USA), 10 mM of sodium pyruvate (Servicebio, Wuhan, China). The tablet was incubated for a day at 37 °C in a 5% CO_2_ atmosphere. The GalNAc conjugated complex (siPCSK9_2_12-C) was dissolved in a sterile phosphate-salt buffer solution and a stock solution with a concentration of 0.1 mg/mL was prepared. Next, the calculated amounts of the siPCSK9_2_12-C and siPCSK9_1-C complexes were added to the wells with a full medium so that their amount was 1 µM per well, respectively. The cells were incubated for the entire duration of the experiment (24 h, 48 h, 72 h) at 37 °C in a 5% CO_2_ atmosphere. Next, the cells were lysed in Lysis Buffer (Thermo Fisher Scientific, Waltham, MA, USA). The total RNA from the cells was isolated using the RNeasy Mini Kit (Qiagen, Hilden, Germany) in accordance with the manufacturer’s recommendation. The RNA concentration in the samples was measured using a NanoDrop spectrophotometer (Thermo Fisher Scientific, Waltham, MA, USA). cDNA synthesis was performed using the Reverta-L kit (AmpliSens, Moscow, Russia) in accordance with the manufacturer’s recommendations. PCR amplification was performed on a DTprime device (DNA Technology, Moscow, Russia) using a SYBR Green PCR mix (Eurogen, Moscow, Russia) and primers directed to the human *PCSK9* gene. PCR amplification was performed according to the following temperature program (Table 9).

The beta-actin gene ACTB was used as a normalizing gene in the study of *PCSK9* expression (Table 10). The relative expression level (RQ) was calculated by ΔΔCq.

The results of the PCR were evaluated using the standard ΔΔCq method for analyzing gene expression data. The ACTB gene was selected as a housekeeping gene; the target gene was normalized to the housekeeping gene according to Formula (1). Then, the difference between the average ΔCt of the experimental group and the average ΔCt of the control group was evaluated according to Formula (2). Finally, fold change was calculated using Formula (3) [36].

(1) $$∆Ct=Ct\left(target\;gene\right)-Ct(housekeeping\;gene)$$

Formula (1). ∆Ct—difference between the number of amplification cycles, $Ct\left(target\;gene\right)$—number of amplification cycles for the target gene, $Ct\left(housekeeping\;gene\right)$—the number of amplification cycles for the housekeeping gene.

(2) $$∆∆Ct=∆Ct\left(experimental\;group\right)-∆Ct(Control\;group)$$

Formula (2). ∆∆Ct—difference between the average ∆Ct of experimental and control groups, $∆Ct\left(experimental\;group\right)$—the average ∆Ct for the experimental group, $∆Ct\left(Control\;group\right)$—the average ∆Ct for the control group

(3) $$Fold\;change=2^{-(∆∆Ct)}$$

Formula (3). ∆∆Ct—difference between the average ∆Ct of the experimental and control groups.

## 5. Conclusions

Ultimately, within the framework of this study, experimental validation of the algorithm we used was carried out, new modification patterns were proposed that could be adapted into the algorithm, a largely simple and effective method for creating ligand-oligonucleotide conjugates was integrated, and in confirmation of this, a new therapeutic candidate for hypercholesterolemia was obtained. Future stages will include in vivo testing to assess specific activity against human *PCSK9*, pharmacokinetic and pharmacodynamic profiles, as well as assessment of toxicity and immunogenicity.

## Figures and Tables

**Table 1:** Nucleotide sequences and in vitro activity of the top 5 modified siRNA candidates from the siPCSK9_2_X and siPCSK9_6_X series targeting human PCSK9.

Name		Sequence 5′-3′	Relative Luciferse Activity ^1^
siPCSK9_2 (unmodified)	aS	rArUrArArArUrGrUrCrUrGrCrUrUrGrCrUrUrGrGrG	0.2706 ± 0.0322
	S	rCrArArGrCrArArGrCrArGrArCrArUrUrUrArUrCrU	
siPCK9-2-12 ^2^	aS	mAfUmAfAmAfUmGmUmCmUmGfCmUfUmGfCmUfUmGfGmG	0.0628 ± 0.0338
	S	fCmAmAmGmCmAmAmGmCmAfGfAfCmAmUmUmUmAmUmCmU	
siPCSK9-2-4	aS	mAfUfAfAmAmUmGmUmCmUmGmCmUmUmGmCmUfUfGfGmG	0.1303 ± 0.0247
	S	mCmAmAmGmCmAmAmGmCmAfGfAfCmAmUmUmUmAmUmCmU	
siPCSK9-2-1	aS	mAmUmAmAmAmUmGmUmCmUmGmCmUmUmGmCmUmUmGmGmG	0.1358 ± 0.0369
	S	mCmAmAmGmCmAmAmGmCmAmGmAmCmAmUmUmUmAmUmCmU	
siPCSK9-2-10	aS	mAmUmAmAmAmUmGmUfCfUfGmCmUmUmGmCmUmUmGmGmG	0.1368 ± 0.0245
	S	mCmAmAmGmCmAmAmGmCmAfGfAfCmAmUmUmUmAmUmCmU	
siPCSK9-2-3	aS	mAfUmAfAmAfUmGfUmCmUmGfCmUfUmGfCmUfUmGfGmG	0.1383 ± 0.0245
	S	mCmAmAmGmCmAmAmGmCmAfGfAfCmAmUmUmUmAmUmCmU	
siPCSK9_6 (unmodified)	aS	rUrArArArUrGrUrCrUrGrCrUrUrGrCrUrUrGrGrGrU	0.3249 ± 0.1326
	S	rCrCrArArGrCrArArGrCrArGrArCrArUrUrUrArUrC	
siPCSK9-6-1	aS	mUmAmAmAmUmGmUmCmUmGmCmUmUmGmCmUmUmGmGmGmU	0.0334 ± 0.0312
	S	mCmCmAmAmGmCmAmAmGmCmAmGmAmCmAmUmUmUmAmUmC	
siPCSK9-6-9	aS	mUfAmAmAmUfGmUfCmUmGmCfUmUfGmCmUmUmGmGfGmUmAmA	0.0574 ± 0.0368
	S	mCmCmAmAmGmCmAmAmGmCfAfGfAmCmAmUmUmUmAmUmC	
siPCSK9-6-5	aS	mUfAmAfAfUfGmUmCmUmGmCmUmUmGmCfUfUfGmGfGmU	0.1192 ± 0.0593
	S	mCmCmAmAmGmCmAmAmGmCfAfGfAmCmAmUmUmUmAmUmC	
siPCSK9-6-3	aS	mUfAmAfAmUfGmUfCmUmGmCfUmUfGmCfUmUfGmGfGmU	0.1224 ± 0.0603
	S	mCmCmAmAmGmCmAmAmGmCfAfGfAmCmAmUmUmUmAmUmC	
siPCSK9-6-7	aS	mUmAmAmAfUfGfUmCmUmGmCmUfUfGfCmUmUmGmGmGmU	0.1617 ± 0.0731
	S	mCmCmAmAmGmCmAmAmGmCfAfGfAmCmAmUmUmUmAmUmC	

^1^ Fraction of control, mean ± σ, N = 7; ^2^ this sequence was selected for conjugate formation.

**Table 2:** Conditions of cleavage of the conjugate from the solid phase.

Reagent	Conditions	Product
Ammonium hydroxide	55 °C/2 h	Desired product *
	r.t./12 h	Desired product
AMA ^1^	65 °C/30 min	Desired product **
0.05 M K_2_CO_3_ in EtOH-H_2_O (*v*/*v* = 1:1)	r.t./18 h	Desired product
	r.t./24 h	Desired product **

^1^ AMA (ammonium hydroxide 32%/40% aqueous methylamine; *v*/*v* = 1:1); * low level cleavage from solid phase; ** partial or complete degradation of the conjugate was observed.

**Table 3:** Conditions for the removal of modified oligonucleotides from the solid phase.

Reagent	Temperature	Time	Product
AMA ^1^	55 °C	30 min	Benzylic ester or amide (major)/acid (minor)
Ammonium hydroxide	r.t.	6 h *	-
		12 h **	Acid (major)/benzylic ester or amide(minor)
	55 °C	6 h **	Acid (major)/benzylic ester or amide (minor)
		12 h	Benzylic ester or amide (major)/acid (minor)
0.05 M K_2_CO_3_ EtOH-H_2_O (*v*/*v* = 1:1)	r.t.	2–12 h *	-
		24 h **	Benzylic ester
	55 °C	8–12 h **	Benzylic ester
		24 h	Benzylic ester
0.4 M NaOH EtOH-H_2_O (*v*/*v* = 4:1)	80 °C	5–10 min	Acid

^1^ AMA (ammonium hydroxide/40% aqueous methylamine; *v*/*v* = 1:1); * the product was not observed; ** low conversion of solid-phase cleavage.

**Table 4:** Conditions of liquid-phase conjugation.

Reagents	Conditions	Yield
CDI THF-DMF	r.t./2 h	Desired product was not observed
HBTU, DIPEA DMF-MeCN	r.t./30 min	Trace
EDC·HCl DMSO-MeCN	r.t./2 h	Trace
EDC/NHS DMSO-MeCN	r.t./ 2 h	70–95% *

* Efficacy was calculated by data from HPLC, MALDI-TOF MS and Bouguer–Lambert–Beer law.

**Table 5:** Additional criteria for selecting sequences.

AG/UC ~50%
Distribution of AU and GC by islets of 2–3 nucleotides
The presence of a 4–6-mer sequence A/U or G/C
The first and last 2 nucleotides are A/U and G/C
If the first 2 nucleotides are A/U, 3–4 are C/G
If the first 2 nucleotides are G/C, 3–4–A/U

**Table 6:** Comparison of the efficiency of in-line solid-phase conjugation and out-line post-synthetic liquid-phase conjugation.

Stage	In-Line Conjugation		Out-Line Conjugation	
	3′-End	5′-End	3′-End	5′-End
1. Solid-phase synthesis	Synthesis of 22 repeating cycles, including the conjugation stage		Synthesis of 22 repetitive cycles, including the stage of introducing modification for PSLPC ^1^	
Efficacy (1)	49% *	37% *	>90% *	
2. Chromatography	AE IEX chromatography of DMT-off product		Reverse phase chromatography of DMT-on product on GlenPak cartridges	
Efficacy (2)	55% *		>95% *	
3. Desalting	SEC chromatography		Not required	
Efficacy (3)	>95% *		–	
4. Post-synthetic liqud-phase conjugation	Not required		EDC/NHS, DMSO-MeCN, 2 h., r.t.	
Efficacy (4)	–		70–95% *	
5. Chromatography	Not required		AE IEX chromatography of conjugate	
Efficacy (5)	–		>90% *	
6. Desalting	Not required		SEC chromatography	
Efficacy (6)	–		>95% *	
Total yield	26%	19% **	56–81%	

^1^ PSLPC—post-synthetic liquid-phase conjugation; * efficacy was calculated by data from HPLC, MALDI-TOF MS and Bouguer–Lambert–Beer law; ** the total efficacy described in the literature for this approach was 47% [14].

**Table 7:** Significant off-target effects for siPCSK9_N.

Duplex	AS Off-Target, Gene (% Homology)	S Off-Target, Gene (% Homology)
siPCSK9_2	NG_047156.1 (90%),	NG_021195.2 (81%),
	NG_012232.1 (90%)	NG_012971.2 (81%)
siPCSK9_6	NG_029777.2 (95%),	NG_009191.3 (95%),
	NG_051831.2 (95%),	NG_047156.1 (90%)
	NG_012232.1 (90%)	

**Table 8:** Variants of modification patterns.

Case	Set 1 Antisense Even Modification Variants	Set 2 Antisense Odd Modification Variants	Sense
XXX	mXmXfX	mXfXmX	fXmXmX
YYY	mYmYfY	mYfYmY	fYmYmY
XYX	mXfYmX	fXmYfX	fXfYmX
YXY	fYmXfY	mYfXmY	mYmXfY
5′XY	5′mXfY	5′fXmY	5′fXfX
5′YX	5′mYfX	5′fYmX	5′fYfX
5′XX	5′mXmX	5′fXfX	5′mXfX
3′XY	3′mXfY	3′fXmY	3′mXfX
3′YX	3′fYmX	3′mYfX	3′mYmX
3′XX	3′fXfX	3′mXmX	3′fXmX
3′YY	3′mYmY	3′fYfY	3′fYmY

**Table 9:** Temperature program of PCR amplification.

Stage Number	Temperature	Time	Number of Cycles
1	95.0 °C	300 s.	1
2	95.0 °C	30 s.	40
	65.2 °C *	15 s.	
	72.0 °C	30 s.	

* The stage with fluorescence.

**Table 10:** Sequences of primers.

Target Gene	Primers/ Probes	Sequence (5′-3′)
*ACTB*	F	CATCACCATTGGCAATGAG
	R	CACACTTCATGATGGAGTTGAAG
	Z	(ROX)CTTCCTTCCTGGGCATGGAGTCCTGTG(RTQ2)
*PCSK9*	F	GGTGTATCTCCTAGACACCAGCATAC
	R	GGAGTAGAGGCAGGCATCGT

## Data Availability

The original contributions presented in this study are included in the article/Supplementary Material. Further inquiries can be directed to the corresponding author(s).

## Supplementary Materials

The following supporting information can be downloaded at: https://www.mdpi.com/article/10.3390/molecules31030476/s1.

## Abbreviations

The following abbreviations are used in this manuscript:

AE IEX	Anion Exchange Ion Exchange Chromatography
ASGPR	Asialoglycoprotein Receptor
ASO	Antisense Oligonucleotide
BOP	Benzotriazol-1-yloxy-tris(dimethylamino)phosphonium hexafluorophosphate
cDNA	Complementary DNA
CDI	Carbonyldiimidazole
DCM	Dichloromethane
DIPEA	N,N-Diisopropylethylamine
DMF	Dimethylformamide
DMSO	Dimethyl Sulfoxide
DMT	Dimethoxytrityl
DMT-off synthesis	Oligonucleotide synthesis with the final 5′-DMT group removed on the synthesizer
DMT-on synthesis	Oligonucleotide synthesis with the final 5′-dimethoxytrityl (DMT) group retained.
EDC	1-Ethyl-3-(3-dimethylaminopropyl)carbodiimide
EtOH	Ethanol
GalNAc	N-Acetylgalactosamine
HBTU	O-(Benzotriazol-1-yl)-N,N,N’,N’-tetramethyluronium hexafluorophosphate
HPLC	High-Performance Liquid Chromatography
LDL	Low-Density Lipoprotein
MeCN	Acetonitrile
mRNA	Messenger ribonucleic acid (Messenger RNA)
miRNA	Micro ribonucleic acid (microRNA)
NHS	N-Hydroxysuccinimide
PA	Phosphoramidite
qRT-PCR	Quantitative Reverse Transcription Polymerase Chain Reaction
RISC	RNA-Induced Silencing Complex
ROX	Carboxy-X-Rhodamine
RP HPLC	Reversed-Phase High-Performance Liquid Chromatography
RQ	Relative Quantity
RTQ-2	Real Time Quencher-2
siRNA	Small Interfering RNA
THF	Tetrahydrofuran
