# Supplementary material for: Refined Design and Liquid-Phase Assembly of GalNAc-siRNA Conjugates: Comparative Efficiency Validation in PCSK9 Targeting
Source: Molecules. 2026 Jan 29;31(3):476. doi: 10.3390/molecules31030476 (PMC12899625; doi:10.3390/molecules31030476)
Supplement: Supplementary file 1 [file molecules-31-00476-s001.zip › Supplementary Materials Figures.pdf]

siPSCK9_1

| m/z | Rel. Intens. | Area | SN |
|---|---|---|---|
| 2592.337 | 15 | 19147 | 7.6 |
| 3196.628 | 9 | 9927 | 4.8 |
| 3702.961 | 58 | 66477 | 33.5 |
| 3840.474 | 22 | 27044 | 12.9 |
| 4209.606 | 8 | 9962 | 5.3 |
| 7415.966 | 100 | 238484 | 103.5 |
| 7693.025 | 33 | 92858 | 34.6 |
| 14931.208 | 3 | 2296 | 5.6 |
| 15202.977 | 3 | 1799 | 5.9 |

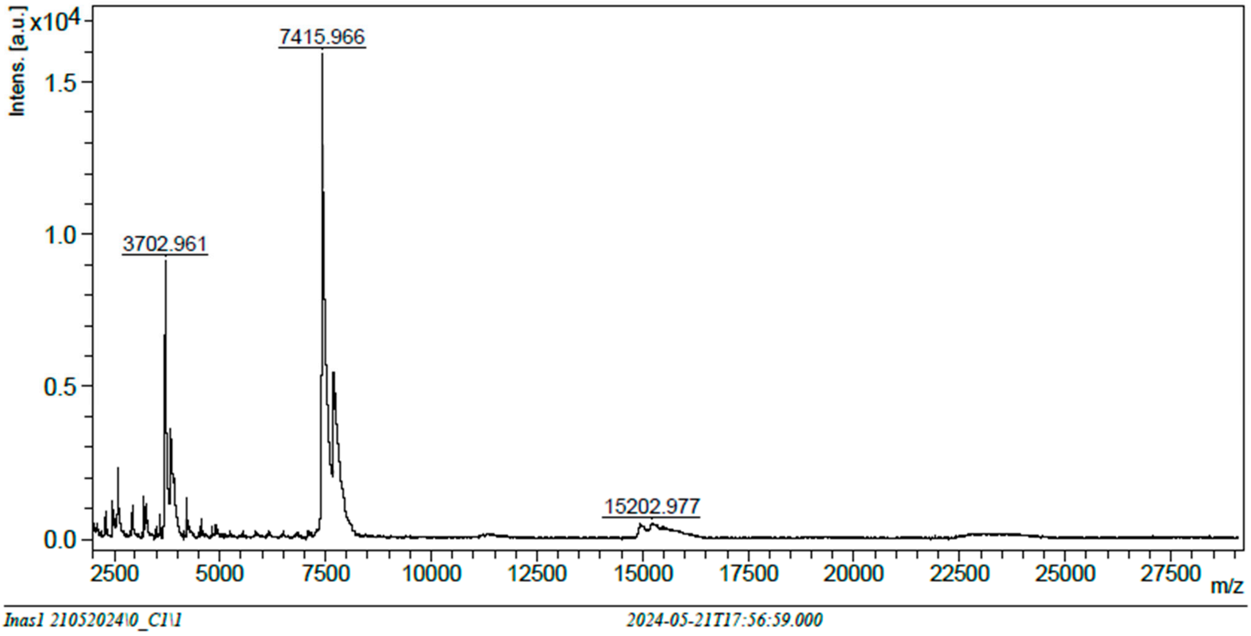


**Figure S1.** MALDI-TOF MS spectrum of siPCSK9_1-aS.

| Name | Sequence (5′-3′) |
|---|---|
| siPSCK9_1-aS | rArCrArArArArGrCrArArArArCrArGrGrUrCrUrArGrArA |
| Calculated Mw (H+ form) | 7431.64 |
| Founded Mw | 7416.0 |

siPSCK9_1

| m/z | Rel. Intens. | Area | SN |
|---|---|---|---|
| 2180.909 | 9 | 17604 | 11.8 |
| 3268.991 | 9 | 91736 | 55.6 |
| 6551.578 | 100 | 555604 | 230.4 |
| 6827.880 | 11 | 76474 | 26.9 |
| 13161.740 | 2 | 1600 | 9.6 |

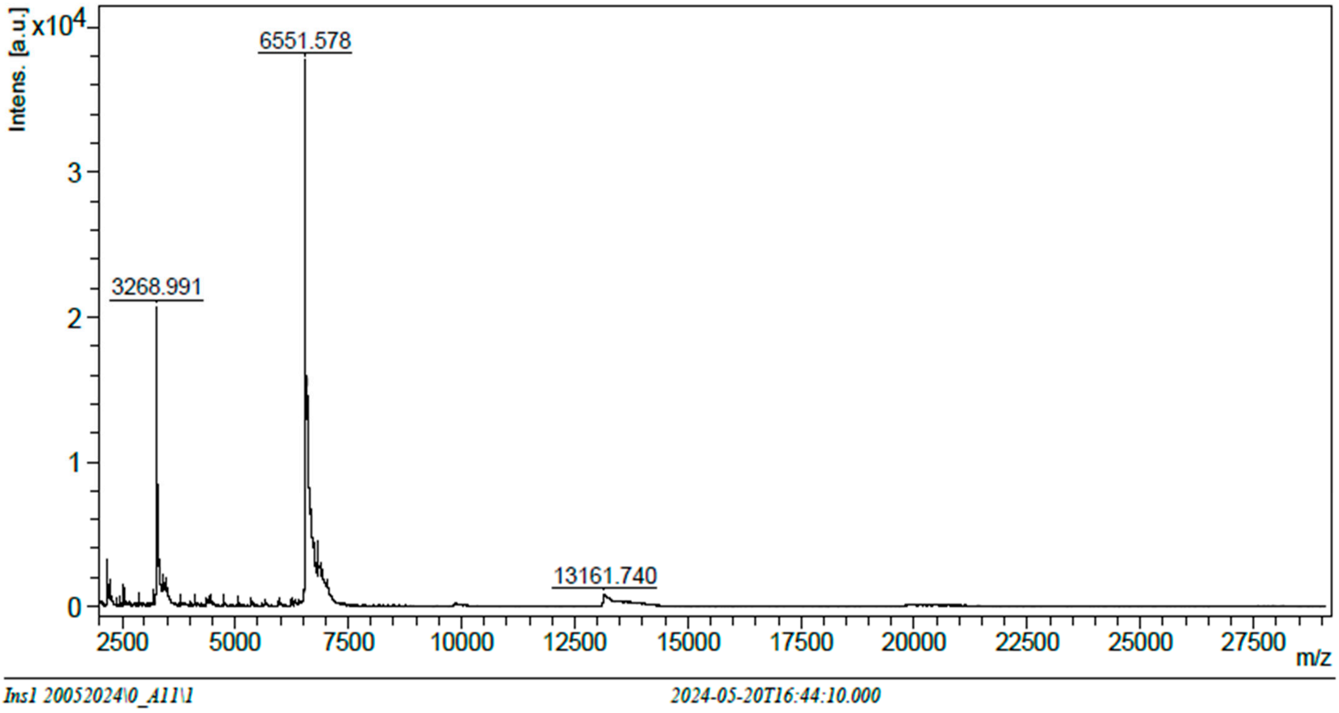


**Figure S2.** MALDI-TOF MS spectrum of siPCSK9_1-S.

| Name | Sequence (5′-3′) |
|---|---|
| siPSCK9_1-S | rCrUrArGrArCrCrUrGrUdTrUrUrGrCrUrUrUrUrGrU |
| Calculated Mw (H+ form) | 6565.87 |
| Founded Mw | 6551.6 |

siPSCK9_2

| m/z | Rel. Intens. | Area | SN |
|---|---|---|---|
| 2222.618 | 18 | 20720 | 16.4 |
| 3331.011 | 9 | 98141 | 35.3 |
| 3468.423 | 2 | 18591 | 8.2 |
| 6677.399 | 100 | 328697 | 169.1 |
| 6953.599 | 25 | 87326 | 43.5 |
| 13406.991 | 1 | 835 | 5.1 |
| 13717.749 | 1 | 1378 | 5.0 |

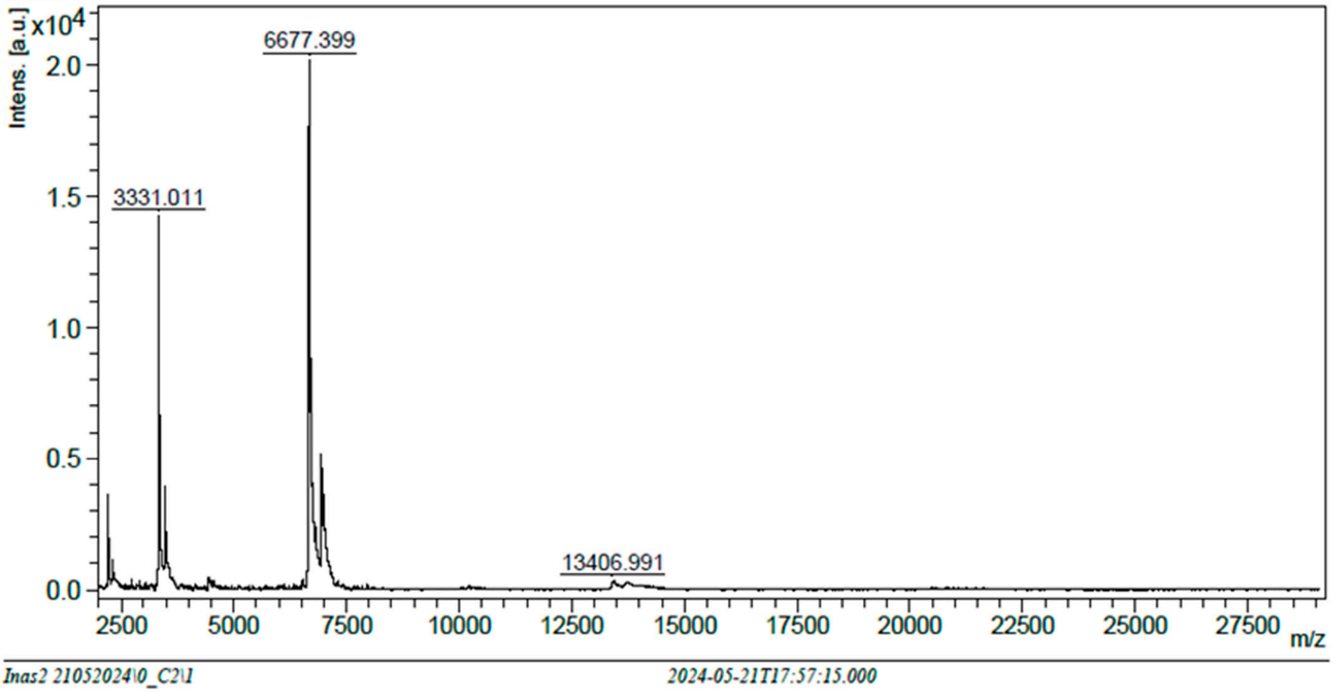


**Figure S3.** MALDI-TOF MS spectrum of siPCSK9_2-aS

| Name | Sequence (5′-3′) |
|---|---|
| siPSCK9_2-aS | rArUrArArArUrGrUrCrUrGrCrUrUrGrCrUrUrGrGrG |
| Calculated Mw (H+ form) | 6691.01 |
| Founded Mw | 6677.4 |

siPSCK9_2

| m/z | Rel. Intens. | Area | SN |
|---|---|---|---|
| 2215.093 | 5 | 495 | 4.3 |
| 3321.039 | 6 | 7386 | 15.7 |
| 3508.001 | 5 | 324 | 4.5 |
| 6652.561 | 100 | 50463 | 110.4 |
| 6928.668 | 9 | 4454 | 10.3 |
| 7026.790 | 7 | 3108 | 8.0 |
| 13385.066 | 2 | 344 | 2.8 |

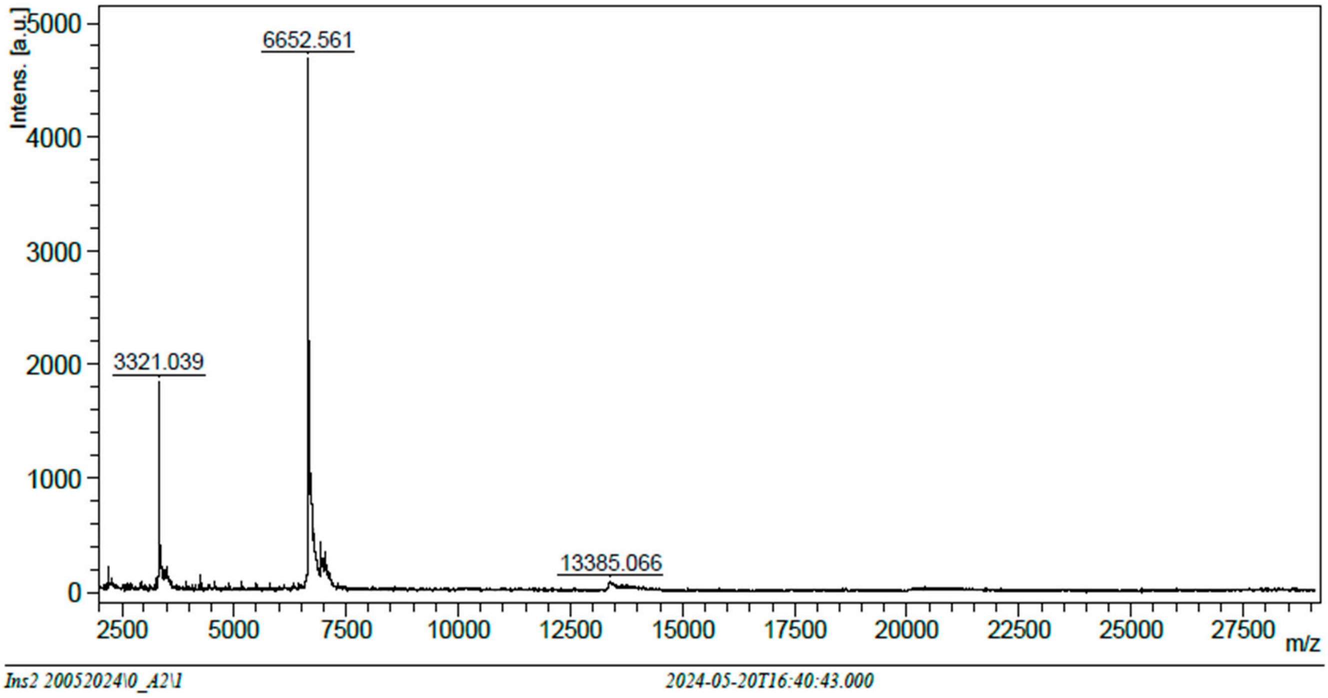


**Figure S4.** MALDI-TOF MS spectrum of siPCSK9_2-S.

| Name | Sequence (5′-3′) |
|---|---|
| siPSCK9_2-s2 | rCrArArGrCrArArGrCrArGrArCrArUrUrUrArUrCrU |
| Calculated Mw (H+ form) | 6664.08 |
| Founded Mw | 6652.6 |

siPSCK9_3

| m/z | Rel. Intens. | Area | SN |
|---|---|---|---|
| 2270.623 | 6 | 8001 | 6.6 |
| 3404.128 | 6 | 66222 | 26.9 |
| 3541.510 | 2 | 16184 | 9.9 |
| 6822.391 | 100 | 285358 | 180.6 |
| 7098.659 | 33 | 99096 | 60.3 |
| 13714.523 | 2 | 2807 | 5.7 |
| 13974.139 | 2 | 1417 | 5.3 |

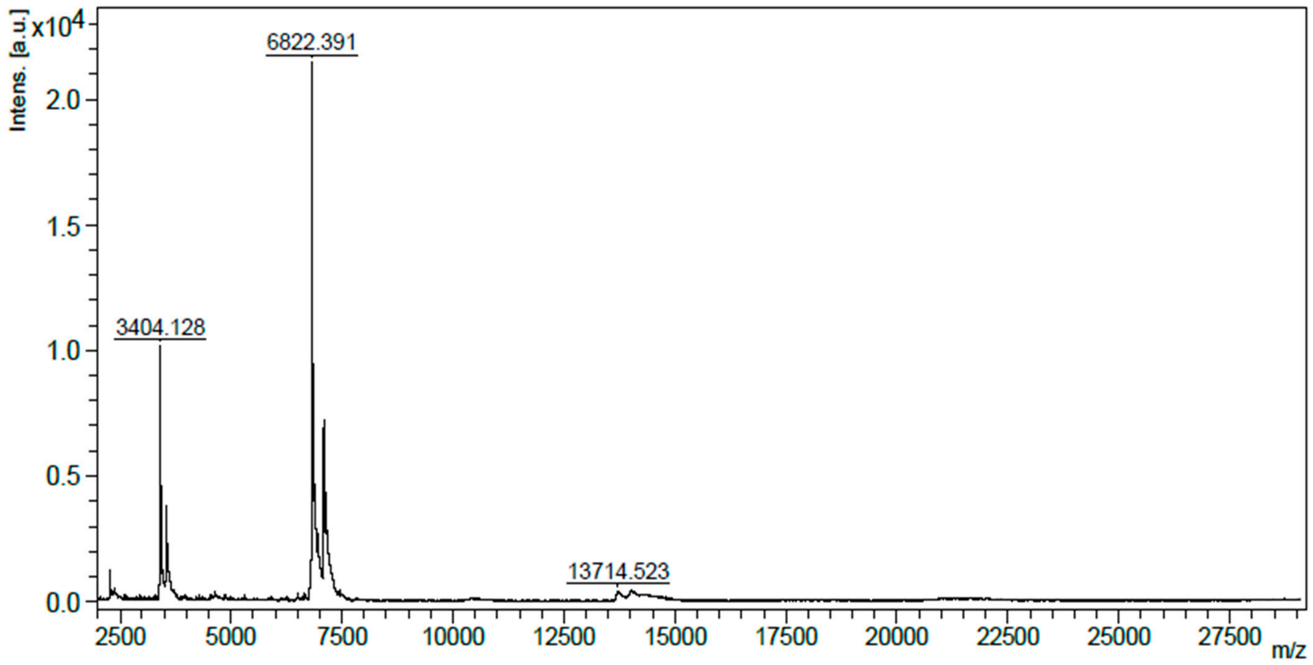


Inas3 21052024\0_C3\1 2024-05-21T17:57:35.000

**Figure S5.** MALDI-TOF MS spectrum of siPCSK9_3-aS

| Name | Sequence (5′-3′) |
|---|---|
| siPSCK9_3-aS | rUrGrArArGrUrArArGrArArGrArGrGrCrUrUrGrGrC |
| Calculated Mw (H+ form) | 6839.2 |
| Founded Mw | 6822.4 |

siPSCK9_3

| m/z | Rel. Intens. | Area | SN |
|---|---|---|---|
| 2156.916 | 9 | 6731 | 12.7 |
| 3232.479 | 8 | 60720 | 35.8 |
| 3252.178 | 5 | 18110 | 27.7 |
| 6476.189 | 100 | 253132 | 212.2 |
| 6803.749 | 6 | 8448 | 14.0 |
| 9747.735 | 1 | 548 | 3.3 |
| 13007.284 | 3 | 3313 | 10.6 |

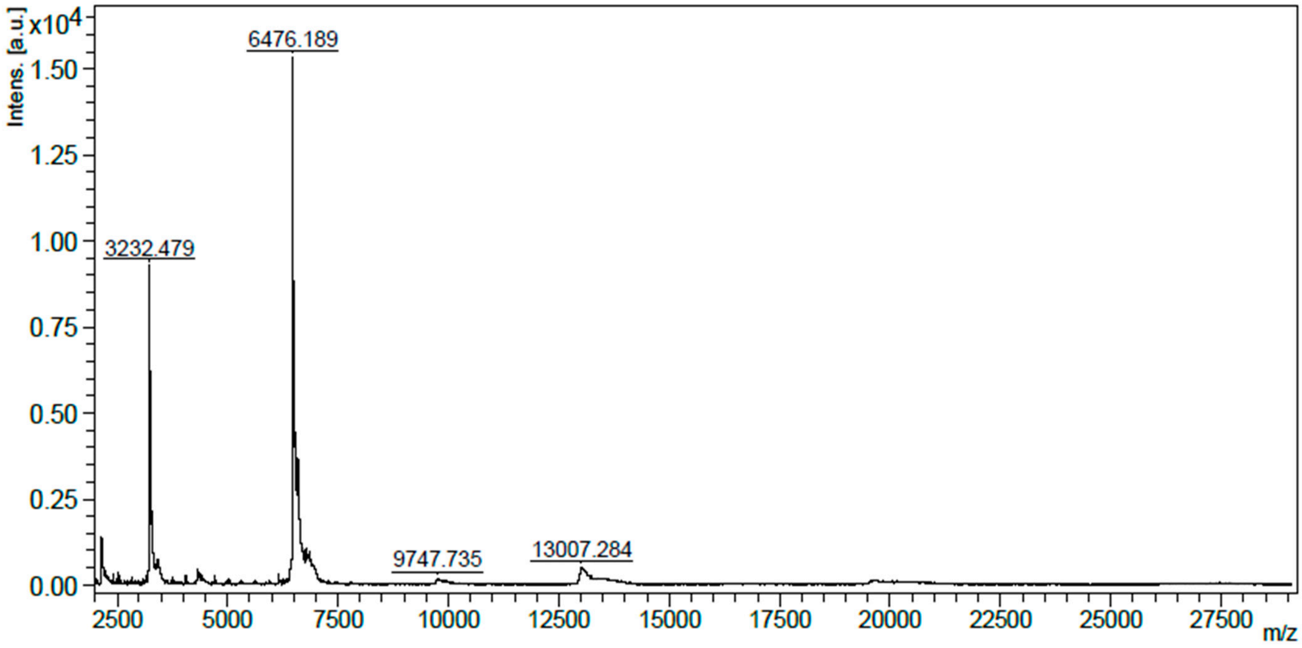


Ins3 20052024\0_A3\1 2024-05-20T16:41:02.000

**Figure S6.** MALDI-TOF MS spectrum of siPCSK9_3-S.

| Name | Sequence (5′-3′) |
|---|---|
| siPSCK9_3-S | rCrArArGrCrCrUrCrUrUrCrUrUrArCrUrUrCrArCrC |
| Calculated Mw (H+ form) | 6489.9 |
| Founded Mw | 6476.2 |

siPSCK9_4

| m/z | Rel. Intens. | Area | SN |
|---|---|---|---|
| 2251.244 | 15 | 10381 | 8.8 |
| 3377.295 | 65 | 62013 | 43.6 |
| 3514.534 | 17 | 16765 | 11.9 |
| 6760.612 | 100 | 181351 | 97.6 |
| 7036.781 | 24 | 46080 | 23.9 |

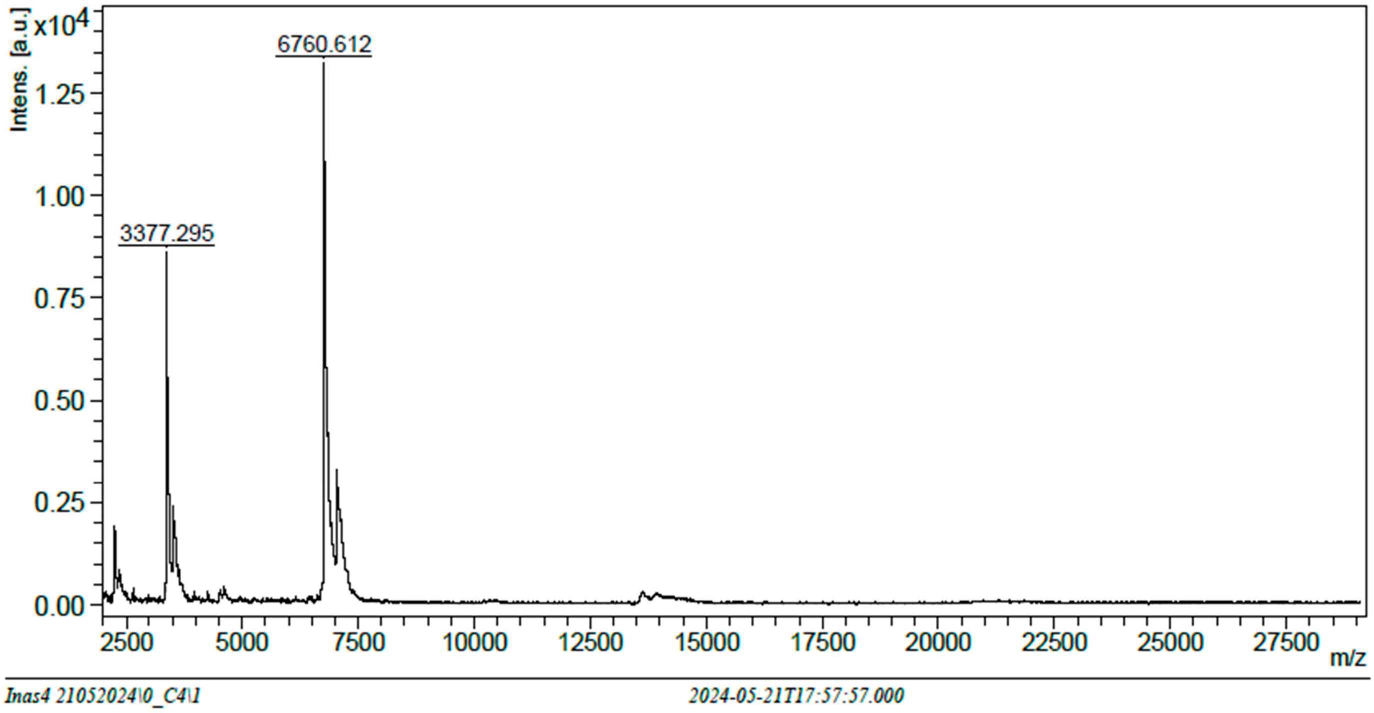


**Figure S7.** MALDI-TOF MS spectrum of siPCSK9_4-aS

| Name | Sequence (5′-3′) |
|---|---|
| siPSCK9_4-aS | rUrArArGrArArGrArGrGrCrUrUrGrGrCrUrUrCrArG |
| Calculated Mw (H+ form) | 6776.13 |
| Founded Mw | 6760.6 |

siPSCK9_4

| m/z | Rel. Intens. | Area | SN |
|---|---|---|---|
| 2190.511 | 42 | 17202 | 12.2 |
| 2298.714 | 39 | 22053 | 11.5 |
| 2626.950 | 36 | 18453 | 11.4 |
| 2971.150 | 23 | 12436 | 7.5 |
| 3282.892 | 27 | 113060 | 27.7 |
| 3579.923 | 27 | 18987 | 9.4 |
| 3885.393 | 20 | 11885 | 7.2 |
| 4189.593 | 19 | 10905 | 7.3 |
| 6571.654 | 6 | 70539 | 13.5 |
| 6693.274 | 100 | 117816 | 53.8 |
| 6852.814 | 26 | 15434 | 14.1 |
| 13214.487 | 6 | 942 | 5.8 |

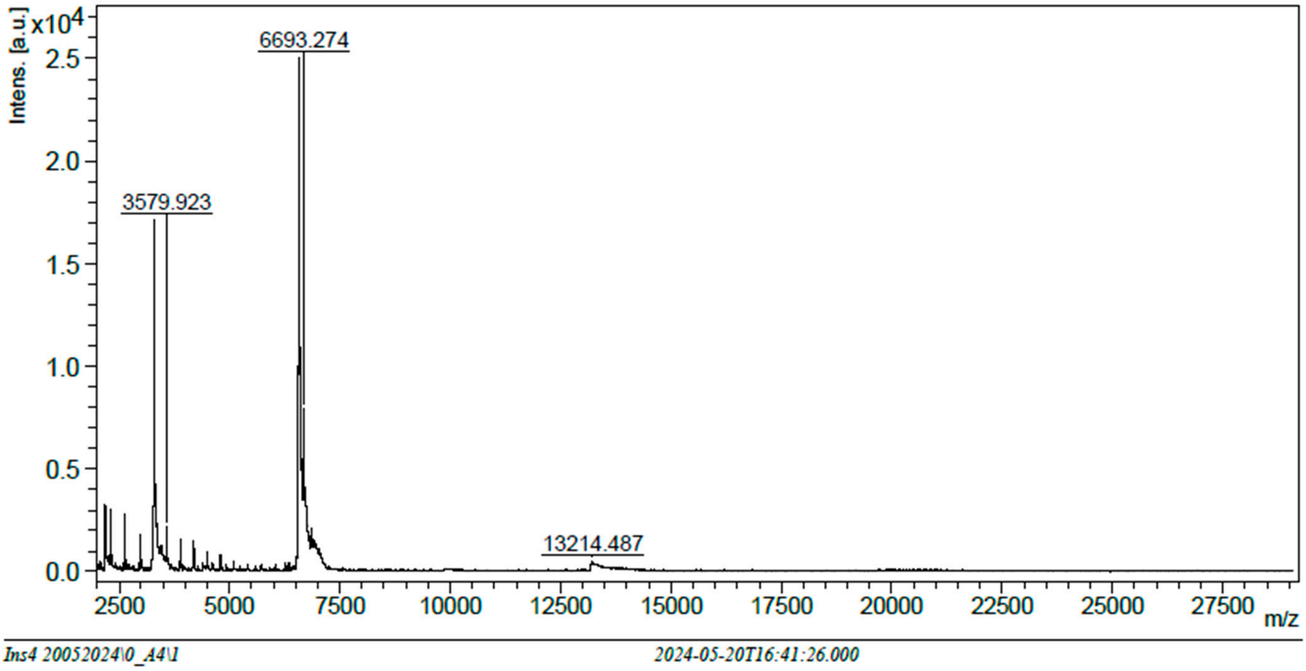


**Figure S8.** MALDI-TOF MS spectrum of siPCSK9_4-S.

| Name | Sequence (5′-3′) |
|---|---|
| siPSCK9_4-S | rGrArArGrCrCrArArGrCrCrUrCrUrUrCrUrUrArCrU |
| Calculated Mw (H+ form) | 6592.99 |
| Founded Mw | 6693.3 |

siPSCK9_5

| m/z | Rel. Intens. | Area | SN |
|---|---|---|---|
| 2271.579 | 15 | 9407 | 8.5 |
| 3407.391 | 100 | 75419 | 62.4 |
| 3544.696 | 36 | 30279 | 22.6 |
| 6815.794 | 5 | 31377 | 41.0 |
| 7091.470 | 1 | 6705 | 14.3 |

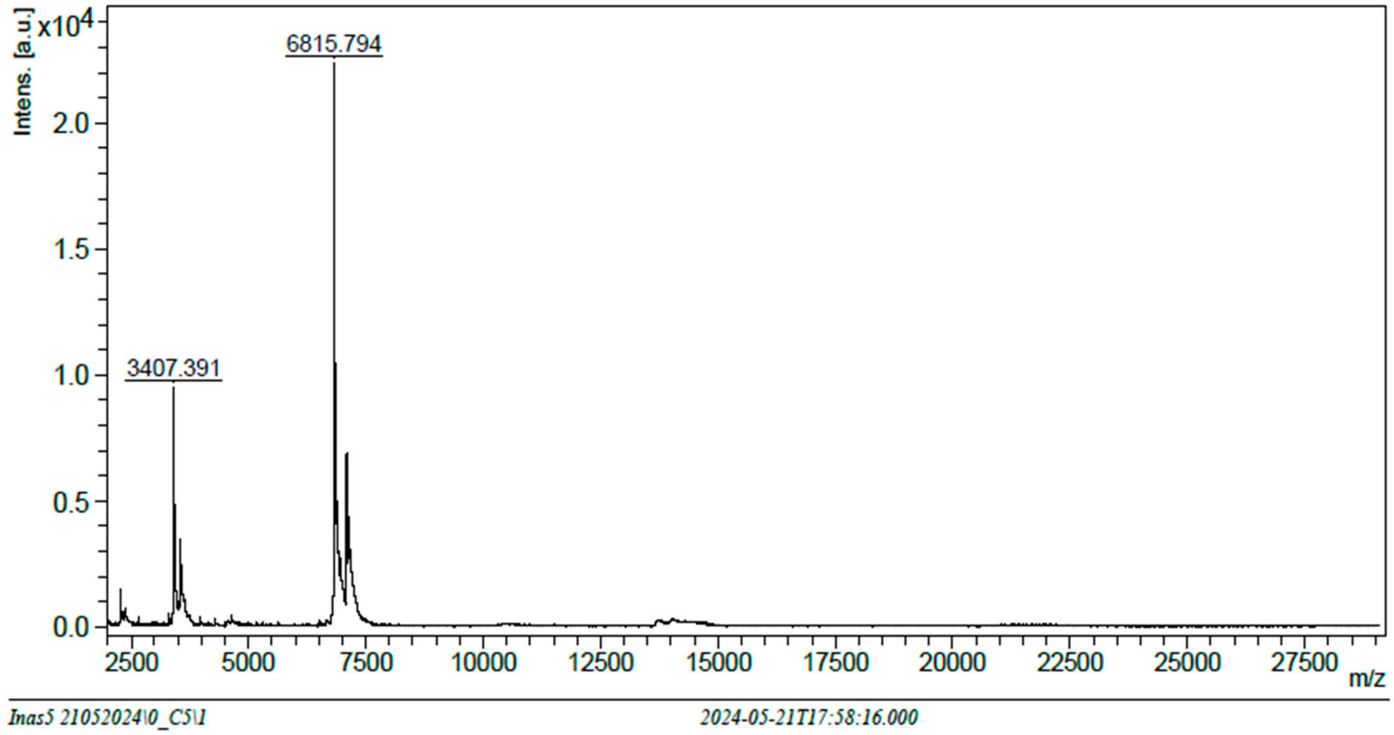


**Figure S9.** MALDI-TOF MS spectrum of siPCSK9_5-aS

| Name | Sequence (5′-3′) |
|---|---|
| siPSCK9_5-aS | rArGrArGrCrArGrArGrUrArArArGrGrUrGrGrCrUrC |
| Calculated Mw (H+ form) | 6838.21 |
| Founded Mw | 6815.8 |

siPSCK9_5

| m/z | Rel. Intens. | Area | SN |
|---|---|---|---|
| 2160.843 | 9 | 3835 | 9.8 |
| 3239.025 | 6 | 30096 | 20.5 |
| 6491.383 | 100 | 96244 | 159.3 |
| 6605.977 | 22 | 24292 | 35.0 |
| 6767.318 | 10 | 10671 | 17.0 |
| 13027.192 | 1 | 677 | 2.2 |

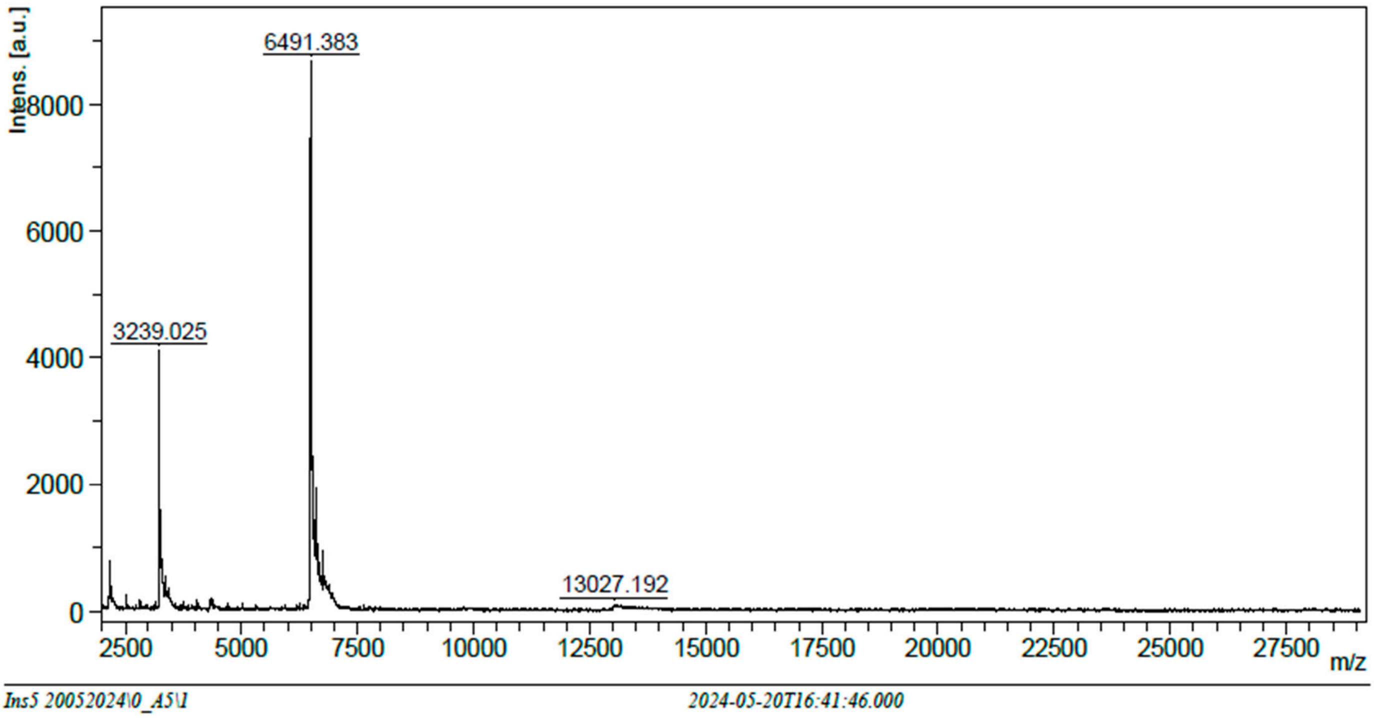


**Figure S10.** MALDI-TOF MS spectrum of siPCSK9_5-S.

| Name | Sequence (5′-3′) |
|---|---|
| siPSCK9_5-S | rGrCrCrArCrCrUrUrUrArCrUrCrUrGrCrUrCrUrArU |
| Calculated Mw (H+ form) | 6506.89 |
| Founded Mw | 6491.4 |

siPSCK9_6

| m/z | Rel. Intens. | Area | SN |
|---|---|---|---|
| 2213.564 | 6 | 26751 | 12.4 |
| 3320.134 | 11 | 85075 | 42.3 |
| 3458.026 | 3 | 14618 | 10.3 |
| 6653.946 | 100 | 240283 | 182.2 |
| 6930.208 | 26 | 59399 | 49.3 |

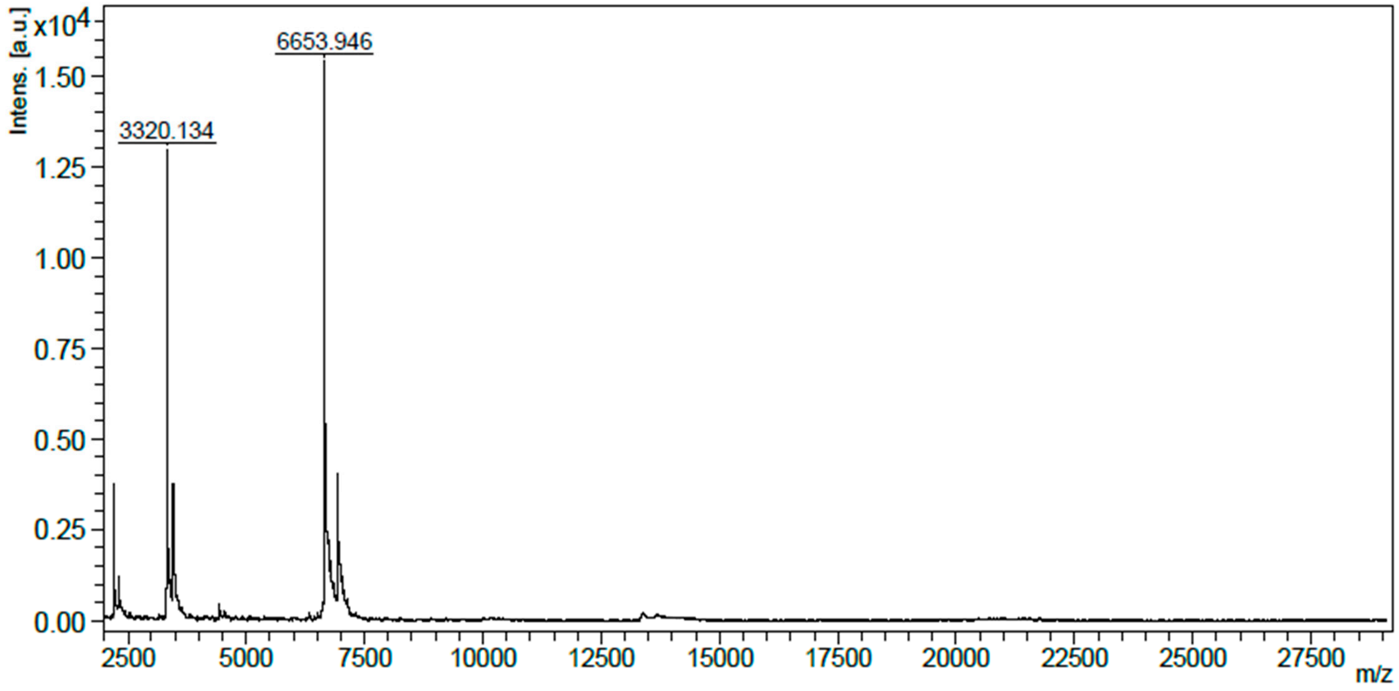


*Inas6 21052024\0_C6\1* *2024-05-21T17:58:31.000*

**Figure S11.** MALDI-TOF MS spectrum of siPCSK9_6-aS

| Name | Sequence (5′-3′) |
|---|---|
| siPSCK9_6-aS | rUrArArArUrGrUrCrUrGrCrUrUrGrCrUrUrGrGrGrU |
| Calculated Mw (H+ form) | 6667.97 |
| Founded Mw | 6654.0 |

siPSCK9_6

| m/z | Rel. Intens. | Area | SN |
|---|---|---|---|
| 2214.719 | 10 | 14732 | 9.1 |
| 2260.708 | 12 | 18620 | 10.6 |
| 2589.252 | 11 | 15192 | 10.1 |
| 2933.392 | 6 | 10318 | 6.6 |
| 3219.835 | 8 | 11336 | 8.1 |
| 3322.220 | 49 | 87135 | 52.8 |
| 3566.405 | 8 | 17605 | 9.1 |
| 6647.976 | 100 | 311111 | 180.3 |
| 6763.016 | 18 | 65847 | 33.7 |
| 6923.740 | 10 | 17499 | 17.7 |
| 13360.629 | 2 | 970 | 4.6 |

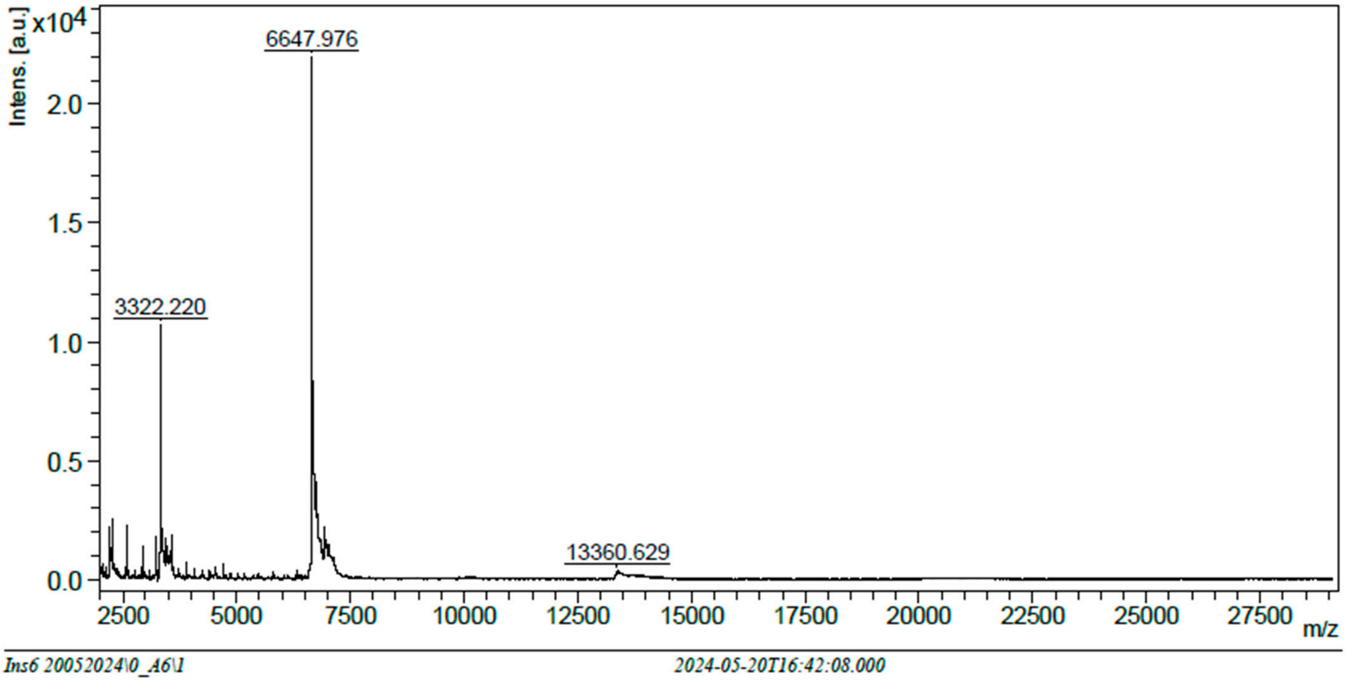


**Figure S12.** MALDI-TOF MS spectrum of siPCSK9_6-S.

| Name | Sequence (5′-3′) |
|---|---|
| siPSCK9_6-S | rCrCrArArGrCrArArGrCrArGrArCrArUrUrUrArUrC |
| Calculated Mw (H+ form) | 6663.1 |
| Founded Mw | 6648.0 |

siPSCK9_7

| m/z | Rel. Intens. | Area | SN |
|---|---|---|---|
| 3304.920 | 67 | 73451 | 48.4 |
| 3442.370 | 22 | 25951 | 16.5 |
| 6616.105 | 100 | 216266 | 124.7 |
| 6892.045 | 30 | 68032 | 38.0 |

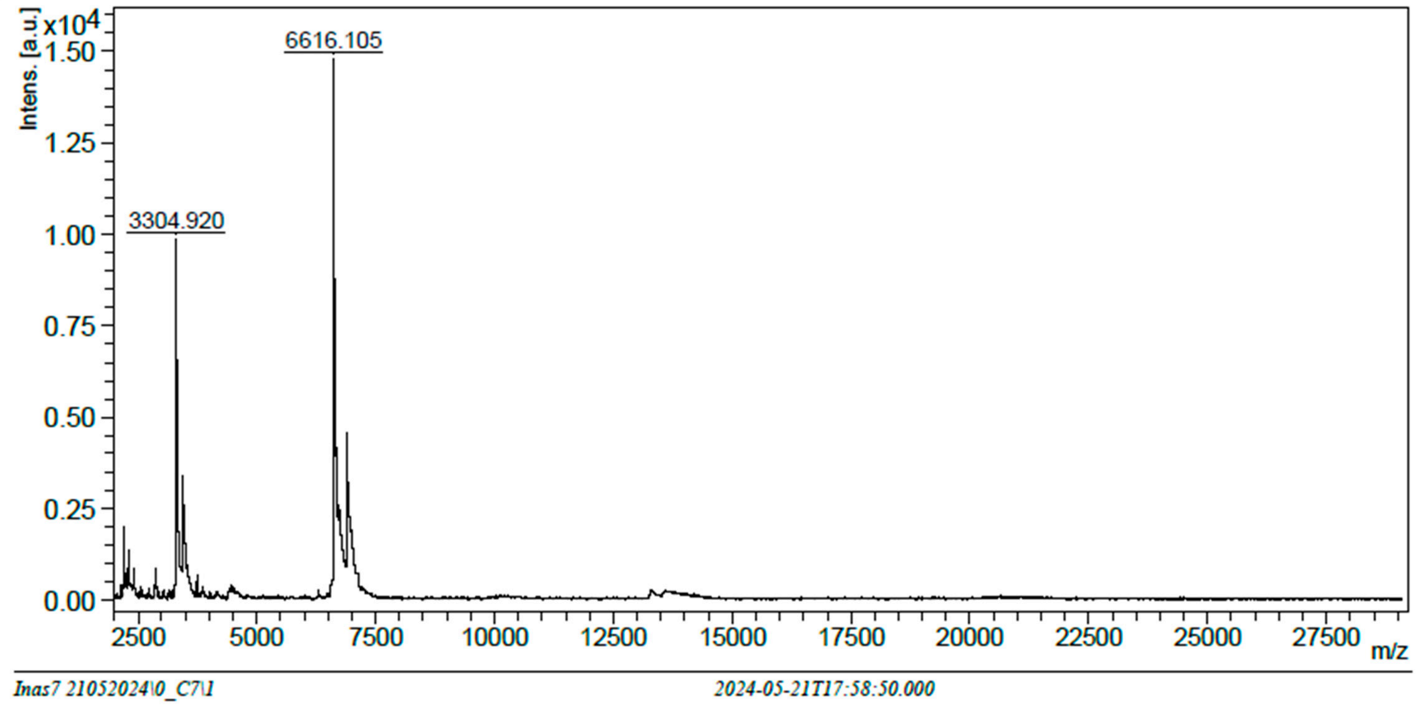


**Figure S13.** MALDI-TOF MS spectrum of siPCSK9_7-aS

| Name | Sequence (5′-3′) |
|---|---|
| siPSCK9_7-aS | rUrUrCrArGrArGrCrCrArGrCrCrCrArArUrCrUrGrC |
| Calculated Mw (H+ form) | 6631.05 |
| Founded Mw | 6616.1 |

siPSCK9_7

| m/z | Rel. Intens. | Area | SN |
|---|---|---|---|
| 2067.768 | 8 | 6063 | 5.8 |
| 2247.893 | 7 | 6670 | 5.1 |
| 2966.152 | 5 | 6890 | 4.6 |
| 3370.921 | 5 | 53773 | 18.0 |
| 3391.407 | 2 | 28611 | 6.3 |
| 3616.085 | 6 | 11751 | 6.1 |
| 3960.859 | 5 | 6139 | 5.0 |
| 6755.268 | 100 | 287852 | 157.7 |
| 6943.133 | 18 | 55066 | 28.7 |
| 7033.306 | 15 | 47033 | 24.1 |
| 13585.682 | 2 | 1400 | 6.3 |

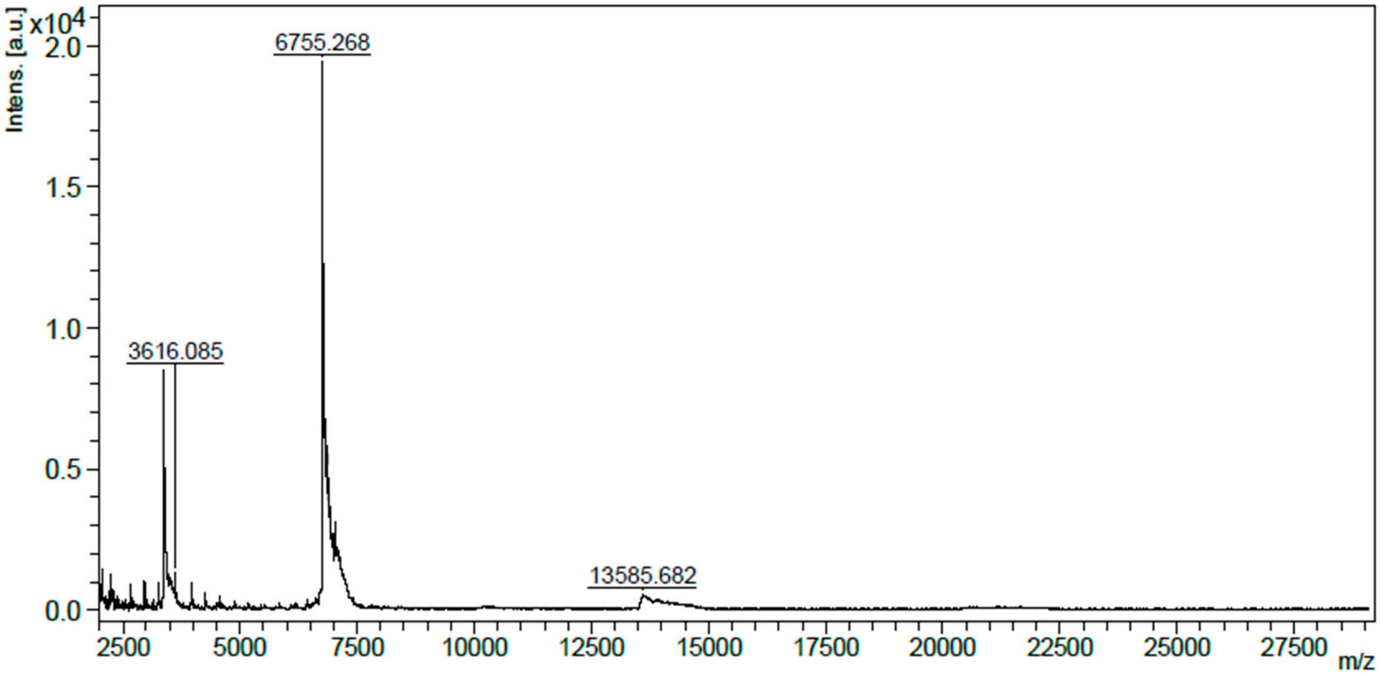


Ins7 20052024\0_A7\1 2024-05-20T16:42:42.000

**Figure S14.** MALDI-TOF MS spectrum of siPCSK9_7-S.

| Name | Sequence (5′-3′) |
|---|---|
| siPSCK9_7-S | rArGrArUrUrGrGrGrCrUrGrGrCrUrCrUrGrArArGrC |
| Calculated Mw (H+ form) | 6768.11 |
| Founded Mw | 6755.3 |

siPSCK9_8

| m/z | Rel. Intens. | Area | SN |
|---|---|---|---|
| 2201.112 | 10 | 10649 | 7.9 |
| 3301.994 | 49 | 67087 | 43.4 |
| 3439.392 | 14 | 19158 | 12.3 |
| 6609.435 | 100 | 263472 | 132.8 |
| 6885.606 | 26 | 71135 | 34.7 |

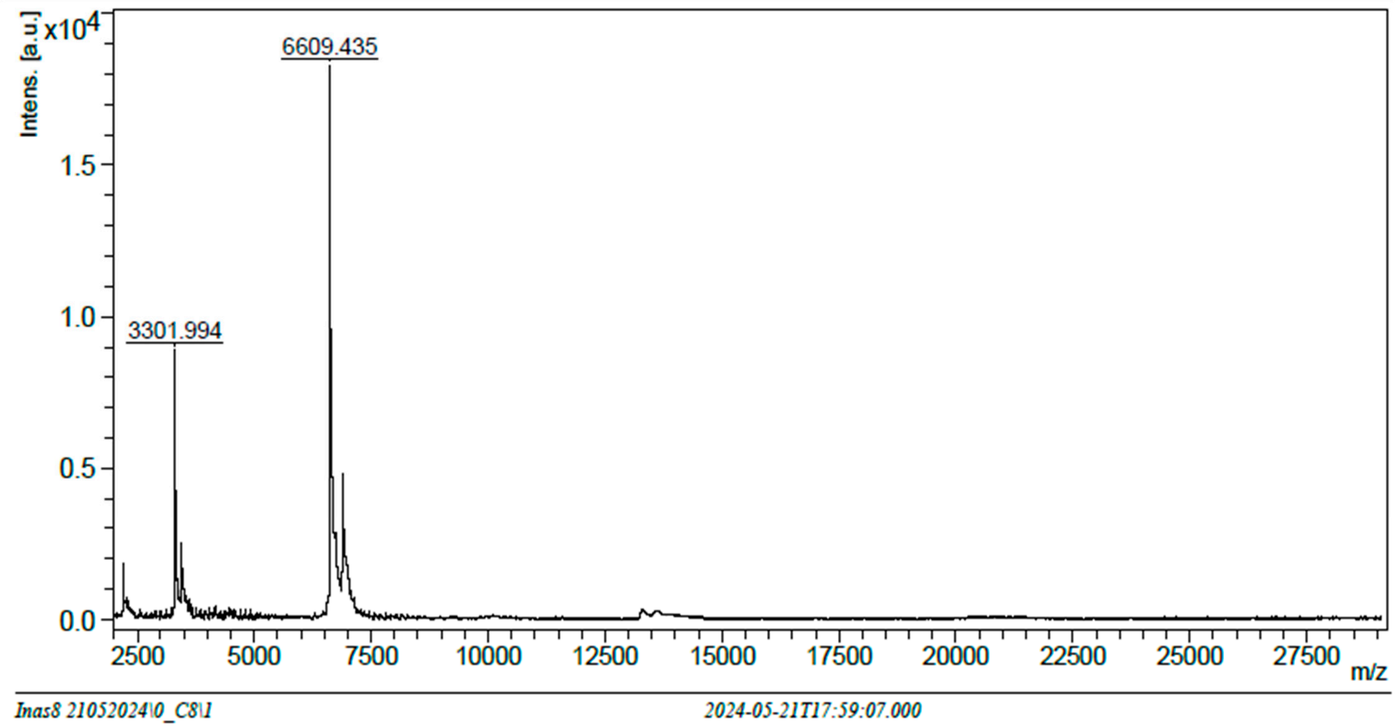


**Figure S15.** MALDI-TOF MS spectrum of siPCSK9_8-aS

| Name | Sequence (5′-3′) |
|---|---|
| siPSCK9_8-aS | rArGrCrCrCrArArUrCrUrGrCrGrUrUrUrCrUrGrGrC |
| Calculated Mw (H+ form) | 6624.99 |
| Founded Mw | 6609.4 |

siPSCK9_8

| m/z | Rel. Intens. | Area | SN |
|---|---|---|---|
| 2266.417 | 11 | 24086 | 6.0 |
| 2912.053 | 5 | 37321 | 6.4 |
| 3393.553 | 6 | 75455 | 12.9 |
| 3534.058 | 9 | 13625 | 5.4 |
| 3586.686 | 9 | 4906 | 5.4 |
| 3881.105 | 14 | 27465 | 9.1 |
| 4546.859 | 7 | 15955 | 4.8 |
| 4891.587 | 5 | 9952 | 3.5 |
| 6800.468 | 100 | 277811 | 105.3 |
| 6874.229 | 42 | 132999 | 44.6 |
| 7077.350 | 19 | 68011 | 20.4 |
| 13662.924 | 2 | 1576 | 3.1 |

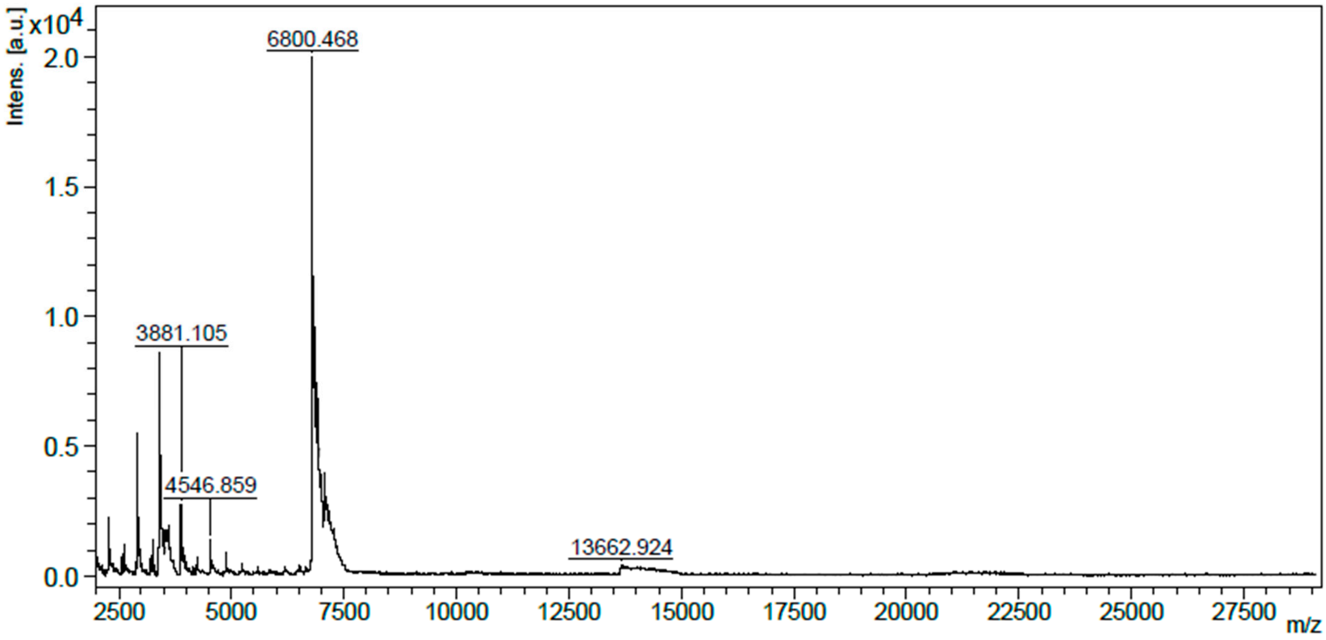


Ins8 20052024\0_A8\1 2024-05-20T16:43:04.000

**Figure S16.** MALDI-TOF MS spectrum of siPCSK9_8-S.

| Name | Sequence (5′-3′) |
|---|---|
| siPSCK9_8-S | rCrArGrArArArCrGrCrArGrArUrUrGrGrGrCrUrGrG |
| Calculated Mw (H+ form) | 6814.19 |
| Founded Mw | 6800.5 |

siPSCK9_9

| m/z | Rel. Intens. | Area | SN |
|---|---|---|---|
| 2210.651 | 18 | 11595 | 5.3 |
| 2324.835 | 26 | 21446 | 8.0 |
| 3313.415 | 8 | 45425 | 8.0 |
| 6641.702 | 100 | 171895 | 60.2 |
| 6917.413 | 25 | 46610 | 15.0 |

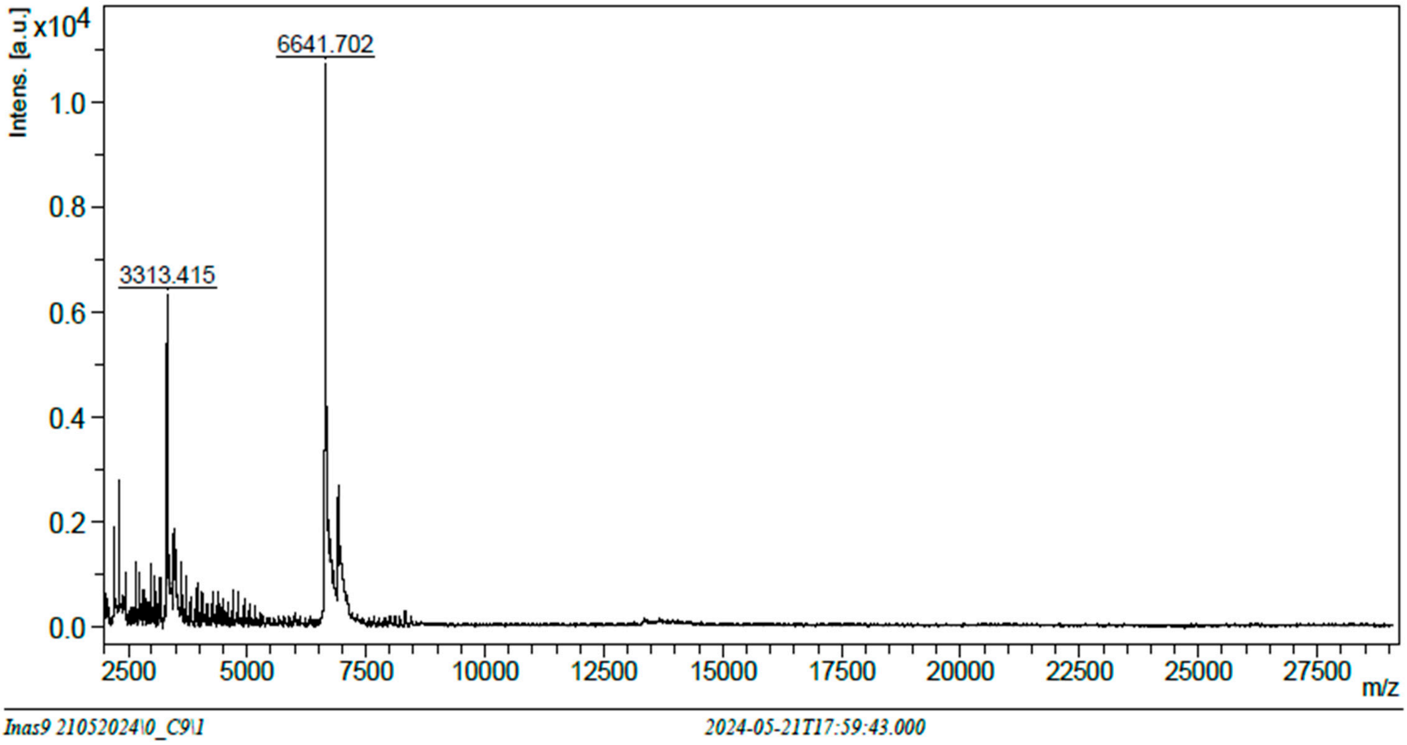


**Figure S17.** MALDI-TOF MS spectrum of siPCSK9_9-aS

| Name | Sequence (5′-3′) |
|---|---|
| siPSCK9_9-aS | rArGrUrGrCrArUrGrCrArCrCrArCrUrGrCrArCrArC |
| Calculated Mw (H+ form) | 6654.09 |
| Founded Mw | 6641.7 |

siPSCK9_9

| m/z | Rel. Intens. | Area | SN |
|---|---|---|---|
| 2239.605 | 12 | 6839 | 11.0 |
| 2635.947 | 6 | 4109 | 5.6 |
| 2980.373 | 6 | 4514 | 5.9 |
| 3285.047 | 7 | 5353 | 7.0 |
| 3357.346 | 8 | 25922 | 33.0 |
| 3933.888 | 6 | 5038 | 6.4 |
| 4261.964 | 5 | 4395 | 5.7 |
| 4567.527 | 5 | 4537 | 5.4 |
| 4912.530 | 3 | 2749 | 3.9 |
| 6728.815 | 100 | 115189 | 149.4 |
| 6843.605 | 21 | 30988 | 30.9 |
| 7004.672 | 12 | 16952 | 18.5 |

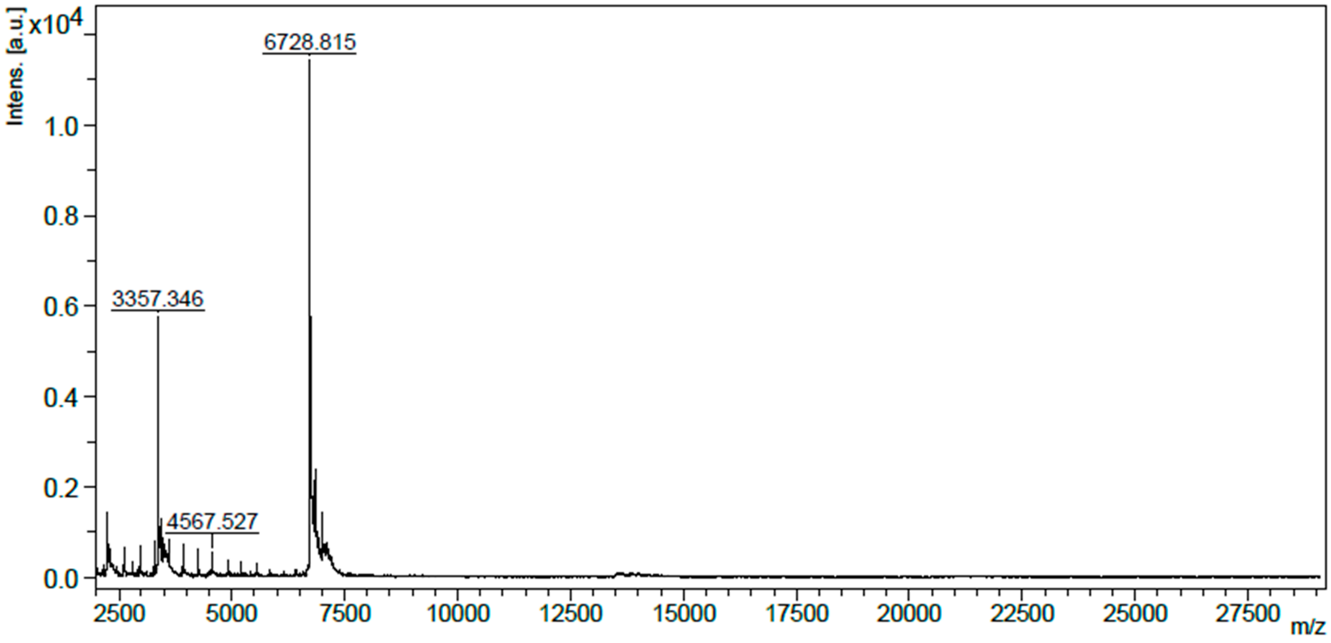


Ins9 20052024\0_A9\1 2024-05-20T16:43:33.000

**Figure S18.** MALDI-TOF MS spectrum of siPCSK9_9-S.

| Name | Sequence (5′-3′) |
|---|---|
| siPSCK9_9-S | rGrUrGrCrArGrUrGrGrUrGrCrArUrGrCrArCrUrGrU |
| Calculated Mw (H+ form) | 6745.07 |
| Founded Mw | 6728.8 |

siPSCK9_10

| m/z | Rel. Intens. | Area | SN |
|---|---|---|---|
| 2273.556 | 9 | 8104 | 6.7 |
| 3214.725 | 15 | 18904 | 12.0 |
| 3410.409 | 67 | 72936 | 56.4 |
| 3544.452 | 3 | 11510 | 9.3 |
| 6824.330 | 100 | 384268 | 131.3 |
| 7101.313 | 32 | 101407 | 42.8 |
| 13709.139 | 2 | 1771 | 6.0 |

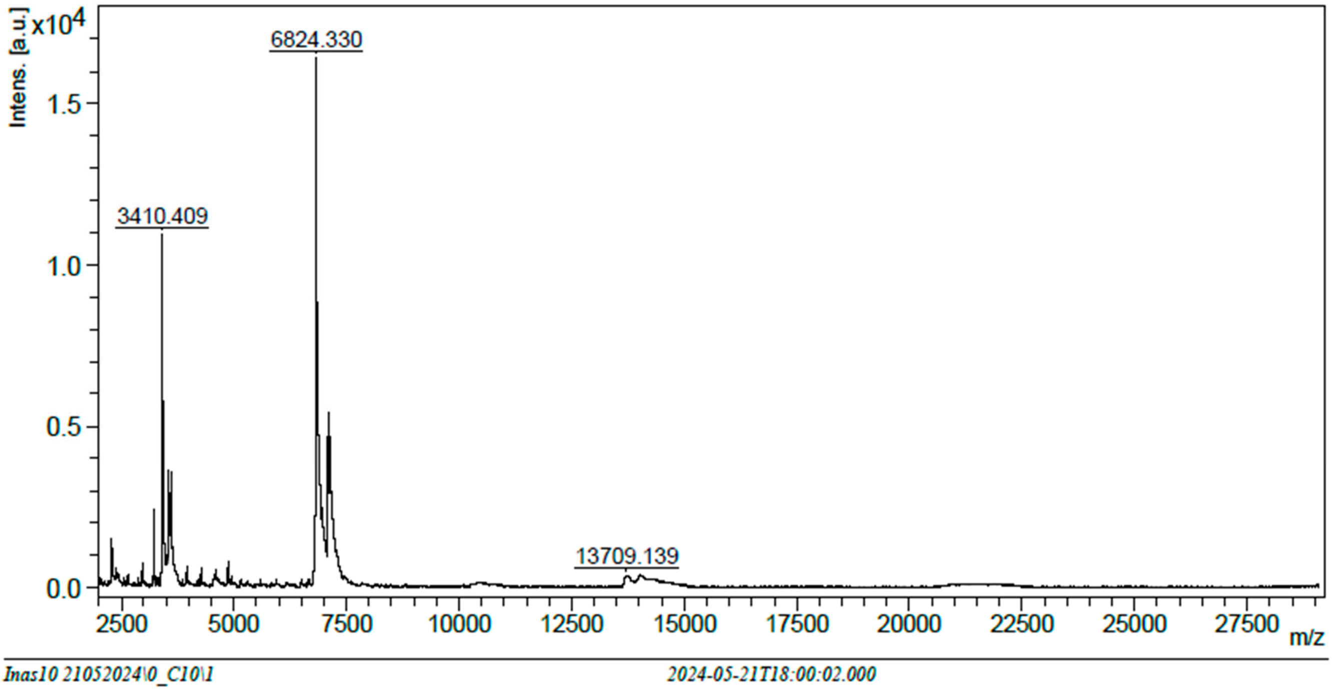


**Figure S19.** MALDI-TOF MS spectrum of siPCSK9_10-aS

| Name | Sequence (5′-3′) |
|---|---|
| siPSCK9_10-aS | rUrArGrArGrCrArGrArGrUrArArArGrGrUrGrGrCrU |
| Calculated Mw (H+ form) | 6839.2 |
| Founded Mw | 6824.3 |

siPSCK9_10

| m/z | Rel. Intens. | Area | SN |
|---|---|---|---|
| 2159.766 | 4 | 34656 | 7.7 |
| 2171.507 | 3 | 12829 | 8.7 |
| 3238.868 | 9 | 105655 | 41.5 |
| 3259.054 | 3 | 34125 | 13.4 |
| 6491.472 | 100 | 391254 | 202.9 |
| 6767.771 | 12 | 47810 | 26.0 |
| 13036.640 | 1 | 1313 | 4.8 |

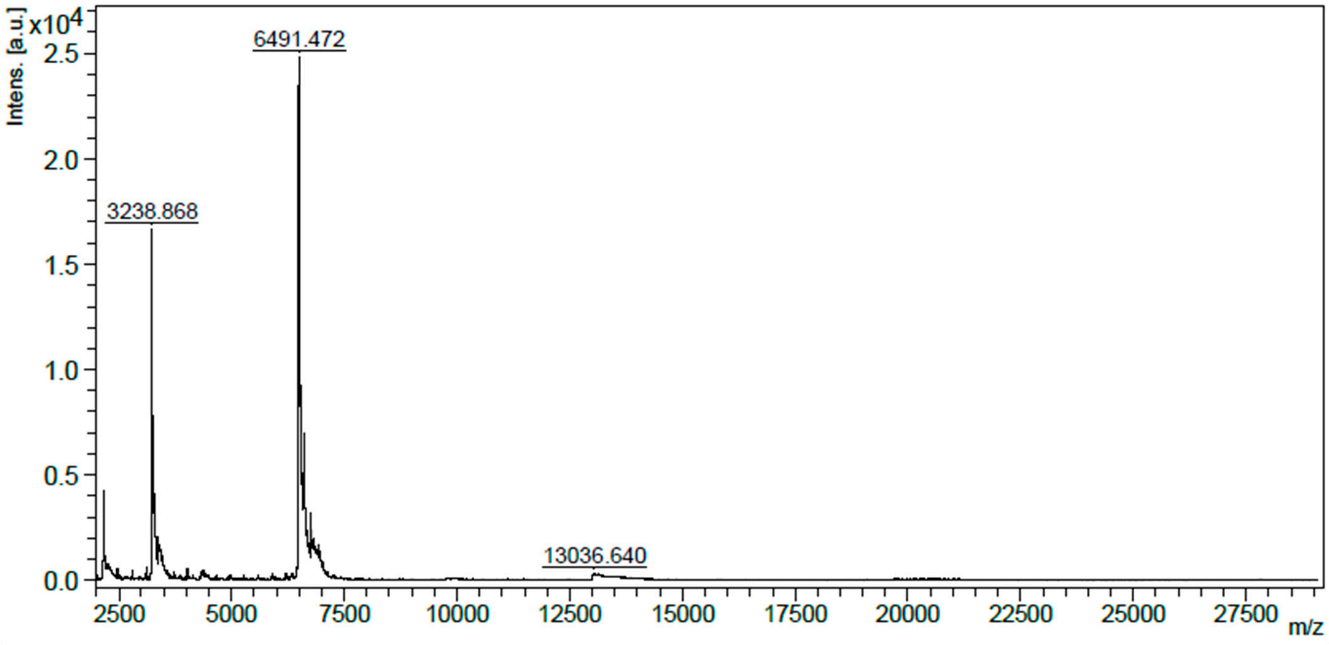


Ins10 20052024\0_A10\1 2024-05-20T16:43:53.000

**Figure S20.** MALDI-TOF MS spectrum of siPCSK9_10-S.

| Name | Sequence (5′-3′) |
|---|---|
| siPSCK9_10-S | rCrCrArCrCrUrUrUrArCrUrCrUrGrCrUrCrUrArUrG |
| Calculated Mw (H+ form) | 6506.89 |
| Founded Mw | 6491.5 |

siPCSK9_2_1

| m/z | Rel. Intens. | Area | SN |
|---|---|---|---|
| 3463.524 | 100 | 49804 | 16.0 |
| 3614.347 | 89 | 21264 | 19.3 |
| 6935.692 | 20 | 24739 | 7.4 |
| 7239.180 | 13 | 12947 | 6.7 |
| 14251.612 | 40 | 1113 | 3.7 |

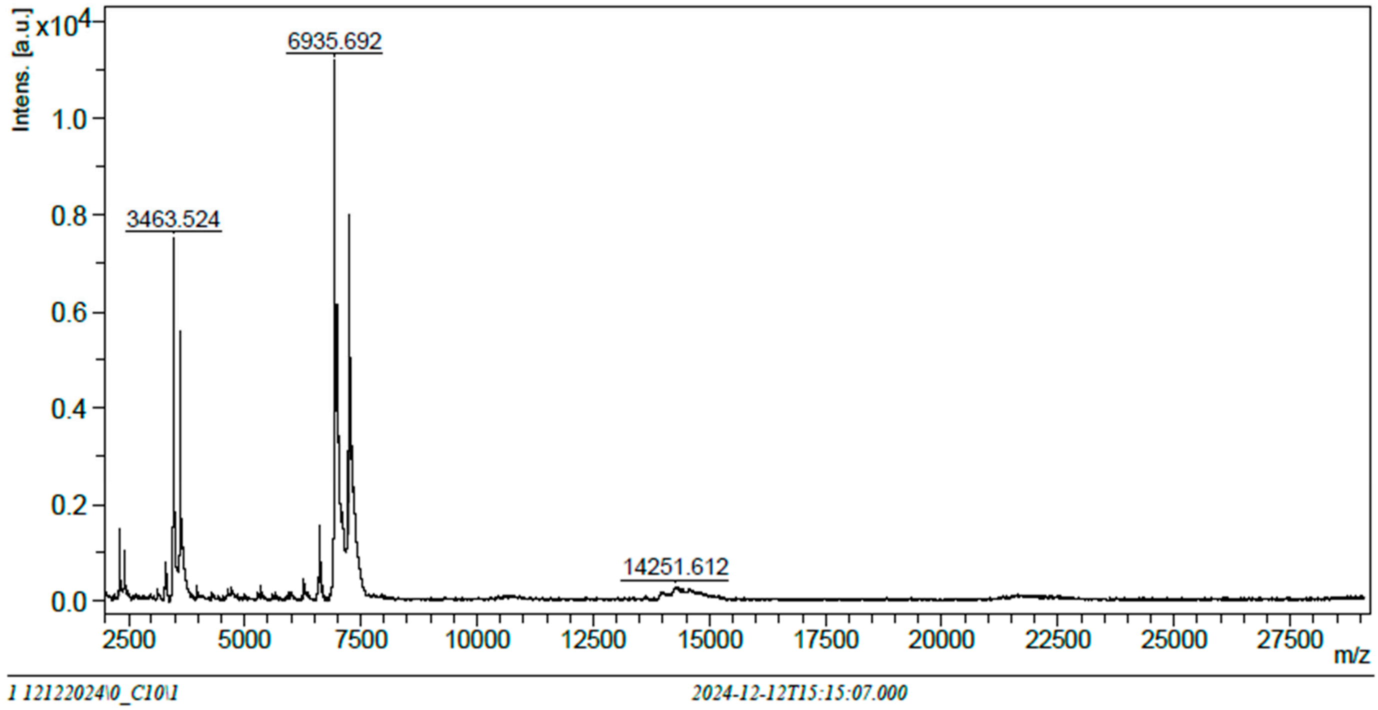


**Figure S21.** MALDI-TOF MS spectrum of siPCSK9_2_1-S

| Name | Sequence (5′-3′) |
|---|---|
| siPCSK9_2_1-S | mCmAmAmGmCmAmAmGmCmAmGmAmCmAmUmUmUmAmUmCmU |
| Calculated Mw (H+ form) | 6958,72 |
| Founded Mw | 6935,69 |

siPCSK9-2-1

| m/z | Rel. Intens. | Area | SN |
|---|---|---|---|
| 2319.460 | 13 | 4182 | 7.3 |
| 3320.523 | 9 | 3708 | 6.2 |
| 3480.563 | 55 | 51362 | 36.5 |
| 3631.891 | 16 | 13825 | 10.9 |
| 6646.399 | 21 | 29874 | 21.7 |
| 6967.016 | 100 | 163168 | 104.2 |
| 7270.670 | 27 | 44024 | 28.1 |
| 14016.564 | 2 | 800 | 4.0 |

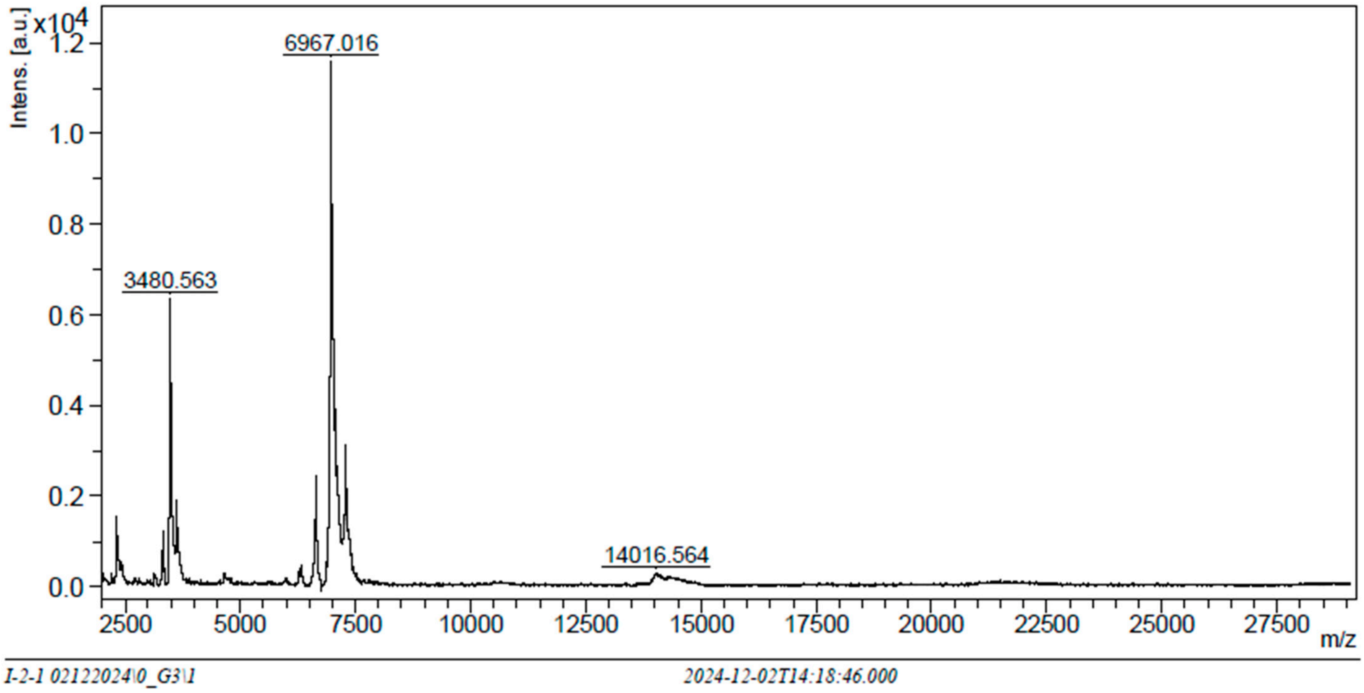


**Figure S22.** MALDI-TOF MS spectrum of siPCSK9_2_1-aS

| Name | Sequence (5′-3′) |
|---|---|
| siPCSK9_2_1-aS | mAmUmAmAmAmUmGmUmCmUmGmCmUmUmGmCmUmUmGmGmG |
| Calculated Mw (H+ form) | 6985,66 |
| Founded Mw | 6967,02 |

siPCSK9_2_2

| m/z | Rel. Intens. | Area | SN |
|---|---|---|---|
| 2299.623 | 5 | 4191 | 7.3 |
| 2535.732 | 6 | 5854 | 8.8 |
| 3446.782 | 7 | 24564 | 39.7 |
| 3597.415 | 4 | 13319 | 23.1 |
| 6587.369 | 10 | 14497 | 18.9 |
| 6907.874 | 100 | 165657 | 188.0 |
| 7205.019 | 1 | 8193 | 55.4 |
| 14170.488 | 1 | 434 | 4.2 |

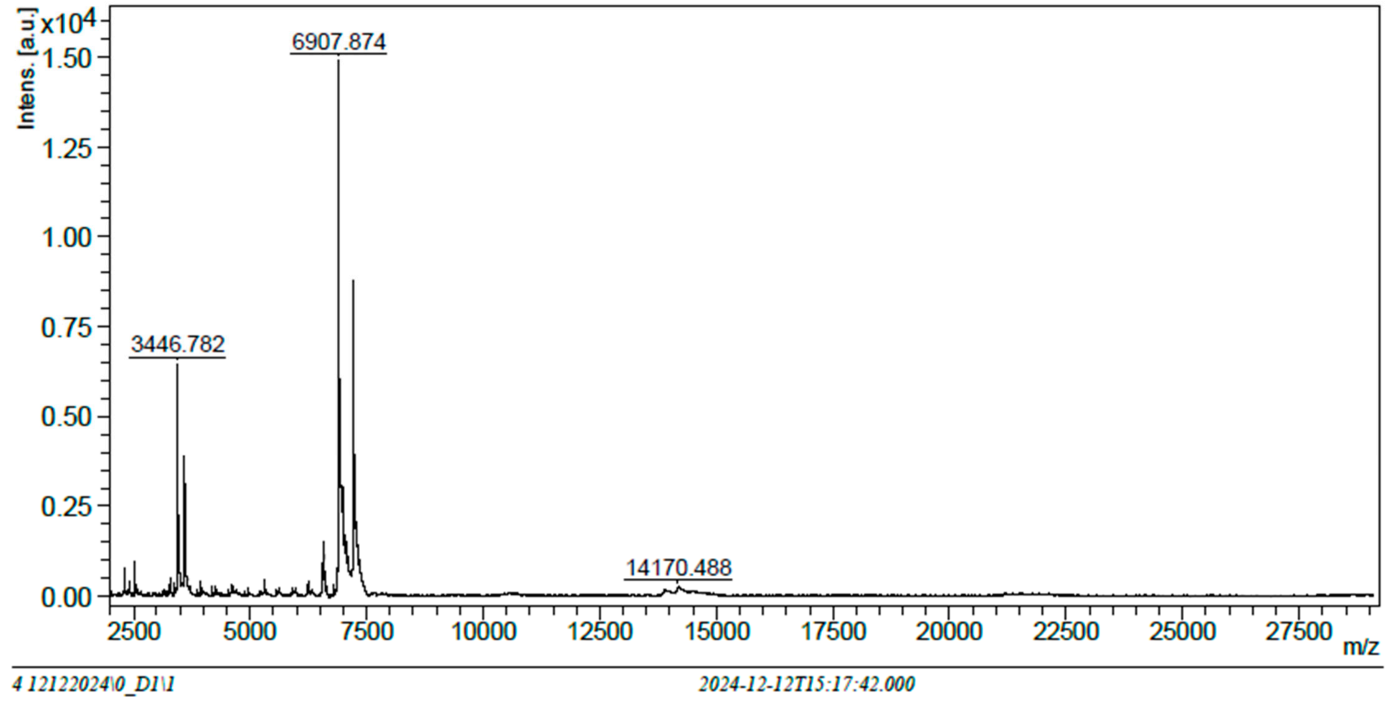


**Figure S23.** MALDI-TOF MS spectrum of siPCSK9_2_2-S

| Name | Sequence (5′-3′) |
|---|---|
| siPCSK9_2_2-S | mCmAmAmGmCmAmAmGmCmAfGfAfCmAmUmUmUmAmUmCmU |
| Calculated Mw (H+ form) | 6922,6 |
| Founded Mw | 6907,87 |

siPCSK9-2-2

| m/z | Rel. Intens. | Area | SN |
|---|---|---|---|
| 2320.300 | 12 | 16932 | 11.3 |
| 3321.162 | 11 | 12268 | 12.0 |
| 3480.744 | 66 | 84135 | 76.5 |
| 6648.029 | 21 | 51457 | 36.5 |
| 6969.716 | 100 | 375833 | 175.3 |
| 7274.218 | 35 | 122115 | 61.6 |
| 13981.752 | 2 | 2598 | 5.0 |

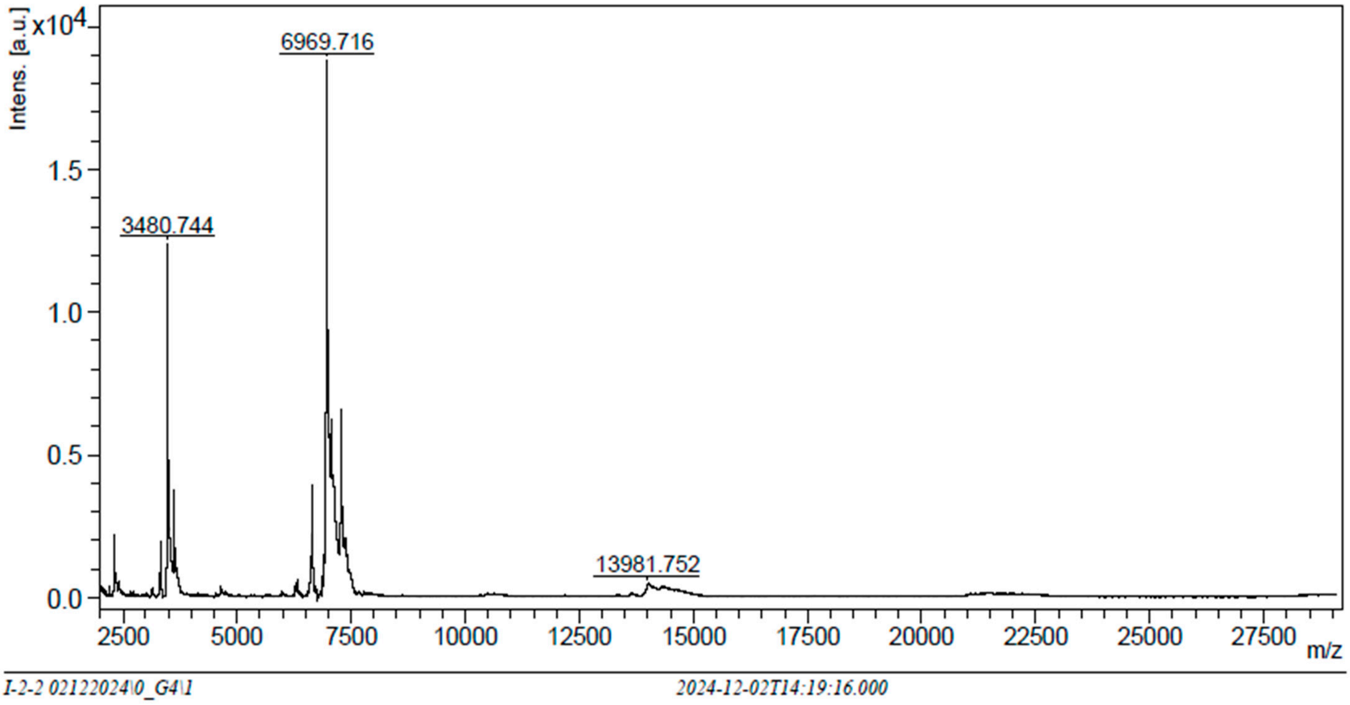


**Figure S24.** MALDI-TOF MS spectrum of siPCSK9_2_2-aS

| Name | Sequence (5′-3′) |
|---|---|
| siPCSK9_2_2-aS | mAmUmAmAmAmUmGmUmCmUmGmCmUmUmGmCmUmUmGmGmG |
| Calculated Mw (H+ form) | 6985,66 |
| Founded Mw | 6969,72 |

siPCSK9_2_3

| m/z | Rel. Intens. | Area | SN |
|---|---|---|---|
| 2298.862 | 17 | 8634 | 12.8 |
| 3445.519 | 11 | 24156 | 46.1 |
| 3596.232 | 7 | 14703 | 30.8 |
| 6586.023 | 10 | 9729 | 12.2 |
| 6906.243 | 100 | 112909 | 118.6 |
| 7202.964 | 1 | 7818 | 21.8 |

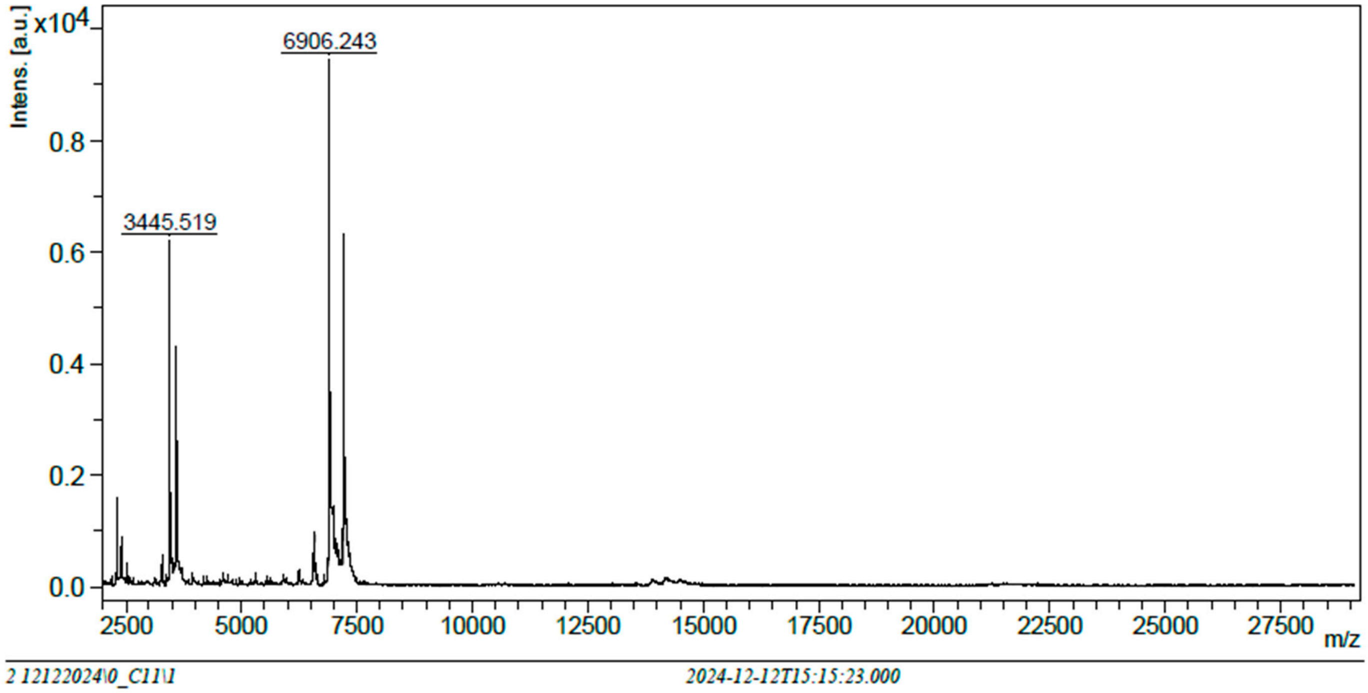


**Figure S25.** MALDI-TOF MS spectrum of siPCSK9_2_3-S

| Name | Sequence (5′-3′) |
|---|---|
| siPCSK9_2_3-S | mCmAmAmGmCmAmAmGmCmAfGfAfCmAmUmUmUmAmUmCmU |
| Calculated Mw (H+ form) | 6922,6 |
| Founded Mw | 6906,24 |

siPCSK9-2-3

| m/z | Rel. Intens. | Area | SN |
|---|---|---|---|
| 2284.505 | 6 | 3289 | 4.6 |
| 3425.292 | 4 | 50655 | 7.4 |
| 6552.064 | 13 | 24351 | 12.4 |
| 6861.076 | 100 | 263895 | 97.5 |
| 13786.753 | 3 | 3047 | 4.7 |

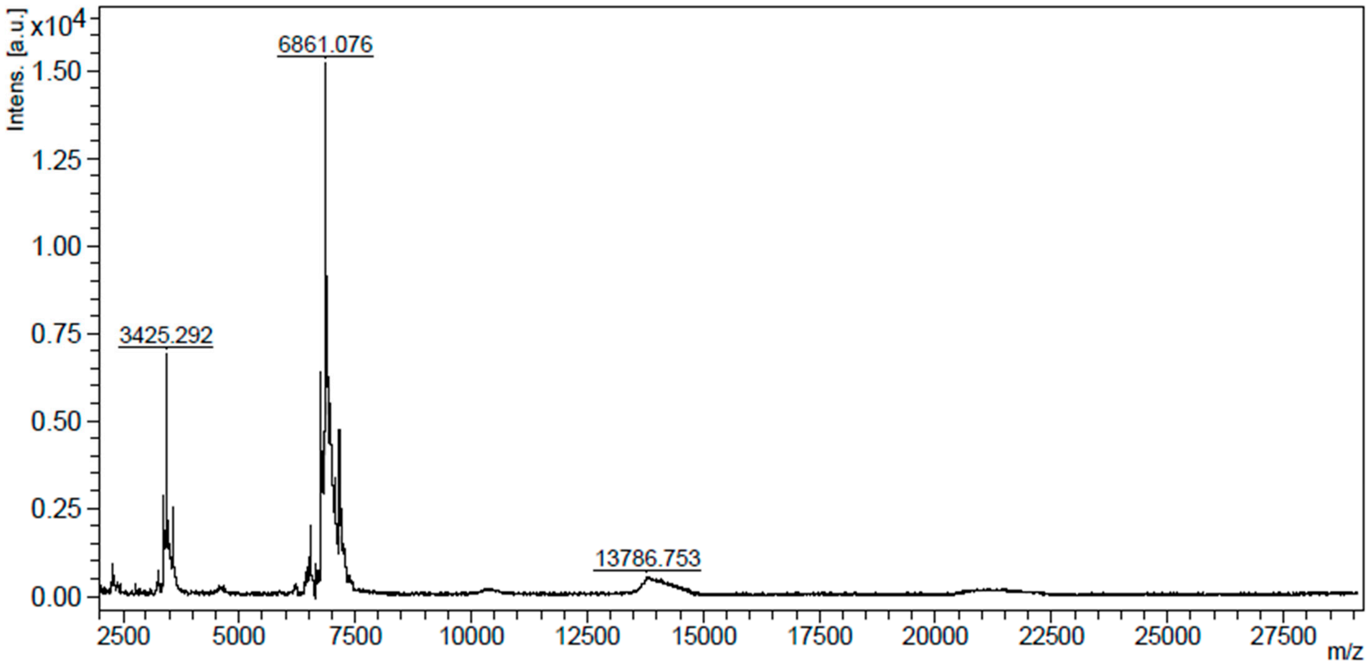


I-2-3 02122024\0_G5\1 2024-12-02T14:19:45.000

**Figure S26.** MALDI-TOF MS spectrum of siPCSK9_2_3-aS

| Name | Sequence (5′-3′) |
|---|---|
| siPCSK9_2_3-aS | mAfUmAfAmAfUmGfUmCmUmGfCmUfUmGfCmUfUmGfGmG |
| Calculated Mw (H+ form) | 6877,3 |
| Founded Mw | 6861,08 |

siPCSK9-2-4

| m/z | Rel. Intens. | Area | SN |
|---|---|---|---|
| 2534.512 | 7 | 2269 | 6.5 |
| 3447.080 | 4 | 26294 | 12.0 |
| 3597.546 | 3 | 17107 | 11.8 |
| 6587.233 | 10 | 9547 | 13.1 |
| 6907.882 | 100 | 116198 | 126.2 |
| 7204.840 | 1 | 6669 | 39.9 |

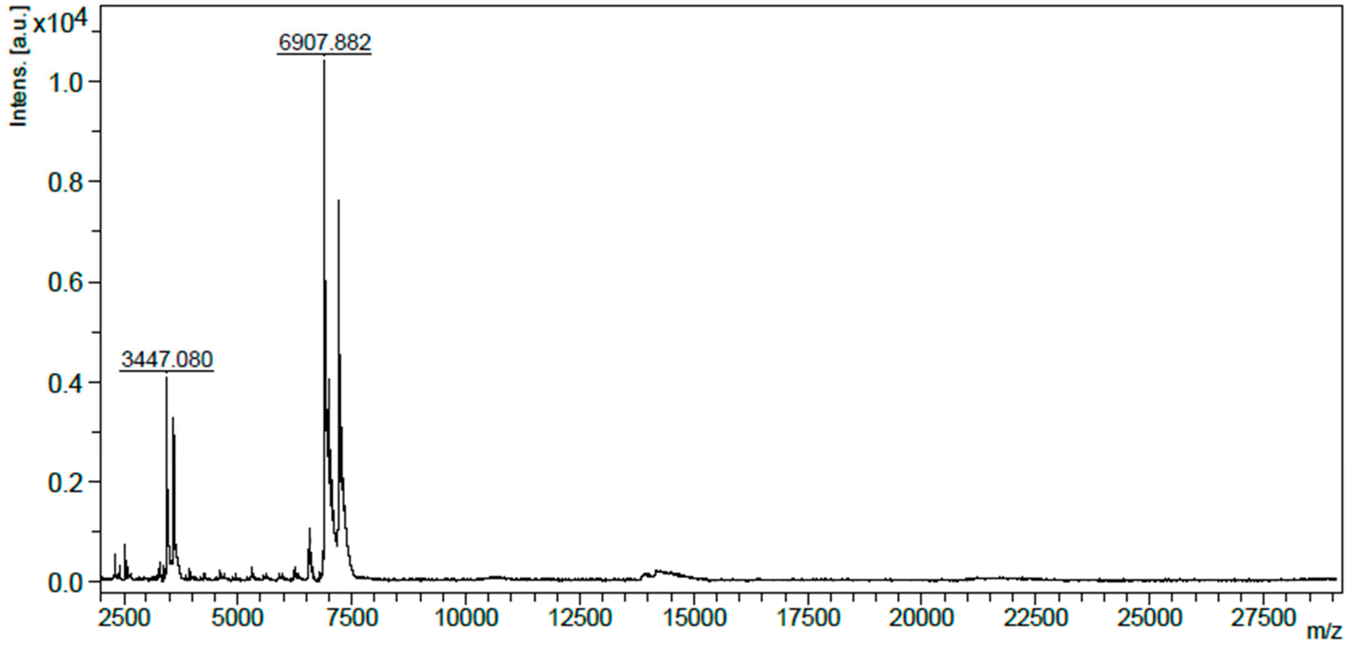


*3 12122024\0_C12\1* *2024-12-12T15:15:47.000*

**Figure S27.** MALDI-TOF MS spectrum of siPCSK9_2_4-S

| Name | Sequence (5′-3′) |
|---|---|
| siPCSK9_2_4-S | mCmAmAmGmCmAmAmGmCmAfGfAfCmAmUmUmUmAmUmCmU |
| Calculated Mw (H+ form) | 6922,6 |
| Founded Mw | 6907,88 |

siPCSK9-2-4

| m/z | Rel. Intens. | Area | SN |
|---|---|---|---|
| 3441.716 | 5 | 57249 | 17.7 |
| 6575.253 | 15 | 29897 | 26.7 |
| 6897.746 | 100 | 371509 | 178.6 |
| 7201.346 | 20 | 44227 | 36.8 |
| 13839.701 | 3 | 1573 | 8.6 |

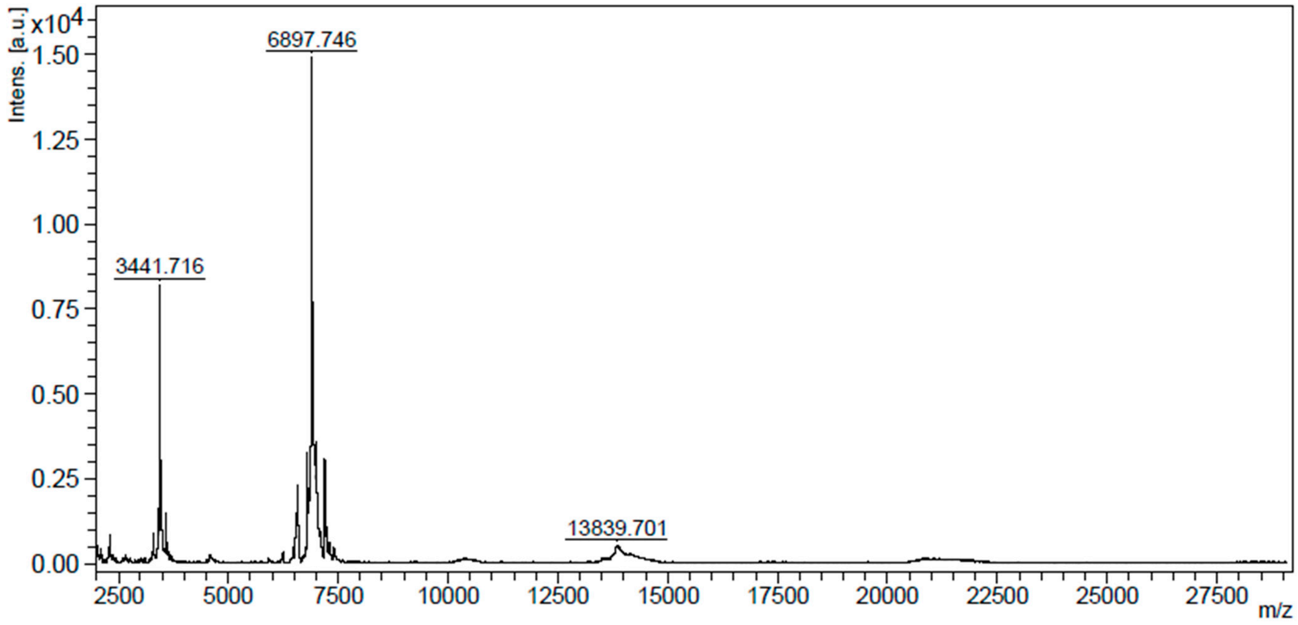


I-2-4 02122024\0_G6\1 2024-12-02T14:19:57.000

**Figure S28.** MALDI-TOF MS spectrum of siPCSK9_2_4-aS

| Name | Sequence (5′-3′) |
|---|---|
| siPCSK9_2_4-aS | mAfUfAfAmAmUmGmUmCmUmGmCmUmUmGmCmUfUfGfGmG |
| Calculated Mw (H+ form) | 6913,42 |
| Founded Mw | 6897,75 |

siPCSK9-2-5

| m/z | Rel. Intens. | Area | SN |
|---|---|---|---|
| 2299.222 | 17 | 7013 | 13.3 |
| 3446.104 | 8 | 31968 | 21.6 |
| 3596.511 | 6 | 18457 | 23.7 |
| 6587.511 | 7 | 4777 | 9.4 |
| 6907.851 | 100 | 75775 | 144.4 |
| 7205.322 | 1 | 5475 | 18.1 |

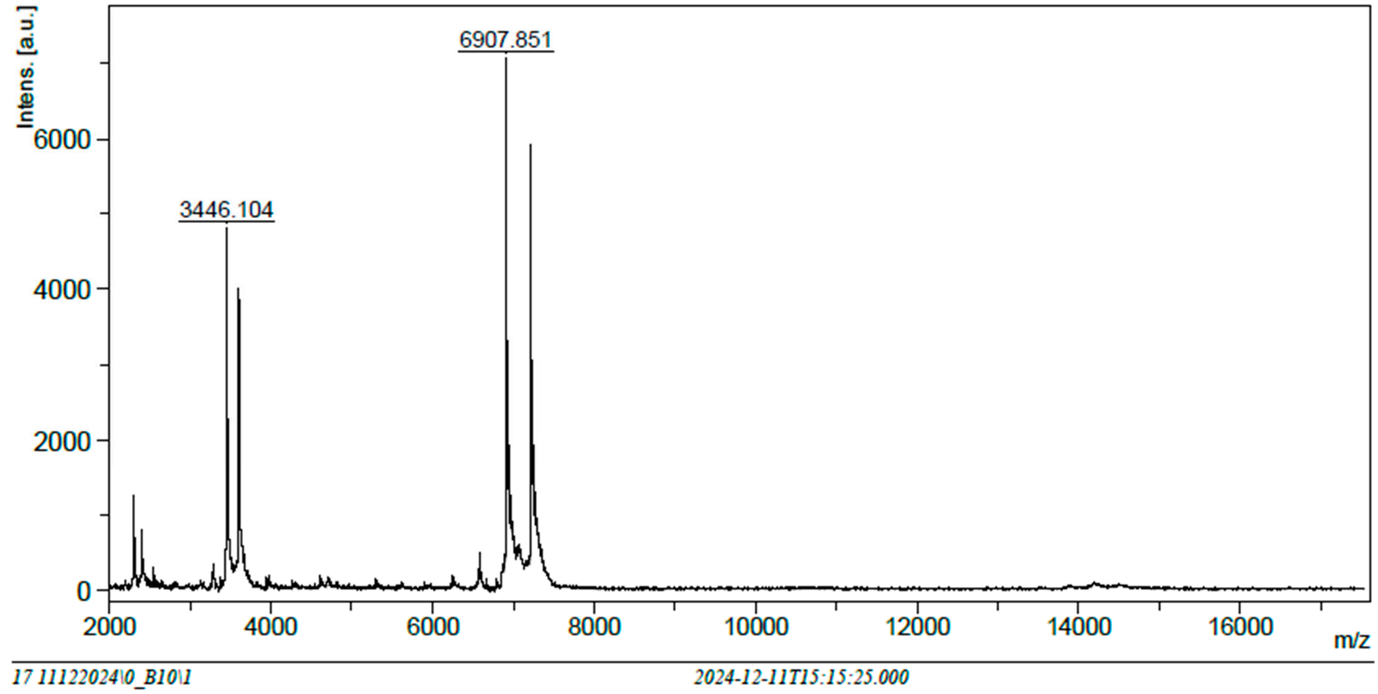


**Figure S29.** MALDI-TOF MS spectrum of siPCSK9_2_5-S

| Name | Sequence (5′-3′) |
|---|---|
| siPCSK9_2_5-S | mCmAmAmGmCmAmAmGmCmAfGfAfCmAmUmUmUmAmUmCmU |
| Calculated Mw (H+ form) | 6922,6 |
| Founded Mw | 6907,85 |

siPCSK9-2-5

| m/z | Rel. Intens. | Area | SN |
|---|---|---|---|
| 3430.909 | 5 | 33913 | 11.8 |
| 6564.198 | 11 | 17582 | 14.4 |
| 6778.389 | 29 | 48601 | 36.8 |
| 6873.779 | 100 | 223047 | 126.5 |
| 7177.370 | 19 | 28468 | 24.3 |
| 13805.111 | 4 | 1607 | 6.2 |

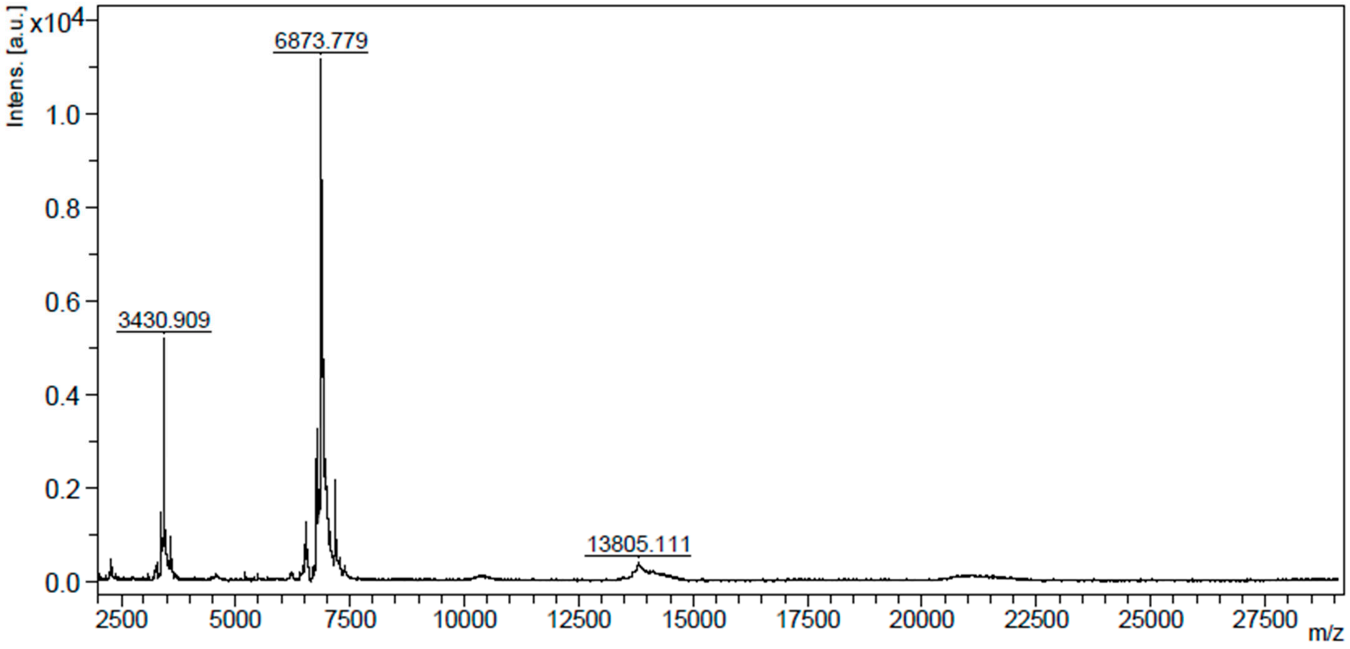


**Figure S30.** MALDI-TOF MS spectrum of siPCSK9_2_5-aS

| Name | Sequence (5′-3′) |
|---|---|
| siPCSK9_2_5-aS | mAfUmAfAfAfUmGmUmCmUmGmCmUmUmGfCfUfUmGfGmG |
| Calculated Mw (H+ form) | 6889,34 |
| Founded Mw | 6873,78 |

siPCSK9-2-6

| m/z | Rel. Intens. | Area | SN |
|---|---|---|---|
| 2299.249 | 11 | 7957 | 12.3 |
| 3446.606 | 8 | 30439 | 32.4 |
| 3597.252 | 6 | 21200 | 31.7 |
| 6586.901 | 7 | 10422 | 14.1 |
| 6907.758 | 100 | 178701 | 195.3 |
| 6939.446 | 0 | 4467 | 9.7 |
| 7204.656 | 1 | 14837 | 12.2 |

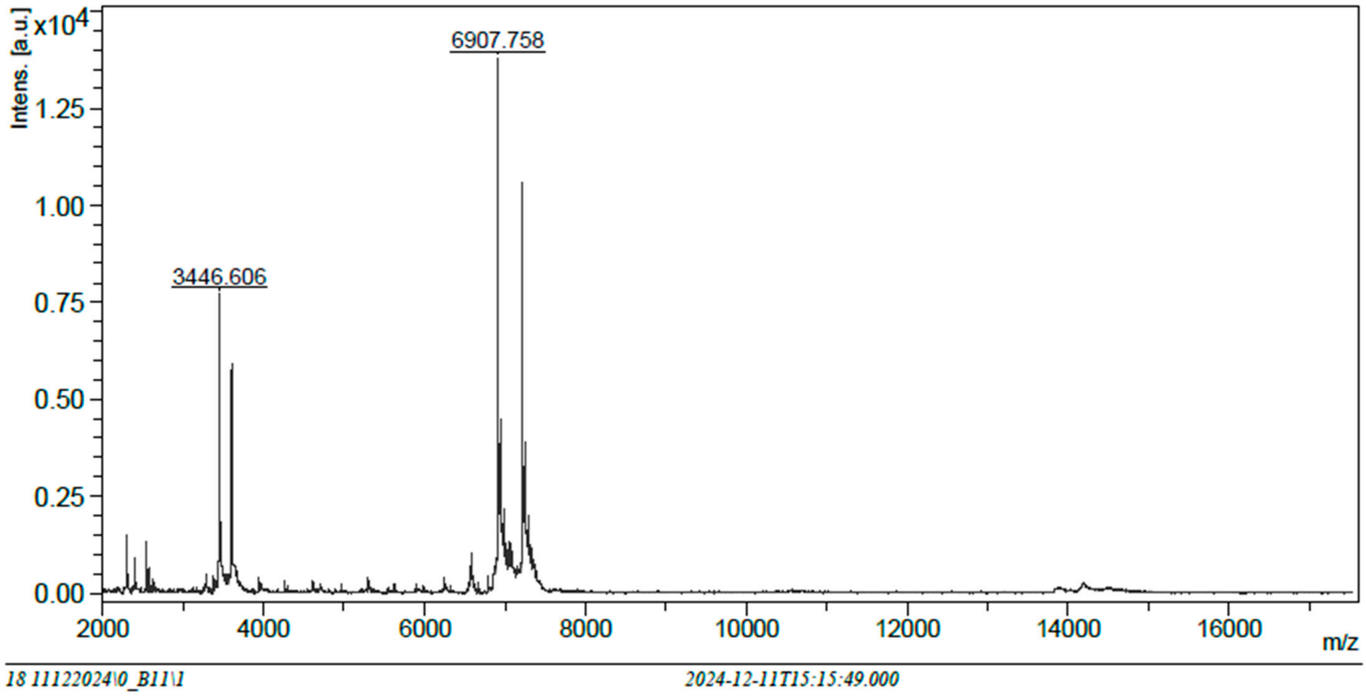


**Figure S31.** MALDI-TOF MS spectrum of siPCSK9_2_6-S

| Name | Sequence (5′-3′) |
|---|---|
| siPCSK9_2_6-S | mCmAmAmGmCmAmAmGmCmAfGfAfCmAmUmUmUmAmUmCmU |
| Calculated Mw (H+ form) | 6922,6 |
| Founded Mw | 6907,76 |

siPCSK9-2-6

| m/z | Rel. Intens. | Area | SN |
|---|---|---|---|
| 2278.699 | 8 | 9210 | 6.3 |
| 3442.852 | 5 | 63895 | 12.6 |
| 6575.895 | 12 | 24598 | 15.9 |
| 6801.950 | 37 | 96904 | 49.1 |
| 6897.000 | 100 | 359480 | 134.3 |
| 7200.582 | 36 | 101477 | 49.6 |
| 13877.577 | 2 | 1268 | 5.7 |

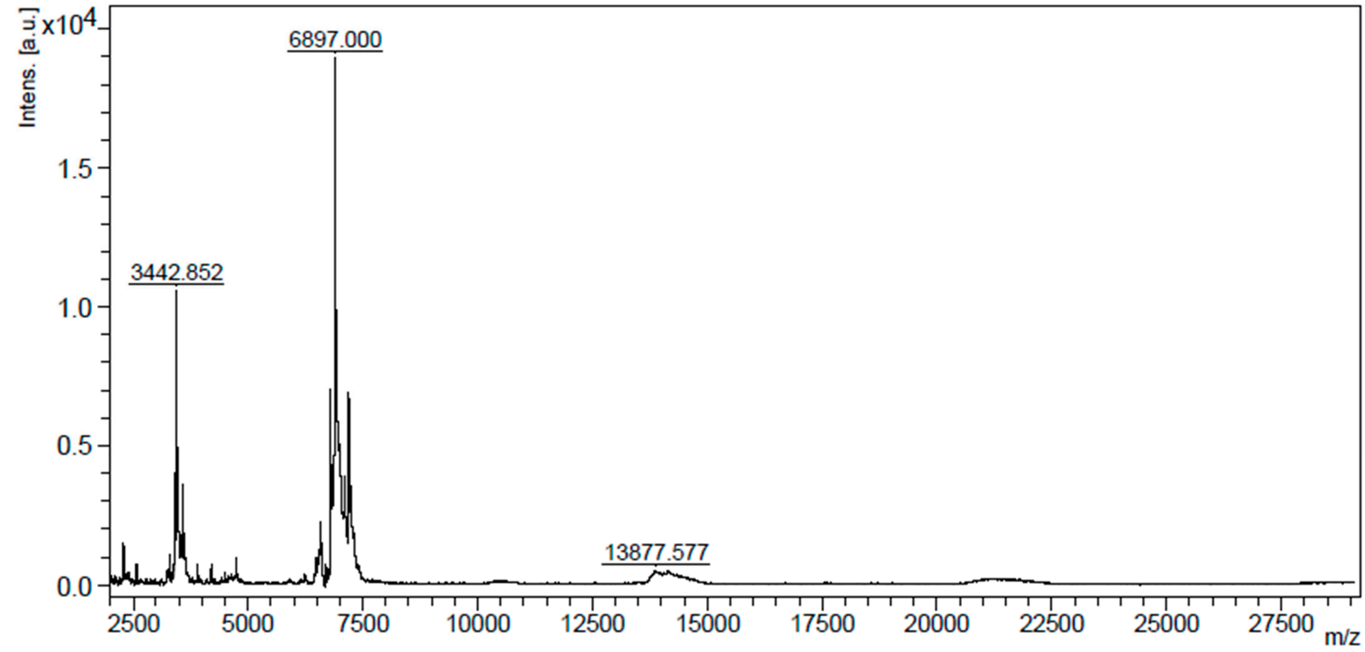


I-2-6 02122024\0_G8\1 2024-12-02T14:20:25.000

**Figure S32.** MALDI-TOF MS spectrum of siPCSK9_2_6-aS

| Name | Sequence (5′-3′) |
|---|---|
| siPCSK9_2_6-aS | mAmUmAmAmAfUfGfUmCmUmGfCfUfUmGmCmUmUmGmGmG |
| Calculated Mw (H+ form) | 6913,42 |
| Founded Mw | 6897,0 |

siPCSK9-2-7

| m/z | Rel. Intens. | Area | SN |
|---|---|---|---|
| 2299.020 | 7 | 27437 | 8.5 |
| 3448.207 | 10 | 53337 | 26.8 |
| 3467.817 | 2 | 6568 | 7.1 |
| 3598.805 | 6 | 31431 | 19.6 |
| 6589.236 | 7 | 8764 | 11.4 |
| 6910.102 | 100 | 198572 | 170.4 |
| 7207.134 | 1 | 7797 | 29.3 |

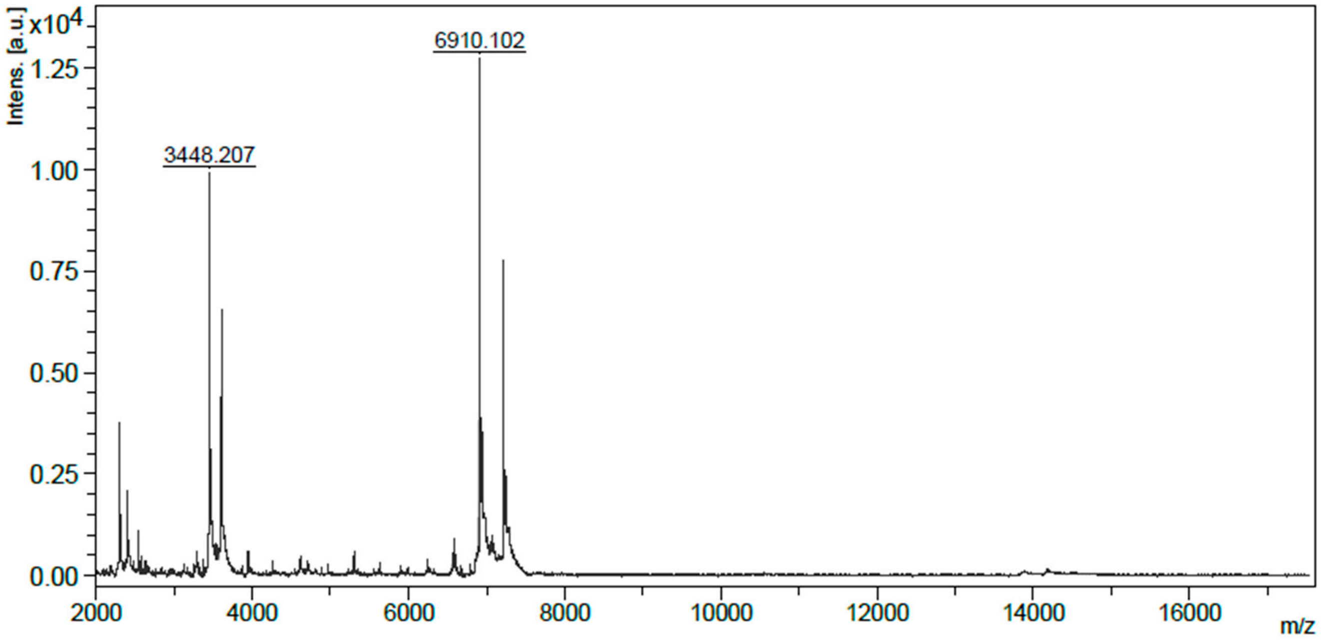


19 11122024\0_C1\1 2024-12-11T15:16:30.000

**Figure S33.** MALDI-TOF MS spectrum of siPCSK9_2_7-S

| Name | Sequence (5′-3′) |
|---|---|
| siPCSK9_2_7-S | mCmAmAmGmCmAmAmGmCmAfGfAfCmAmUmUmUmAmUmCmU |
| Calculated Mw (H+ form) | 6922,6 |
| Founded Mw | 6907,76 |

siPCSK9-2-7

| m/z | Rel. Intens. | Area | SN |
|---|---|---|---|
| 2160.597 | 21 | 31091 | 15.0 |
| 3443.431 | 6 | 98746 | 13.4 |
| 3593.798 | 2 | 22025 | 6.6 |
| 4754.854 | 5 | 11723 | 5.0 |
| 6576.056 | 13 | 46639 | 17.4 |
| 6802.753 | 30 | 144968 | 40.4 |
| 6898.095 | 100 | 540253 | 132.6 |
| 7201.334 | 35 | 173837 | 47.3 |
| 13842.945 | 3 | 1382 | 9.4 |

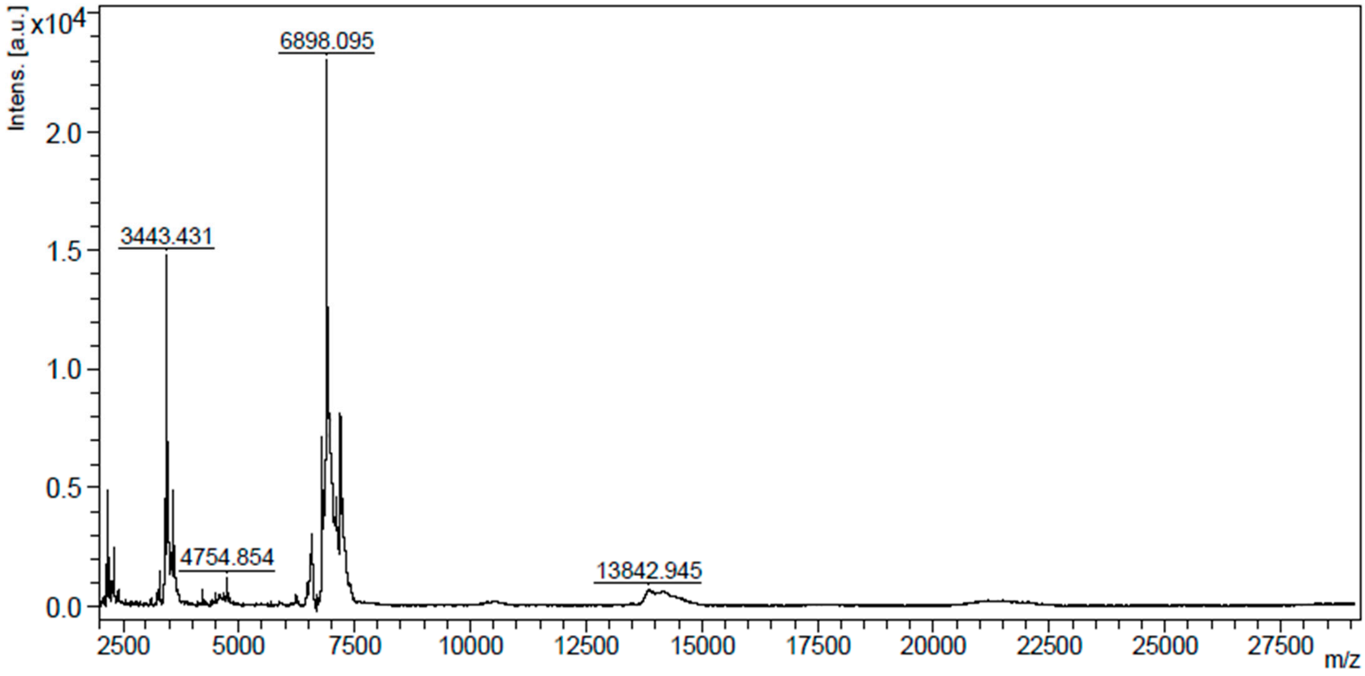


I-2-7 02122024\0_G9\1 2024-12-02T14:20:42.000

**Figure S34.** MALDI-TOF MS spectrum of siPCSK9_2_7-aS

| Name | Sequence (5′-3′) |
|---|---|
| siPCSK9_2_7-aS | mAmUmAmAfAfUfGmUmCmUmGmCfUfUfGmCmUmUmGmGmG |
| Calculated Mw (H+ form) | 6913,42 |
| Founded Mw | 6897,00 |

siPCSK9-2-8

| m/z | Rel. Intens. | Area | SN |
|---|---|---|---|
| 2298.407 | 14 | 2394 | 6.8 |
| 2533.723 | 8 | 7063 | 7.8 |
| 3445.786 | 6 | 10734 | 14.6 |
| 3596.594 | 3 | 5775 | 6.1 |
| 6588.099 | 6 | 1443 | 5.1 |
| 6910.051 | 100 | 50220 | 91.7 |
| 6946.987 | 40 | 19808 | 36.5 |
| 7207.035 | 1 | 2463 | 10.9 |
| 7251.692 | 26 | 14210 | 24.4 |

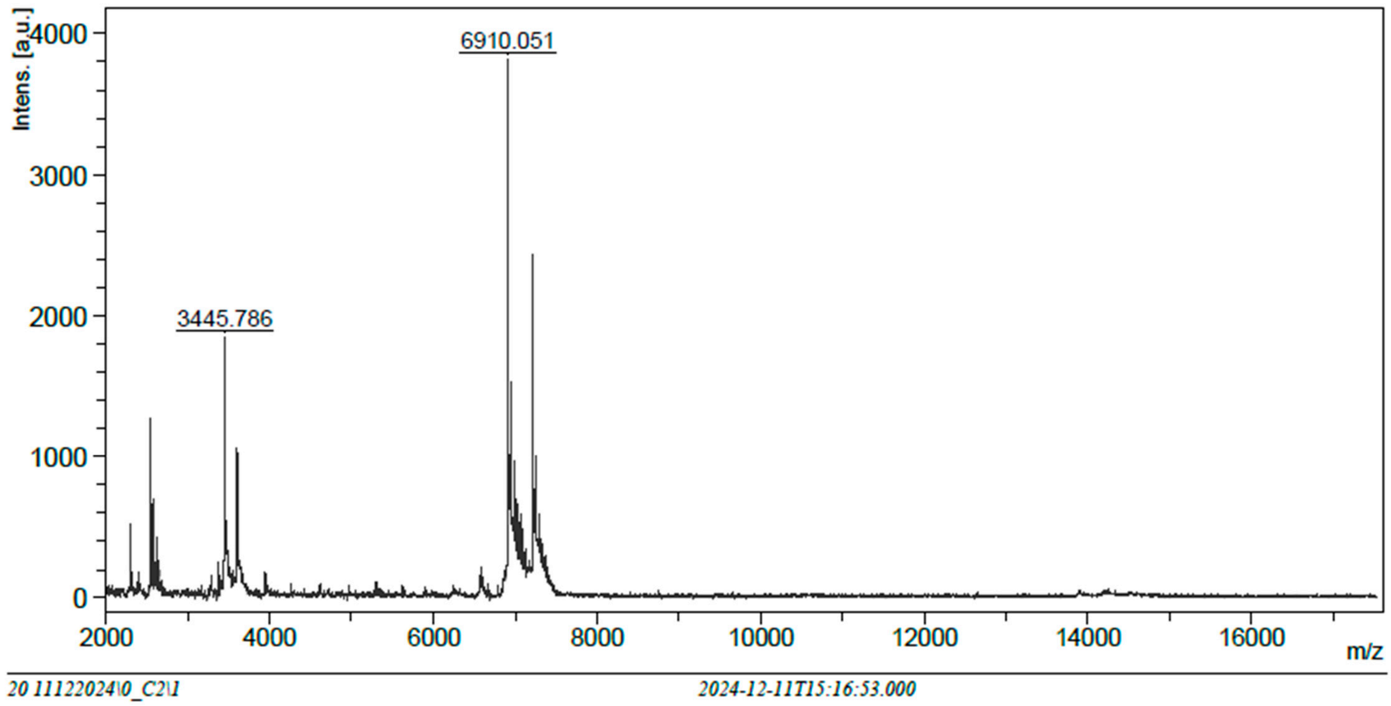


**Figure S35.** MALDI-TOF MS spectrum of siPCSK9_2_8-S

| Name | Sequence (5′-3′) |
|---|---|
| siPCSK9_2_8-S | mCmAmAmGmCmAmAmGmCmAfGfAfCmAmUmUmUmAmUmCmU |
| Calculated Mw (H+ form) | 6922,6 |
| Founded Mw | 6910,05 |

siPCSK9-2-8

| m/z | Rel. Intens. | Area | SN |
|---|---|---|---|
| 2296.879 | 7 | 10155 | 6.7 |
| 3395.293 | 2 | 32909 | 10.1 |
| 3442.329 | 6 | 85723 | 26.9 |
| 3593.381 | 2 | 20916 | 7.4 |
| 6574.534 | 13 | 39524 | 18.3 |
| 6801.322 | 35 | 170932 | 50.2 |
| 6897.723 | 100 | 617092 | 144.3 |
| 7201.812 | 34 | 180762 | 50.4 |

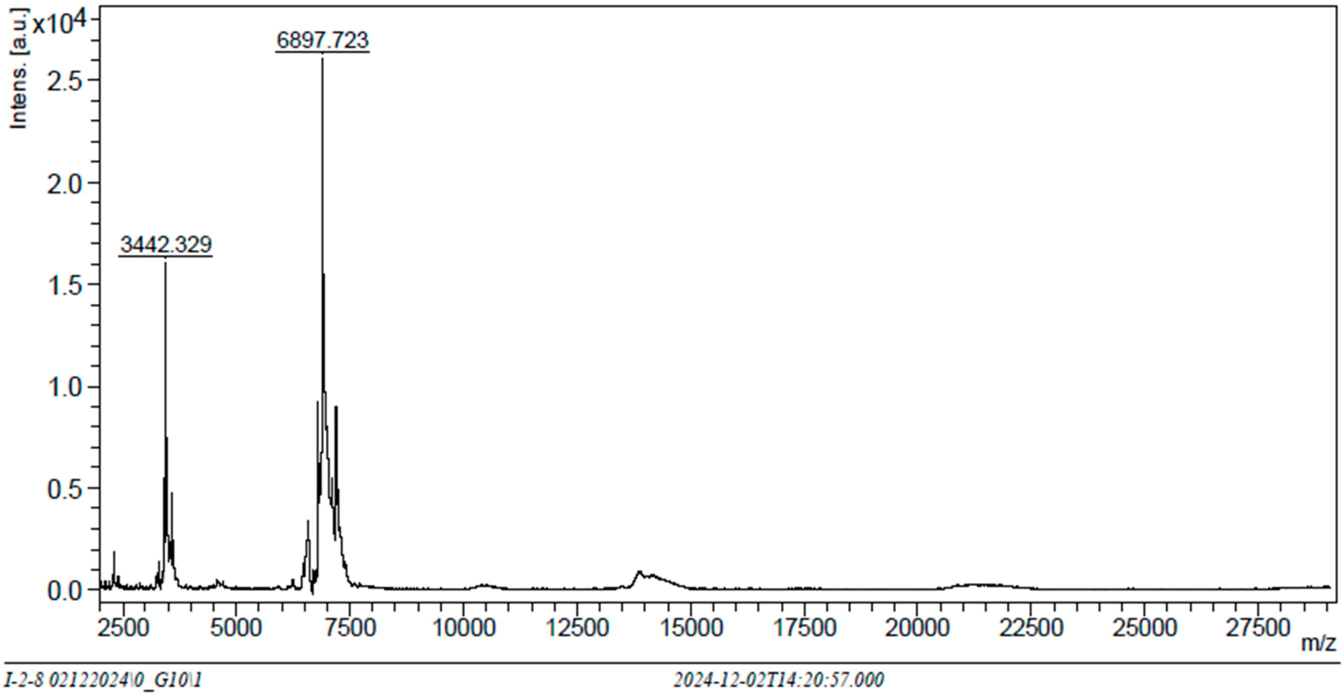


**Figure S36.** MALDI-TOF MS spectrum of siPCSK9_2_8-aS

| Name | Sequence (5′-3′) |
|---|---|
| siPCSK9_2_8-aS | mAfUmAmAmAfUmGfUmCmUmGfCmUfUmGmCmUmUmGfGmG |
| Calculated Mw (H+ form) | 6913,42 |
| Founded Mw | 6897,72 |

siPCSK9-2-9

| m/z | Rel. Intens. | Area | SN |
|---|---|---|---|
| 2297.146 | 5 | 10514 | 6.6 |
| 2533.623 | 7 | 11856 | 6.8 |
| 3446.094 | 11 | 27130 | 28.3 |
| 3596.773 | 6 | 15483 | 16.6 |
| 6587.339 | 6 | 7370 | 8.4 |
| 6908.215 | 100 | 139078 | 139.8 |
| 7205.043 | 1 | 7856 | 6.4 |

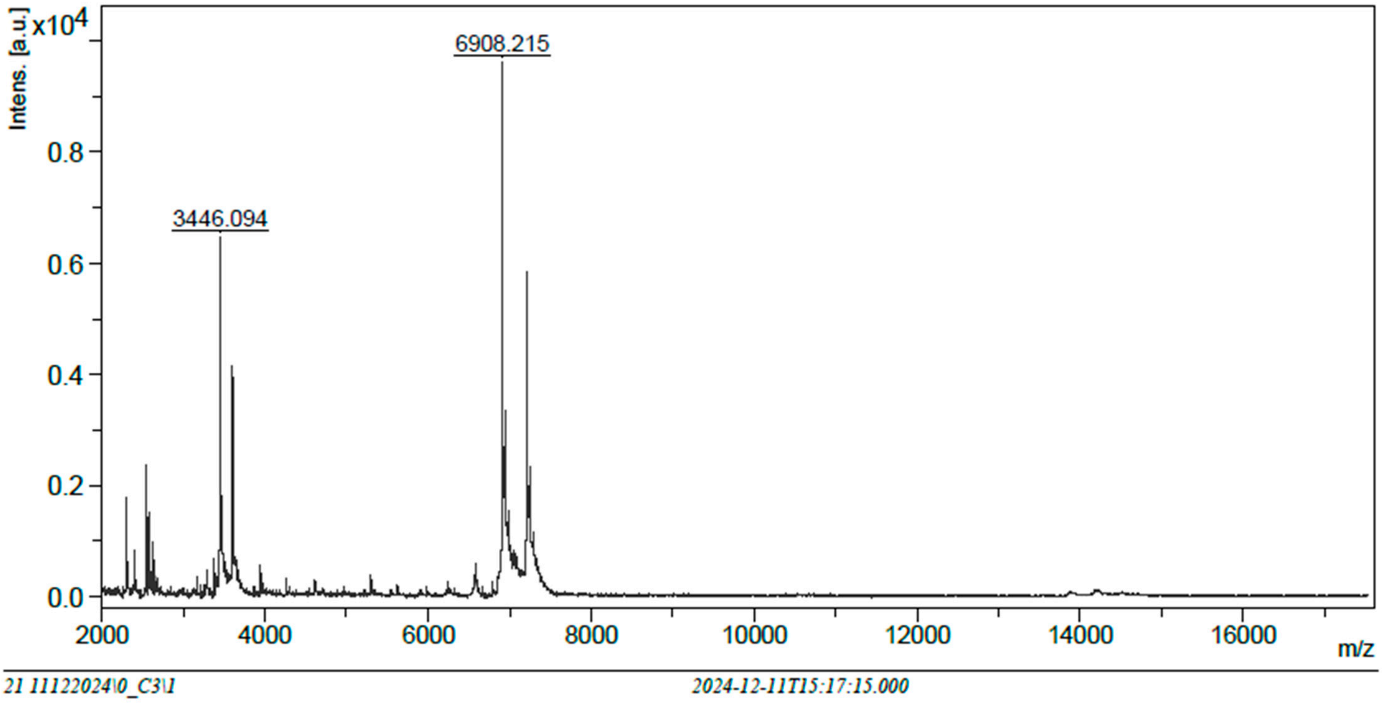


**Figure S37.** MALDI-TOF MS spectrum of siPCSK9_2_9-S

| Name | Sequence (5′-3′) |
|---|---|
| siPCSK9_2_9-S | mCmAmAmGmCmAmAmGmCmAfGfAfCmAmUmUmUmAmUmCmU |
| Calculated Mw (H+ form) | 6922,6 |
| Founded Mw | 6908,2 |

siPCSK9-2-9

| m/z | Rel. Intens. | Area | SN |
|---|---|---|---|
| 2525.299 | 9 | 8509 | 6.3 |
| 3468.910 | 11 | 16314 | 8.9 |
| 3737.860 | 2 | 21137 | 7.8 |
| 3784.827 | 6 | 63404 | 18.5 |
| 4610.286 | 7 | 10039 | 5.8 |
| 6588.220 | 4 | 7576 | 4.4 |
| 6931.681 | 23 | 46674 | 24.1 |
| 7262.457 | 14 | 27073 | 14.9 |
| 7489.438 | 36 | 105322 | 37.8 |
| 7585.199 | 100 | 504270 | 106.9 |
| 7888.954 | 19 | 44962 | 20.7 |

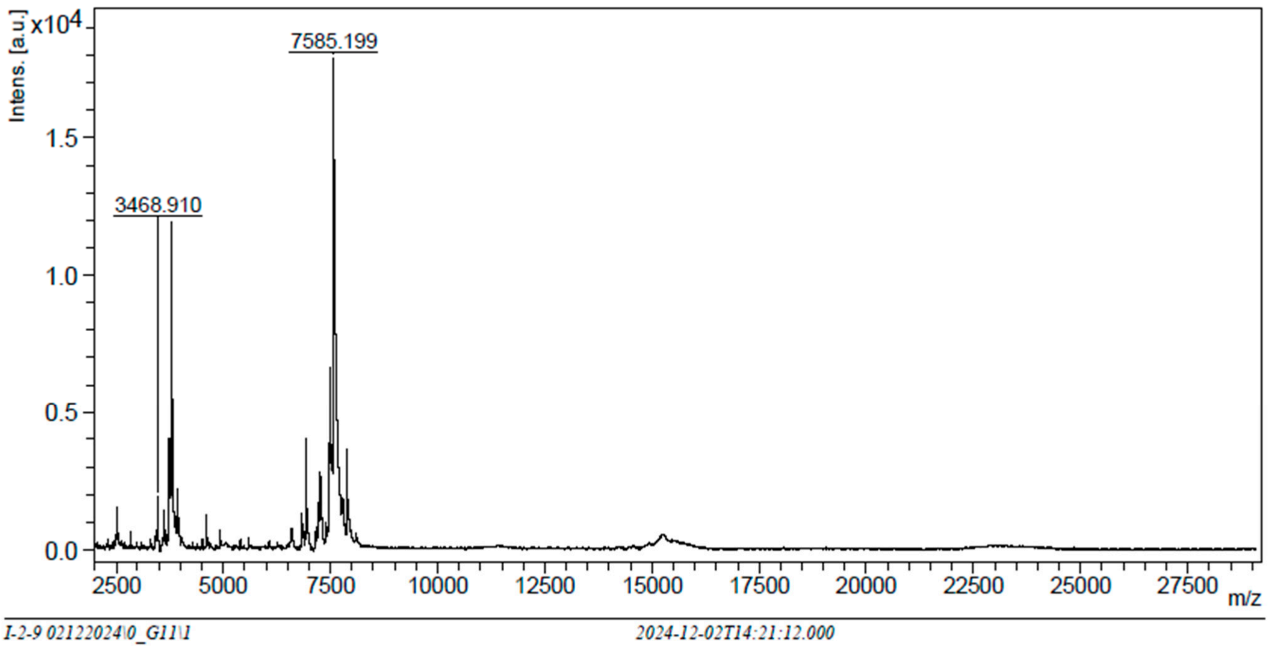


**Figure S38.** MALDI-TOF MS spectrum of siPCSK9_2_9-aS

| Name | Sequence (5′-3′) |
|---|---|
| siPCSK9_2_9-aS | mAfUmAmAmAfUmGfUmCmUmGfCmUfUmGmCmUmUmGfGmGmAmA |
| Calculated Mw (H+ form) | 7599,9 |
| Founded Mw | 7585,20 |

siPCSK9-2-10

| m/z | Rel. Intens. | Area | SN |
|---|---|---|---|
| 2299.486 | 11 | 6006 | 4.8 |
| 2399.662 | 8 | 2821 | 3.6 |
| 3449.570 | 51 | 38783 | 23.1 |
| 3459.291 | 34 | 32749 | 15.6 |
| 3600.460 | 51 | 47742 | 23.0 |
| 6586.027 | 12 | 14872 | 8.6 |
| 6906.509 | 100 | 172138 | 77.0 |
| 6945.286 | 75 | 141883 | 57.9 |
| 7210.489 | 83 | 156682 | 66.5 |
| 7248.937 | 62 | 120657 | 49.2 |

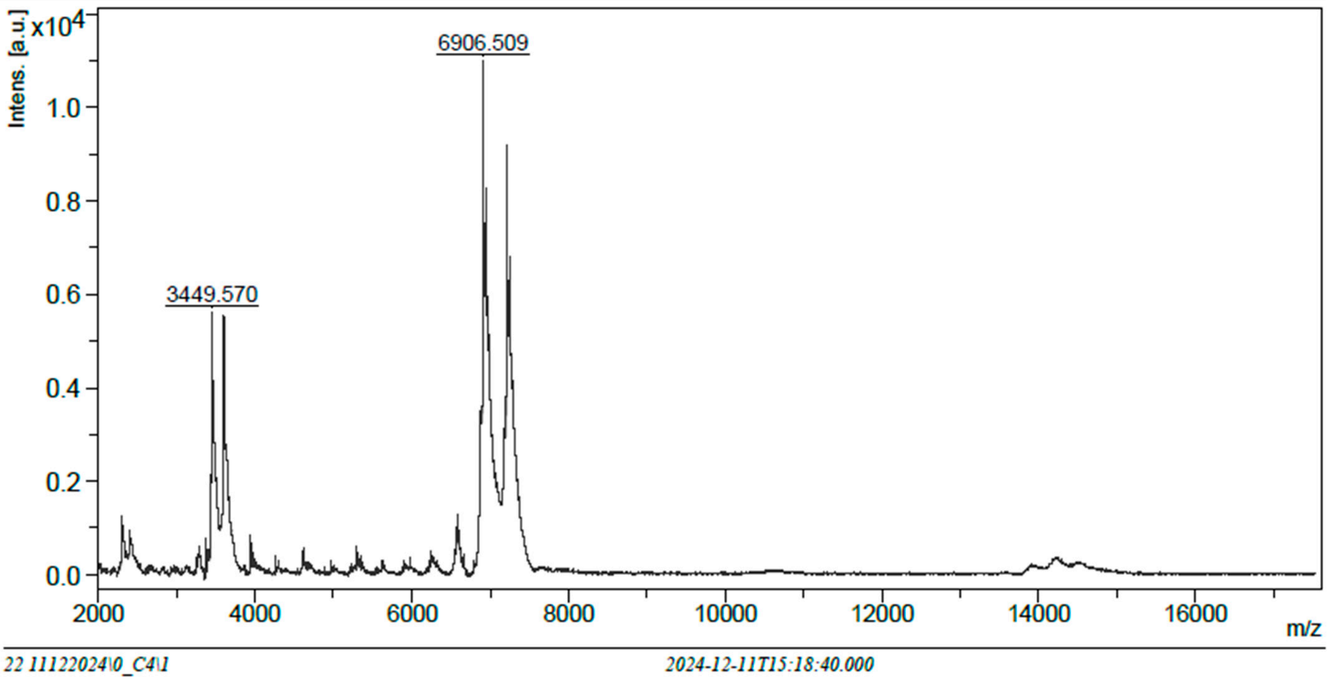


**Figure S39.** MALDI-TOF MS spectrum of siPCSK9_2_10-S

| Name | Sequence (5′-3′) |
|---|---|
| siPCSK9_2_10-S | mCmAmAmGmCmAmAmGmCmAfGfAfCmAmUmUmUmAmUmCmU |
| Calculated Mw (H+ form) | 6922,6 |
| Founded Mw | 6908,22 |

siPCSK9-2-10

| m/z | Rel. Intens. | Area | SN |
|---|---|---|---|
| 2308.617 | 7 | 6579 | 7.7 |
| 3463.486 | 49 | 89877 | 65.2 |
| 3614.089 | 13 | 19362 | 17.2 |
| 6612.637 | 19 | 41253 | 35.9 |
| 6933.898 | 100 | 380455 | 191.4 |
| 7238.095 | 28 | 73072 | 54.6 |
| 13935.043 | 3 | 1480 | 8.7 |

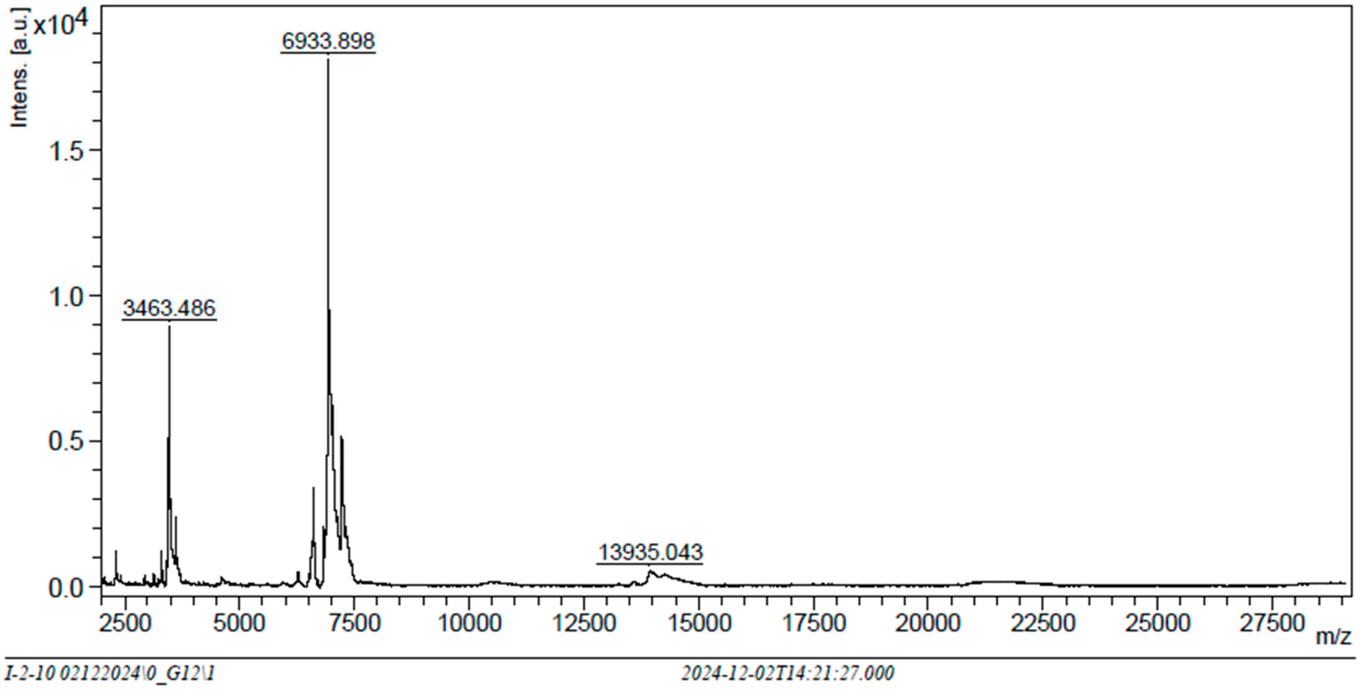


**Figure S40.** MALDI-TOF MS spectrum of siPCSK9_2_10-aS

| Name | Sequence (5′-3′) |
|---|---|
| siPCSK9_2_10-aS | mAmUmAmAmAmUmGmUfCfUfGmCmUmUmGmCmUmUmGmGmG |
| Calculated Mw (H+ form) | 6949,54 |
| Founded Mw | 7585,20 |

siPCSK9-2-11

| m/z | Rel. Intens. | Area | SN |
|---|---|---|---|
| 2298.325 | 41 | 3824 | 7.1 |
| 3446.366 | 20 | 20534 | 13.1 |
| 3597.035 | 13 | 9751 | 8.7 |
| 3882.948 | 31 | 18334 | 14.7 |
| 4551.241 | 100 | 15758 | 20.3 |
| 6587.886 | 23 | 3108 | 6.3 |
| 6903.710 | 8 | 8524 | 31.8 |
| 7206.688 | 4 | 4603 | 22.7 |
| 7251.720 | 93 | 23301 | 27.4 |

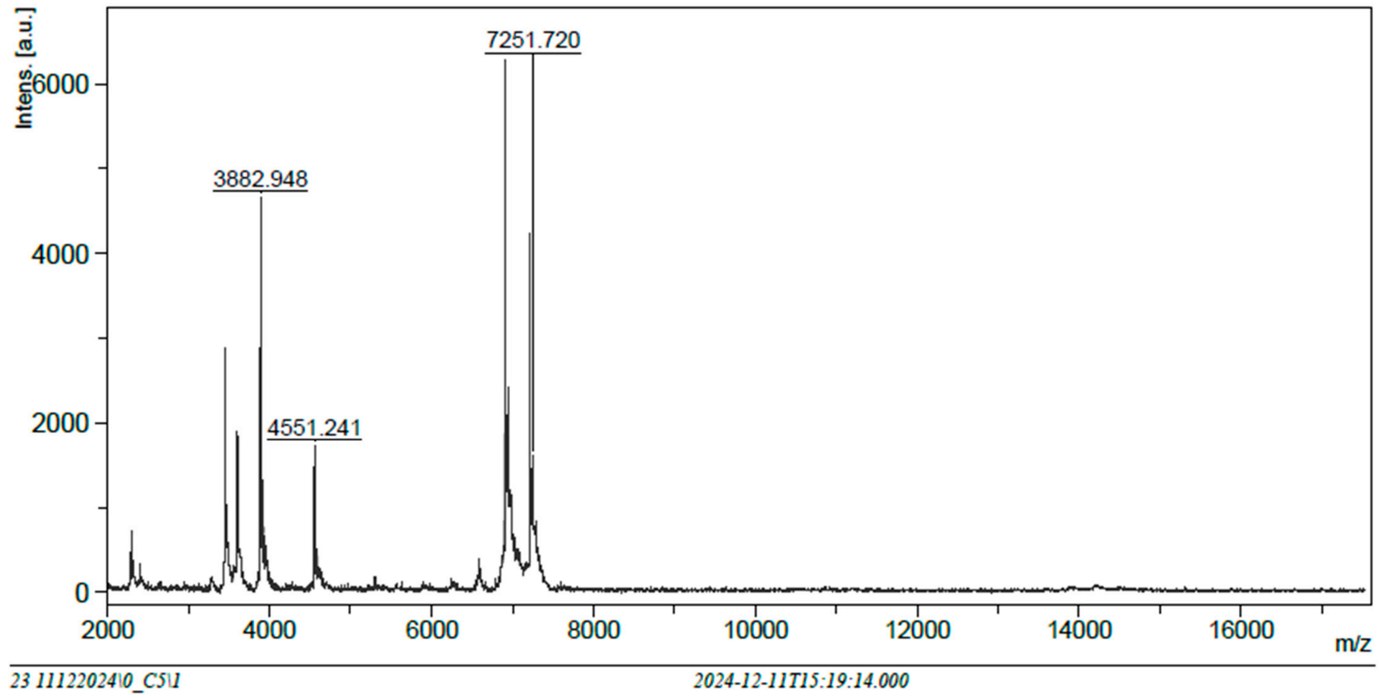


**Figure S41.** MALDI-TOF MS spectrum of siPCSK9_2_11 -S

| Name | Sequence (5′-3′) |
|---|---|
| siPCSK9_2_11-S | mCmAmAmGmCmAfAmGfCfAmGmAmCmAmUmUmUmAmUmCmU |
| Calculated Mw (H+ form) | 6922,6 |
| Founded Mw | 6903,71 |

siPCSK9-2-11

| m/z | Rel. Intens. | Area | SN |
|---|---|---|---|
| 2284.116 | 9 | 4687 | 9.4 |
| 3424.203 | 6 | 31820 | 24.7 |
| 5532.703 | 0 | 1484 | 6.4 |
| 6762.504 | 0 | 1639 | 10.5 |
| 6863.437 | 100 | 112689 | 151.4 |
| 7166.947 | 11 | 10948 | 16.5 |

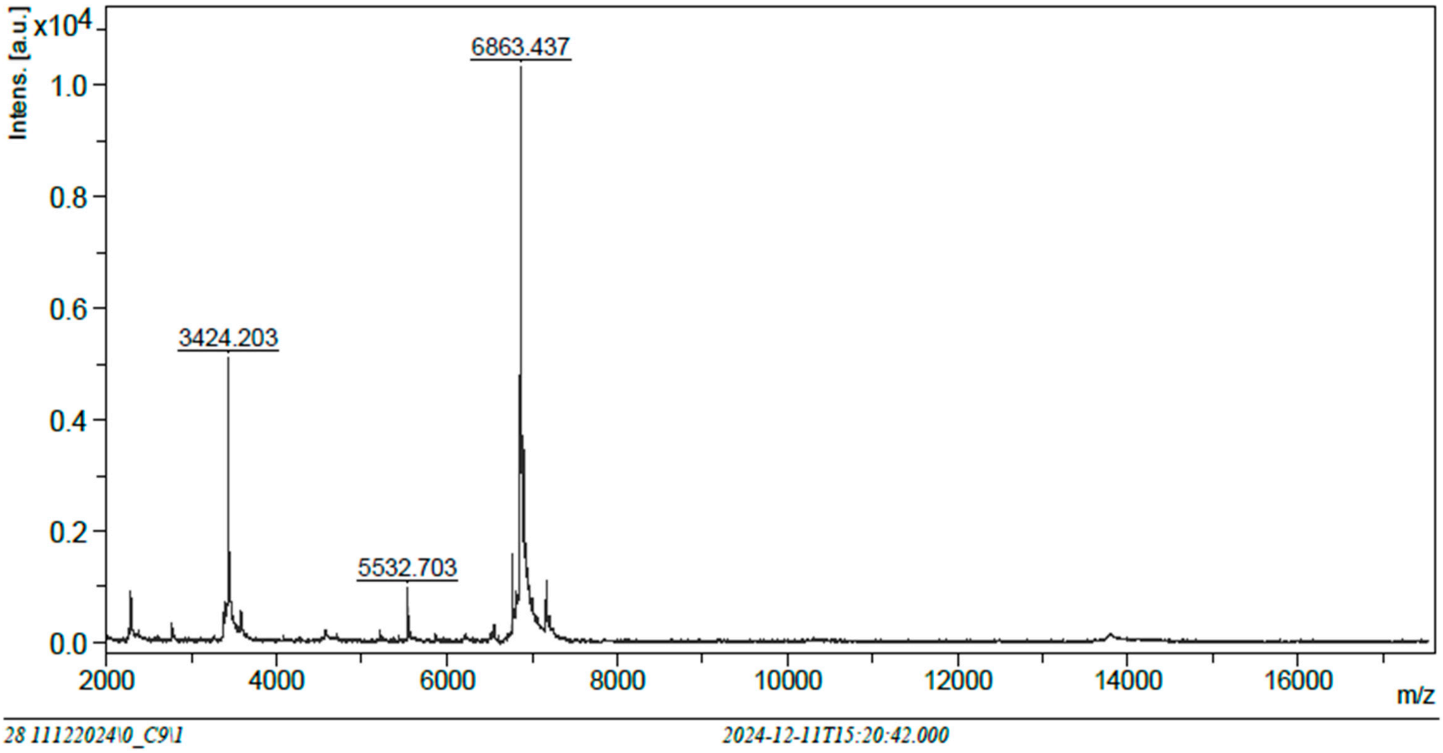


**Figure S42.** MALDI-TOF MS spectrum of siPCSK9_2_11-aS

| Name | Sequence (5′-3′) |
|---|---|
| siPCSK9_2_11-aS | mAfUmAfAfAfUmGfUmCfUmGmCmUfUmGfCmUfUmGmGmG |
| Calculated Mw (H+ form) | 6877,3 |
| Founded Mw | 6863,44 |

siPCSK9-2-12

| m/z | Rel. Intens. | Area | SN |
|---|---|---|---|
| 2294.678 | 14 | 6912 | 10.8 |
| 3440.170 | 8 | 21626 | 22.0 |
| 3590.812 | 5 | 14763 | 15.6 |
| 6896.762 | 100 | 90299 | 132.1 |
| 6935.012 | 32 | 33099 | 41.6 |
| 7193.762 | 1 | 5084 | 36.3 |

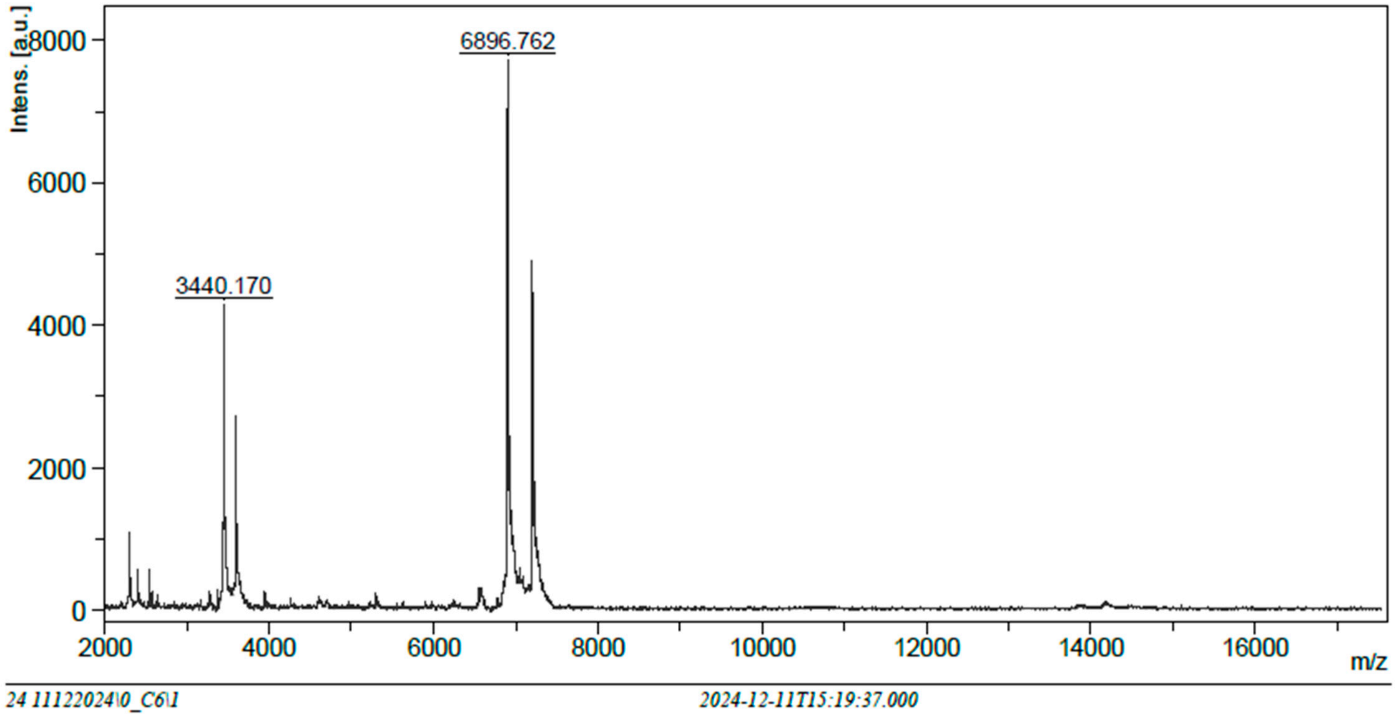


**Figure S43.** MALDI-TOF MS spectrum of siPCSK9_2_12-S

| Name | Sequence (5′-3′) |
|---|---|
| siPCSK9_2_12-S | fCmAmAmGmCmAmAmGmCmAfGfAfCmAmUmUmUmAmUmCmU |
| Calculated Mw (H+ form) | 6910,56 |
| Founded Mw | 6896,76 |

siPCSK9-2-12

| m/z | Rel. Intens. | Area | SN |
|---|---|---|---|
| 2287.860 | 5 | 1569 | 4.4 |
| 3434.148 | 48 | 45949 | 45.8 |
| 6564.781 | 13 | 23336 | 15.7 |
| 6778.533 | 34 | 66863 | 40.9 |
| 6873.908 | 100 | 262659 | 119.1 |
| 7177.617 | 25 | 45677 | 30.8 |
| 13815.919 | 4 | 2256 | 7.3 |

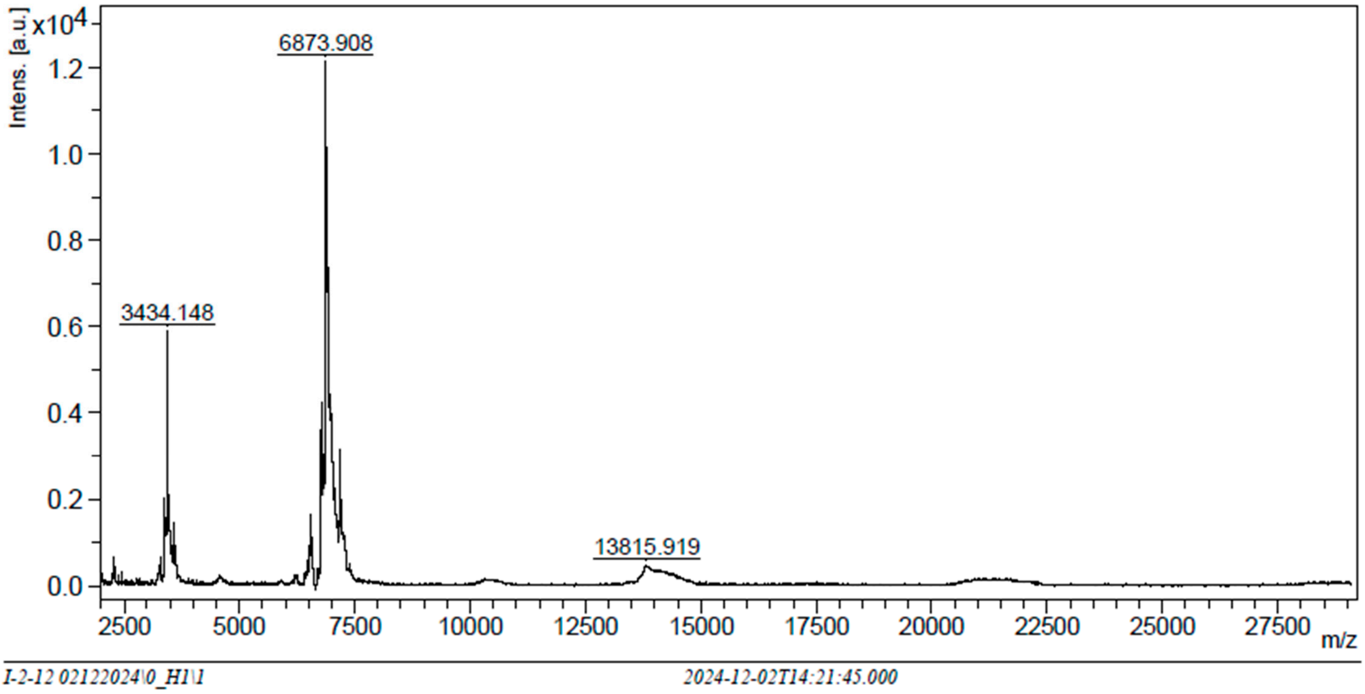


**Figure S44.** MALDI-TOF MS spectrum of siPCSK9_2_12-aS

| Name | Sequence (5′-3′) |
|---|---|
| siPCSK9_2_12-aS | mAfUmAfAfAfUmGfUmCfUmGmCmUfUmGfCmUfUmGmGmG |
| Calculated Mw (H+ form) | 6889,34 |
| Founded Mw | 6873,91 |

siPCSK9-6-1

| m/z | Rel. Intens. | Area | SN |
|---|---|---|---|
| 2311.834 | 12 | 2408 | 5.4 |
| 2412.129 | 12 | 2365 | 5.4 |
| 3308.885 | 7 | 1538 | 3.6 |
| 3464.733 | 8 | 12192 | 14.6 |
| 3618.722 | 67 | 21722 | 34.3 |
| 5019.599 | 5 | 701 | 2.8 |
| 6622.623 | 13 | 6009 | 9.5 |
| 6942.549 | 90 | 49053 | 70.8 |
| 7246.078 | 100 | 61546 | 79.0 |
| 14235.154 | 2 | 168 | 2.3 |

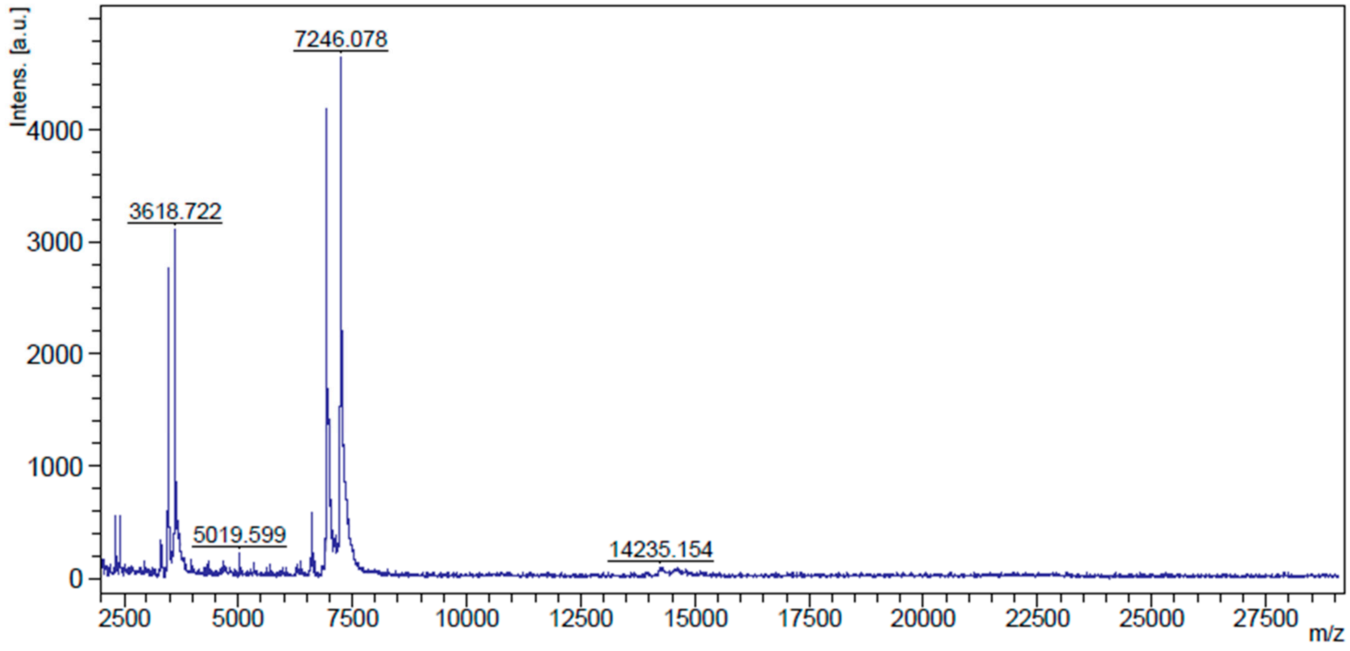


25 13122024\0_F10\1 2024-12-13T13:19:04.000

**Figure S45.** MALDI-TOF MS spectrum of siPCSK9_6_1-S

| Name | Sequence (5′-3′) |
|---|---|
| siPCSK9_6_1-S | mCmCmAmAmGmCmAmAmGmCmAmGmAmCmAmUmUmUmAmUmC |
| Calculated Mw (H+ form) | 6957,73 |
| Founded Mw | 6942,55 |

siPCSK9-6-1

| m/z | Rel. Intens. | Area | SN |
|---|---|---|---|
| 2312.293 | 7 | 6687 | 7.6 |
| 3466.574 | 5 | 69937 | 15.3 |
| 3616.670 | 3 | 31595 | 16.1 |
| 6623.781 | 25 | 60566 | 48.1 |
| 6944.713 | 100 | 294340 | 190.3 |
| 7241.773 | 1 | 19703 | 8.1 |
| 14265.898 | 3 | 1095 | 10.1 |

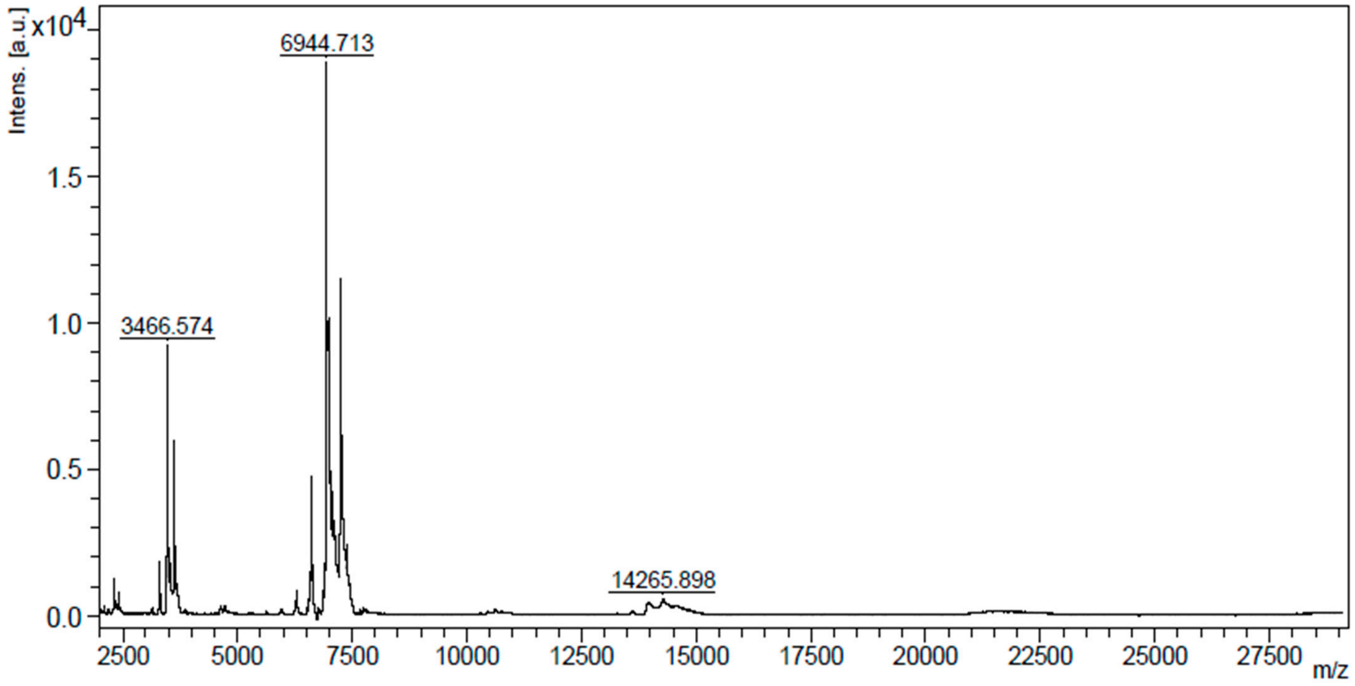


I-6-1 02122024\0_H2\1 2024-12-02T14:22:02.000

**Figure S46.** MALDI-TOF MS spectrum of siPCSK9_6_1-aS

| Name | Sequence (5′-3′) |
|---|---|
| siPCSK9_6_1-aS | mUmAmAmAmUmGmUmCmUmGmCmUmUmGmCmUmUmGmGmGmU |
| Calculated Mw (H+ form) | 6962,62 |
| Founded Mw | 6944,71 |

siPCSK9-6-2

| m/z | Rel. Intens. | Area | SN |
|---|---|---|---|
| 3289.683 | 13 | 2002 | 6.7 |
| 3448.812 | 51 | 18009 | 26.8 |
| 3621.891 | 17 | 5321 | 8.8 |
| 6586.336 | 17 | 8611 | 12.2 |
| 6906.804 | 100 | 47527 | 72.3 |
| 7210.050 | 14 | 5952 | 10.2 |

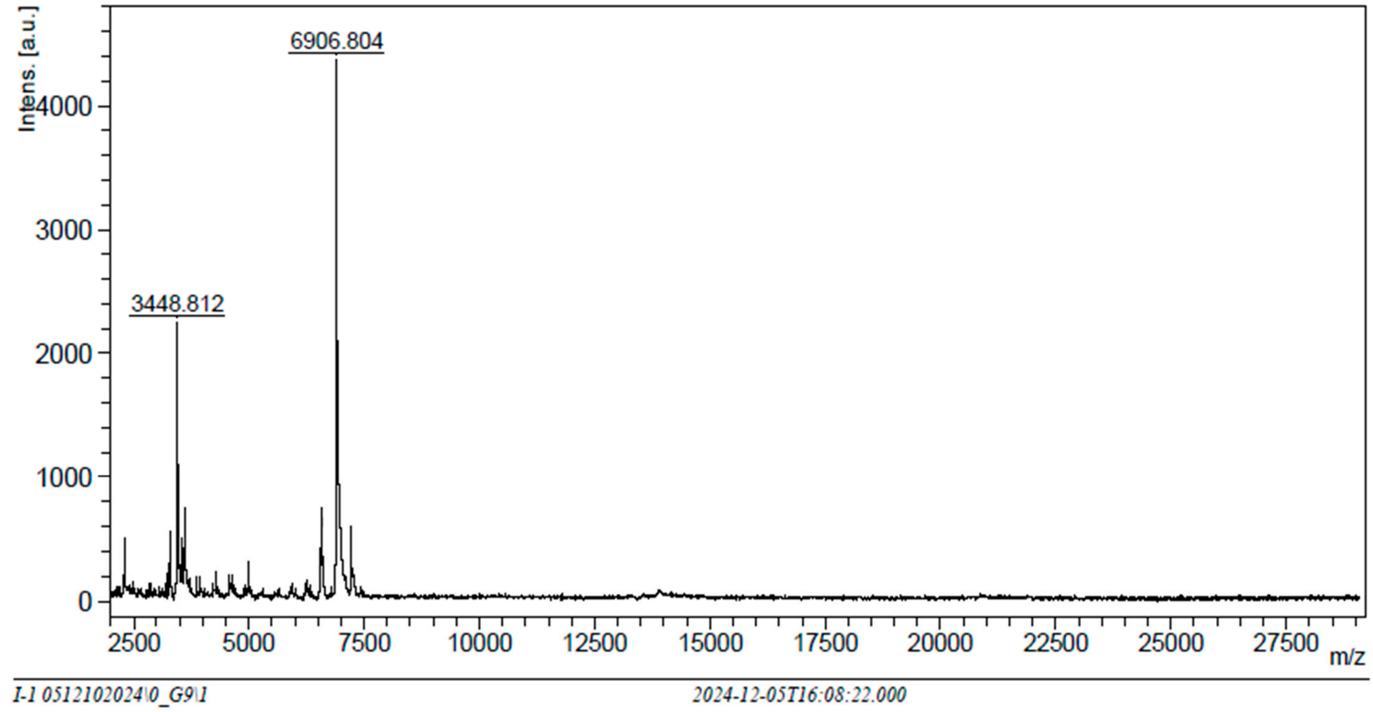


**Figure S47.** MALDI-TOF MS spectrum of siPCSK9_6_2-S

| Name | Sequence (5′-3′) |
|---|---|
| siPCSK9_6_2-S | mCmCmAmAmGmCmAmAmGmCfAfGfAmCmAmUmUmUmAmUmC |
| Calculated Mw (H+ form) | 6921,61 |
| Founded Mw | 6906,80 |

siPCSK9-6-2

| m/z | Rel. Intens. | Area | SN |
|---|---|---|---|
| 2320.476 | 21 | 7094 | 9.1 |
| 3311.023 | 13 | 6931 | 6.3 |
| 3470.017 | 87 | 58912 | 44.5 |
| 3620.273 | 14 | 12039 | 7.3 |
| 6624.395 | 18 | 22917 | 15.2 |
| 6944.916 | 100 | 156624 | 85.5 |
| 7248.599 | 20 | 28014 | 17.3 |
| 13970.881 | 3 | 957 | 4.2 |

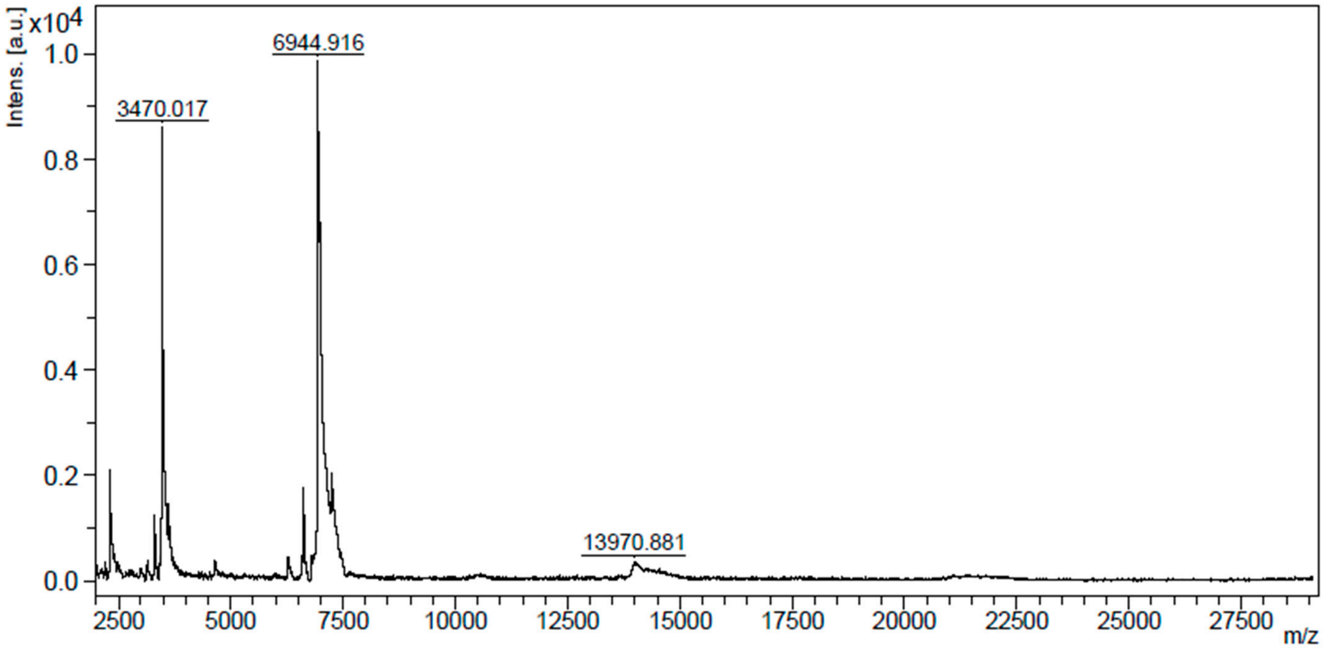


I-3 05121020240_G111 2024-12-05T16:09:06.000

**Figure S48.** MALDI-TOF MS spectrum of siPCSK9_6_2-aS

| Name | Sequence (5′-3′) |
|---|---|
| siPCSK9_6_2-aS | mUmAmAmAmUmGmUmCmUmGmCmUmUmGmCmUmUmGmGmGmU |
| Calculated Mw (H+ form) | 6962,62 |
| Founded Mw | 6944,92 |

siPCSK9-6-3

| m/z | Rel. Intens. | Area | SN |
|---|---|---|---|
| 2299.071 | 8 | 3863 | 8.6 |
| 3446.367 | 6 | 24838 | 20.2 |
| 3596.911 | 4 | 15674 | 14.9 |
| 6587.453 | 9 | 8282 | 13.1 |
| 6907.934 | 100 | 116632 | 158.7 |
| 6928.666 | 29 | 33147 | 46.7 |
| 7204.243 | 1 | 6922 | 19.9 |

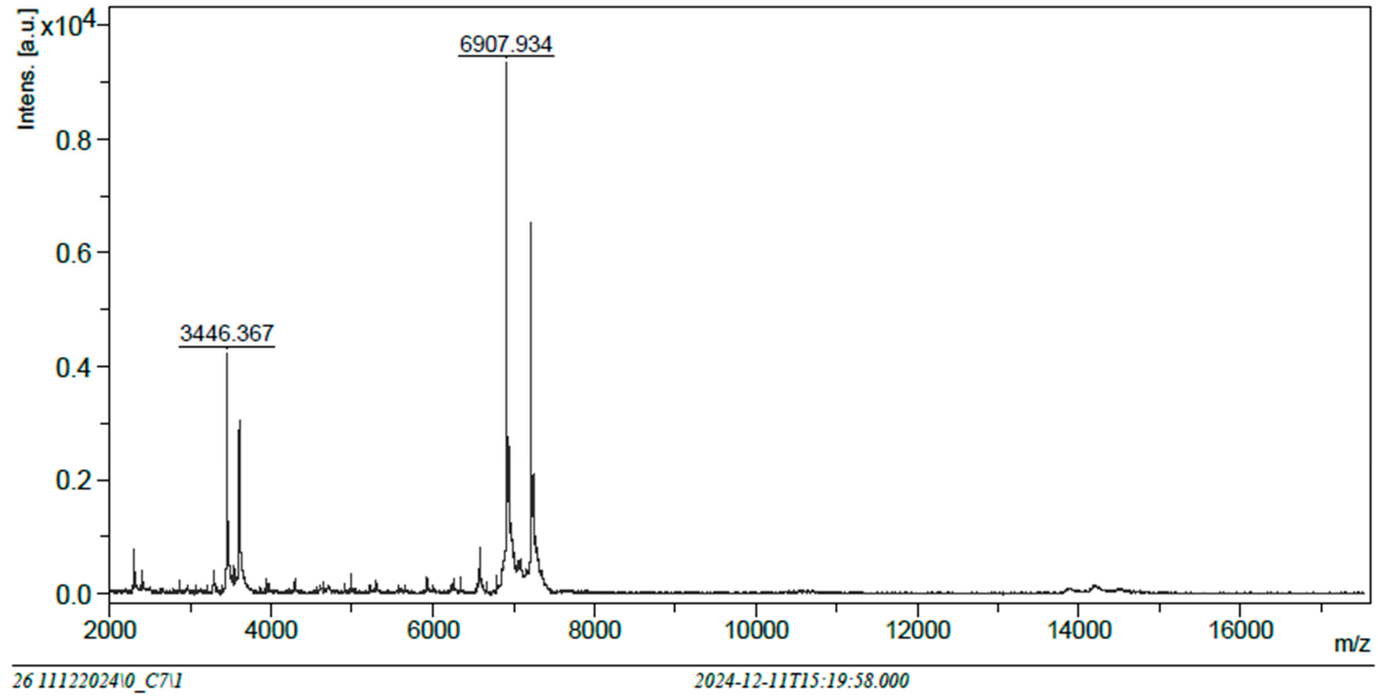


**Figure S49.** MALDI-TOF MS spectrum of siPCSK9_6_3-S

| Name | Sequence (5′-3′) |
|---|---|
| siPCSK9_6_3-S | mCmCmAmAmGmCmAmAmGmCfAfGfAmCmAmUmUmUmAmUmC |
| Calculated Mw (H+ form) | 6921,61 |
| Founded Mw | 6907,9 |

siPCSK9-6-3

| m/z | Rel. Intens. | Area | SN |
|---|---|---|---|
| 2442.148 | 13 | 12485 | 7.0 |
| 3426.739 | 66 | 32320 | 20.2 |
| 6537.943 | 16 | 9337 | 8.3 |
| 6879.879 | 100 | 71817 | 54.0 |
| 13812.674 | 6 | 1544 | 5.6 |

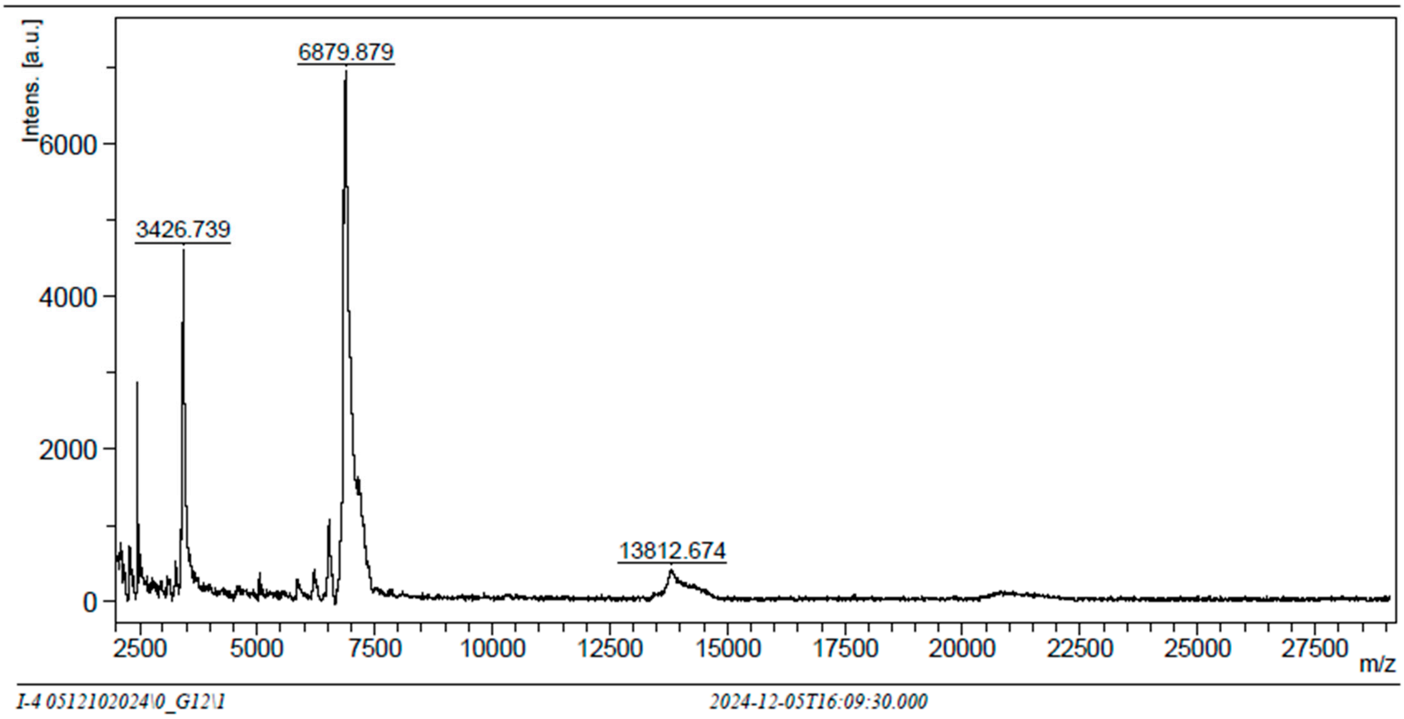


**Figure S50.** MALDI-TOF MS spectrum of siPCSK9_6_3-aS

| Name | Sequence (5′-3′) |
|---|---|
| siPCSK9_6_3-aS | mUfAmAfAmUfGmUfCmUmGmCfUmUfGmCfUmUfGmGfGmU |
| Calculated Mw (H+ form) | 6854,26 |
| Founded Mw | 6879,88 |

siPCSK9-6-4

| m/z | Rel. Intens. | Area | SN |
|---|---|---|---|
| 3446.143 | 4 | 40079 | 11.6 |
| 3597.661 | 3 | 17070 | 10.1 |
| 6586.334 | 12 | 19395 | 14.0 |
| 6906.419 | 100 | 197082 | 119.0 |
| 7203.451 | 1 | 12138 | 26.0 |
| 14161.729 | 2 | 1998 | 3.7 |

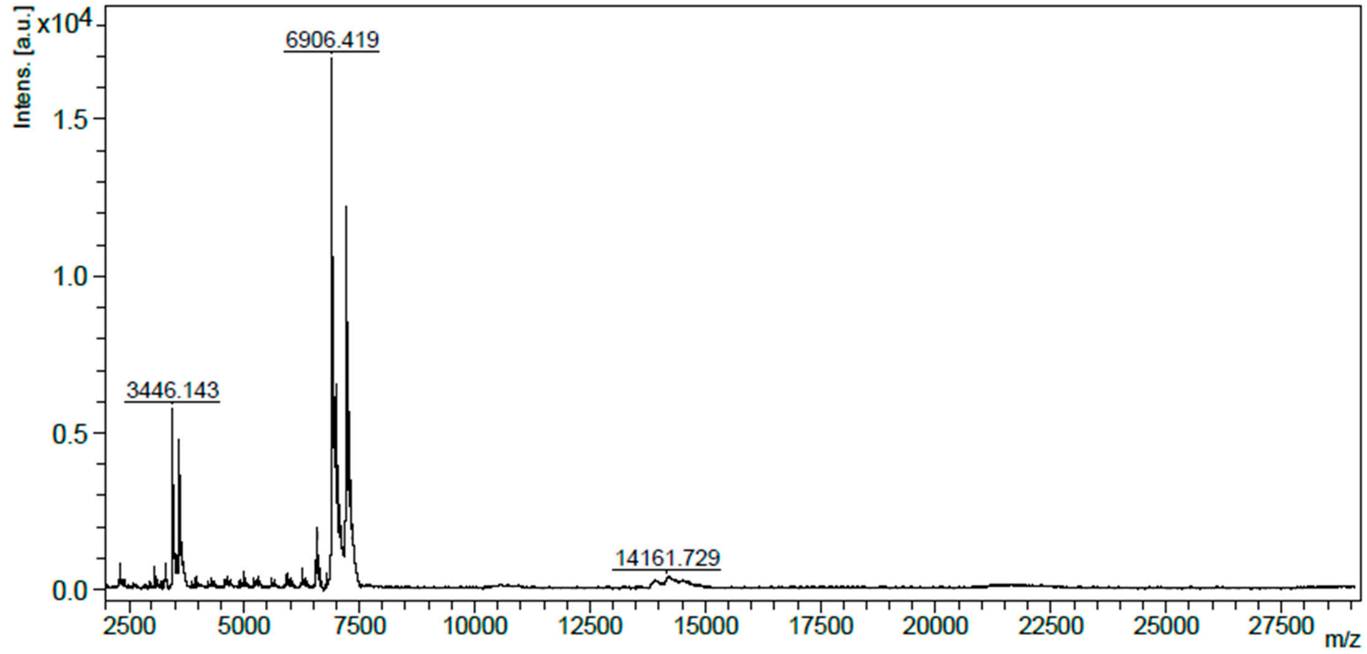


7 12122024\0_D4\1 2024-12-12T15:18:48.000

**Figure S51.** MALDI-TOF MS spectrum of siPCSK9_6_4-S

| Name | Sequence (5′-3′) |
|---|---|
| siPCSK9_6_4-S | mCmCmAmAmGmCmAmAmGmCfAfGfAmCmAmUmUmUmAmUmC |
| Calculated Mw (H+ form) | 6921,61 |
| Founded Mw | 6906,42 |

siPCSK9-6-4

| m/z | Rel. Intens. | Area | SN |
|---|---|---|---|
| 3274.708 | 11 | 6358 | 7.0 |
| 3434.531 | 84 | 56350 | 55.9 |
| 6553.884 | 18 | 25861 | 19.4 |
| 6875.198 | 100 | 186435 | 108.3 |
| 7176.322 | 12 | 5974 | 13.0 |
| 13823.481 | 3 | 1241 | 6.0 |

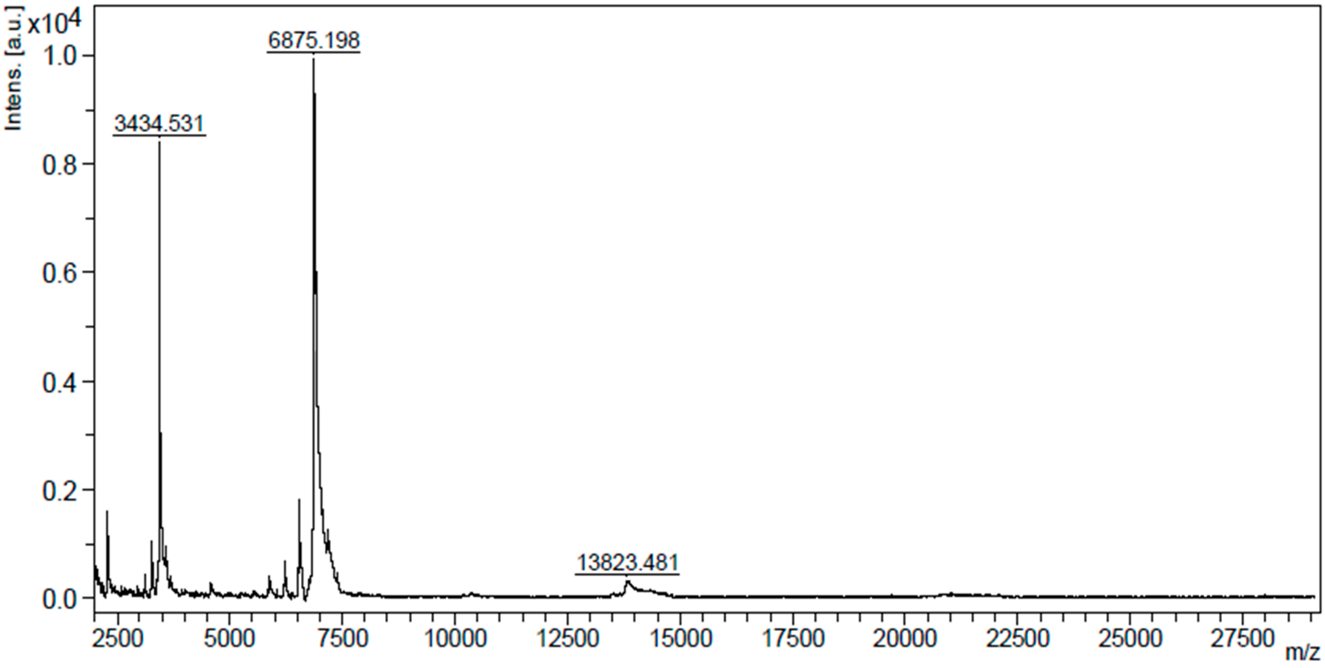


I-5 051210202410_H1\1 2024-12-05T16:09:54.000

**Figure S52.** MALDI-TOF MS spectrum of siPCSK9_6_4-aS

| Name | Sequence (5′-3′) |
|---|---|
| siPCSK9_6_4-aS | mUfAfAfAmUmGmUmCmUmGmCmUmUmGmCmUmUfGfGfGmU |
| Calculated Mw (H+ form) | 6890,38 |
| Founded Mw | 6875,20 |

siPCSK9-6-5

| m/z | Rel. Intens. | Area | SN |
|---|---|---|---|
| 3446.472 | 6 | 27654 | 17.0 |
| 3597.122 | 3 | 17135 | 12.4 |
| 6584.422 | 14 | 28094 | 17.7 |
| 6904.919 | 100 | 247924 | 125.0 |
| 14225.284 | 4 | 3811 | 8.9 |

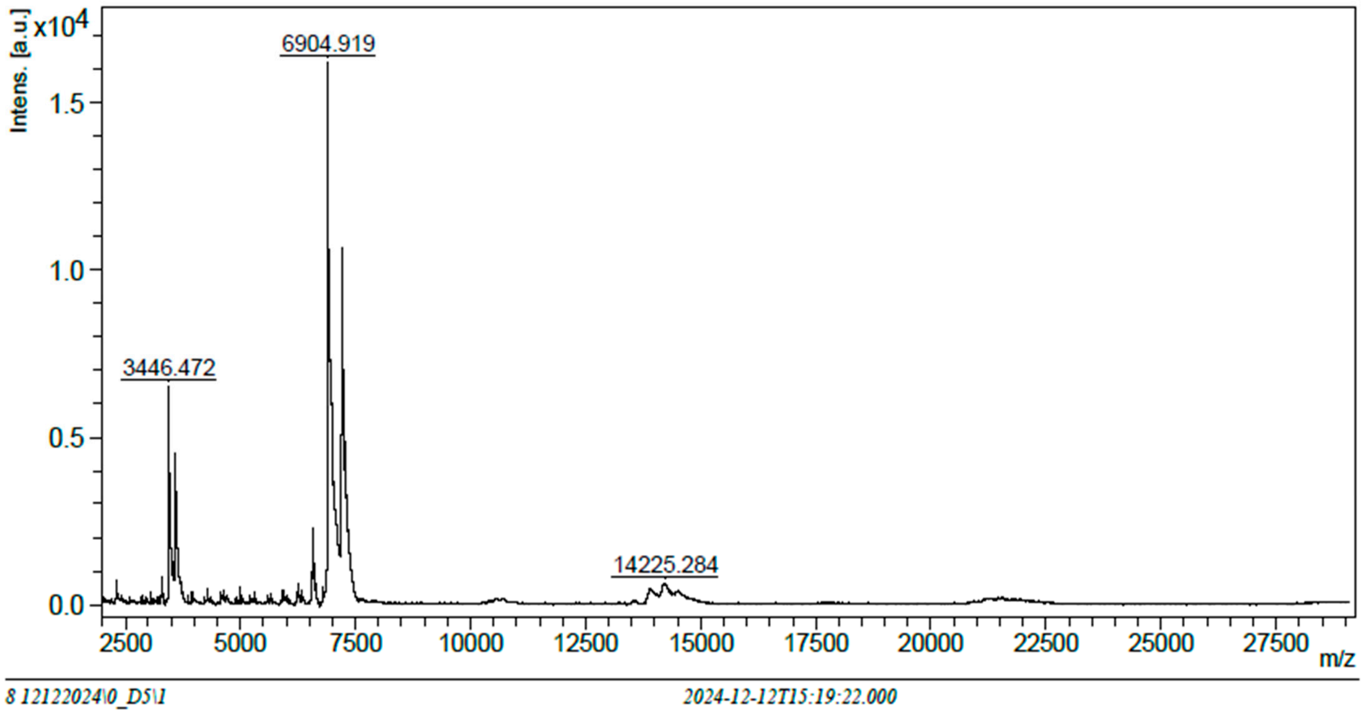


**Figure S53.** MALDI-TOF MS spectrum of siPCSK9_6_5-S

| Name | Sequence (5′-3′) |
|---|---|
| siPCSK9_6_5-S | mCmCmAmAmGmCmAmAmGmCfAfGfAmCmAmUmUmUmAmUmC |
| Calculated Mw (H+ form) | 6921,61 |
| Founded Mw | 6904,92 |

siPCSK9-6-5

| m/z | Rel. Intens. | Area | SN |
|---|---|---|---|
| 3432.901 | 69 | 27977 | 17.1 |
| 6550.668 | 15 | 5832 | 6.1 |
| 6871.404 | 100 | 109000 | 41.1 |
| 7153.351 | 35 | 15182 | 15.1 |
| 13826.724 | 5 | 1413 | 3.8 |

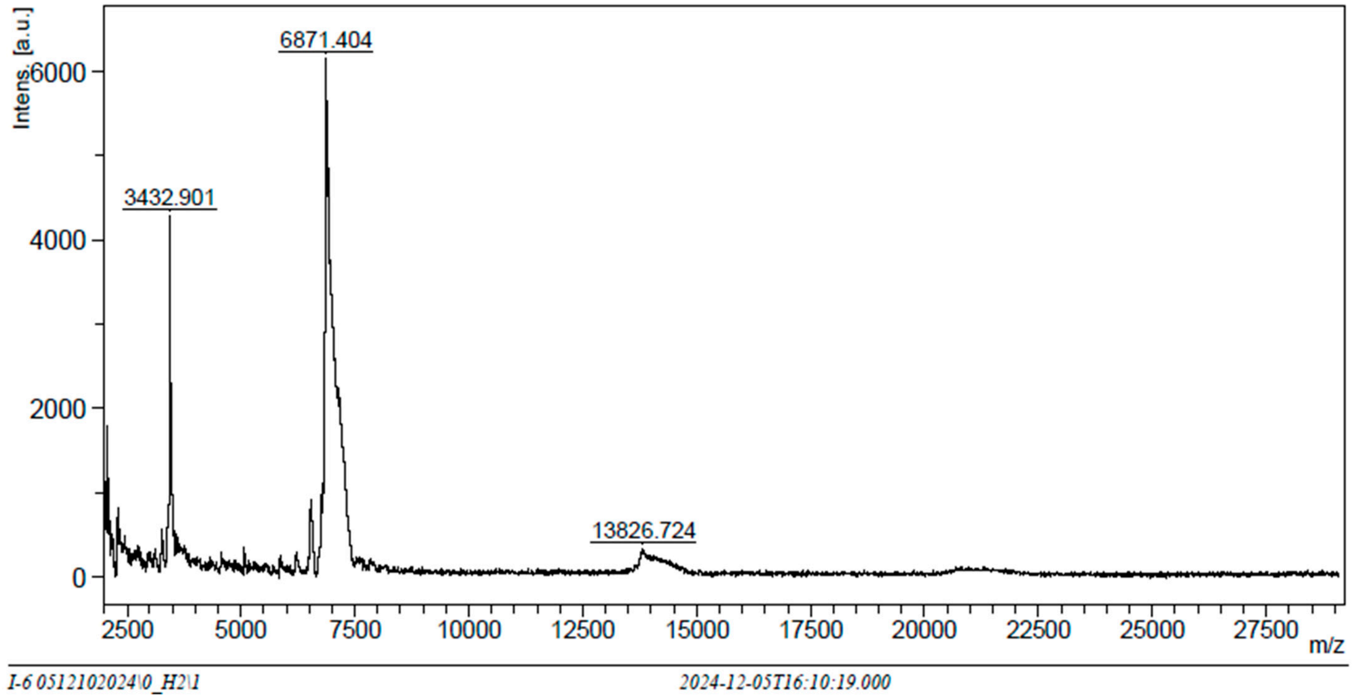


**Figure S54.** MALDI-TOF MS spectrum of siPCSK9_6_5-aS

| Name | Sequence (5′-3′) |
|---|---|
| siPCSK9_6_5-aS | mUfAmAfAfUfGmUmCmUmGmCmUmUmGmCfUfUfGmGfGmU |
| Calculated Mw (H+ form) | 6866,3 |
| Founded Mw | 6871,40 |

siPCSK9-6-6

| m/z | Rel. Intens. | Area | SN |
|---|---|---|---|
| 2299.456 | 5 | 3557 | 8.4 |
| 3446.687 | 5 | 35681 | 28.8 |
| 3597.698 | 4 | 22616 | 20.4 |
| 6584.688 | 12 | 21764 | 23.1 |
| 6906.173 | 100 | 247764 | 193.0 |
| 7202.738 | 1 | 17751 | 6.2 |
| 14163.913 | 4 | 2784 | 11.6 |

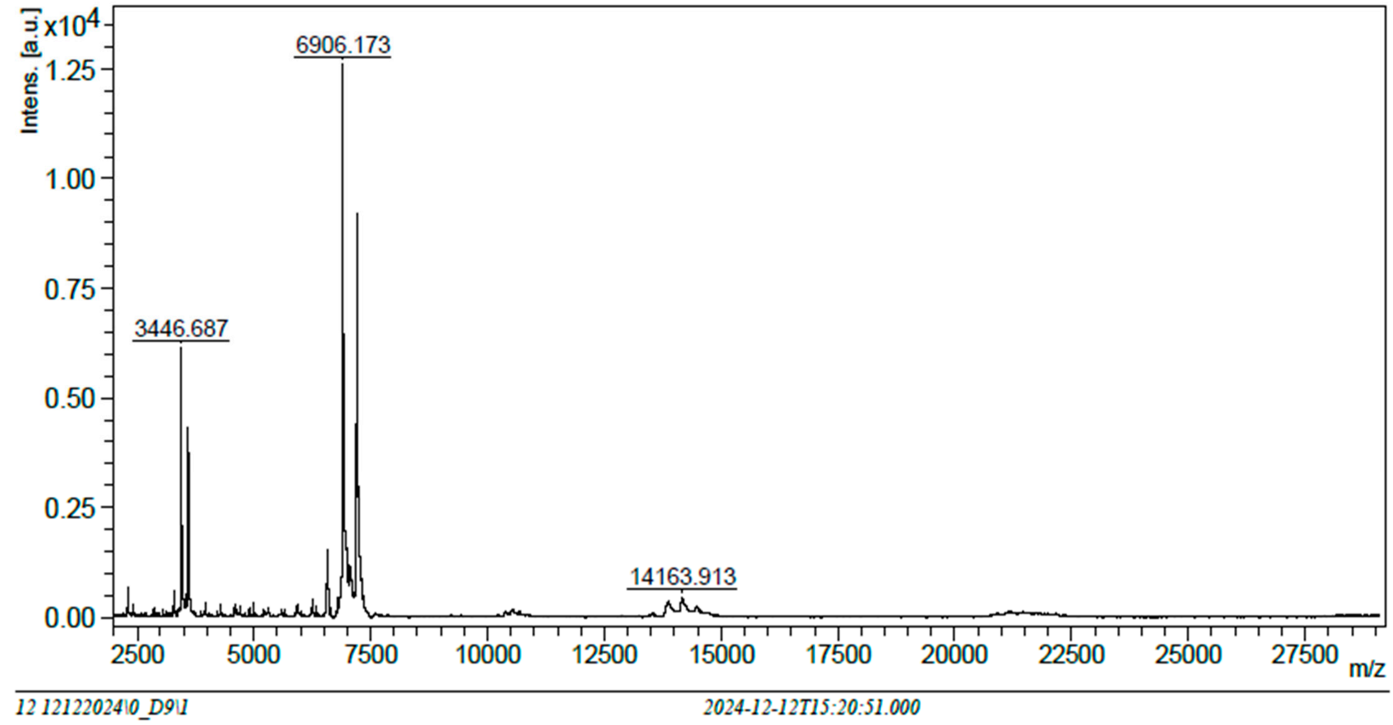


**Figure S55.** MALDI-TOF MS spectrum of siPCSK9_6_6-S

| Name | Sequence (5′-3′) |
|---|---|
| siPCSK9_6_6-S | mCmCmAmAmGmCmAmAmGmCfAfGfAmCmAmUmUmUmAmUmC |
| Calculated Mw (H+ form) | 6921,61 |
| Founded Mw | 6906,17 |

siPCSK9-6-6

| m/z | Rel. Intens. | Area | SN |
|---|---|---|---|
| 2477.880 | 7 | 14477 | 7.2 |
| 3383.636 | 2 | 5653 | 6.0 |
| 3431.116 | 9 | 53322 | 18.8 |
| 6551.959 | 15 | 26532 | 15.2 |
| 6777.707 | 15 | 31368 | 15.0 |
| 6873.717 | 100 | 300652 | 101.7 |
| 7176.764 | 16 | 22578 | 17.2 |
| 13844.029 | 3 | 1969 | 6.0 |

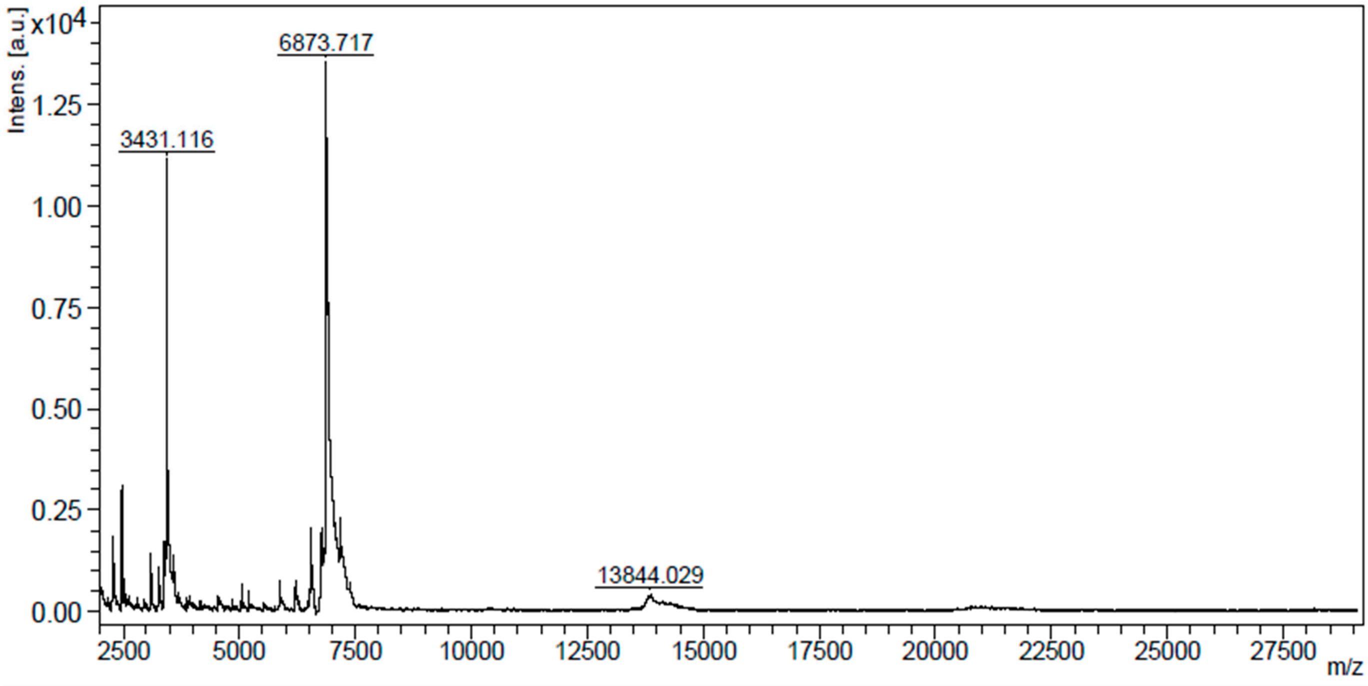


I-7 05121020240_H31 2024-12-05T16:10:42.000

**Figure S56.** MALDI-TOF MS spectrum of siPCSK9_6_6-aS

| Name | Sequence (5′-3′) |
|---|---|
| siPCSK9_6_6-aS | mUmAmAmAmUfGfUfCmUmGmCfUfUfGmCmUmUmGmGmGmU |
| Calculated Mw (H+ form) | 6890,38 |
| Founded Mw | 6873,72 |

siPCSK9-6-7

| m/z | Rel. Intens. | Area | SN |
|---|---|---|---|
| 3445.643 | 7 | 50743 | 41.7 |
| 3597.027 | 4 | 23554 | 20.6 |
| 3616.557 | 2 | 27045 | 6.0 |
| 6584.732 | 13 | 36018 | 20.3 |
| 6904.978 | 100 | 403033 | 153.4 |
| 7201.424 | 1 | 18681 | 21.5 |

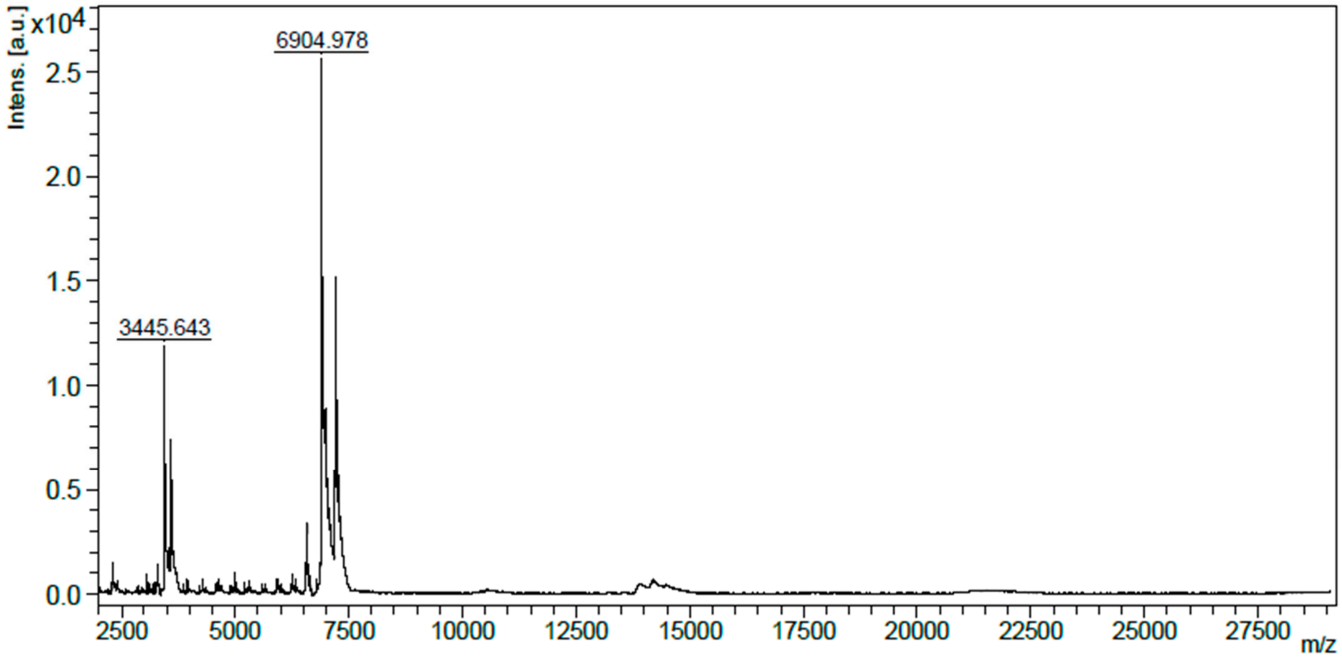


*9 12122024\0_D6\1* *2024-12-12T15:19:51.000*

**Figure S57.** MALDI-TOF MS spectrum of siPCSK9_6_7-S

| Name | Sequence (5′-3′) |
|---|---|
| siPCSK9_6_7-S | mCmCmAmAmGmCmAmAmGmCfAfGfAmCmAmUmUmUmAmUmC |
| Calculated Mw (H+ form) | 6921,61 |
| Founded Mw | 6904,98 |

siPCSK9-6-7

| m/z | Rel. Intens. | Area | SN |
|---|---|---|---|
| 3434.241 | 83 | 48911 | 31.6 |
| 6551.873 | 14 | 6206 | 9.1 |
| 6779.197 | 15 | 16095 | 10.0 |
| 6874.855 | 100 | 142741 | 65.0 |
| 7175.730 | 19 | 6879 | 12.9 |
| 13838.612 | 4 | 1351 | 3.9 |

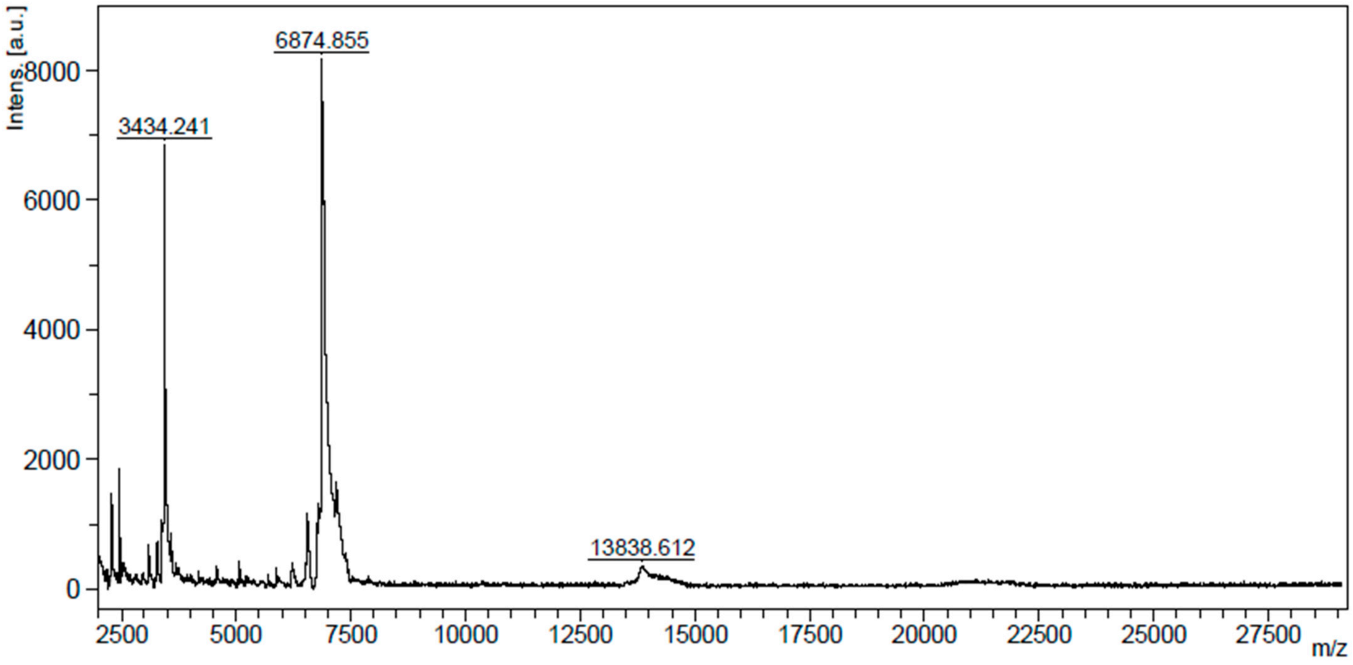


I-8 05121020240_H41 2024-12-05T16:11:05.000

**Figure S58.** MALDI-TOF MS spectrum of siPCSK9_6_7-aS

| Name | Sequence (5′-3′) |
|---|---|
| siPCSK9_6_7-aS | mUmAmAmAfUfGfUmCmUmGmCmUfUfGfCmUmUmGmGmGmU |
| Calculated Mw (H+ form) | 6890,38 |
| Founded Mw | 6874,86 |

siPCSK9-6-8

| m/z | Rel. Intens. | Area | SN |
|---|---|---|---|
| 3454.472 | 36 | 30822 | 22.5 |
| 3605.700 | 27 | 21276 | 17.0 |
| 6591.478 | 15 | 23694 | 11.7 |
| 6932.946 | 100 | 174501 | 76.5 |
| 7216.086 | 68 | 115369 | 52.6 |
| 14219.743 | 7 | 3214 | 9.4 |

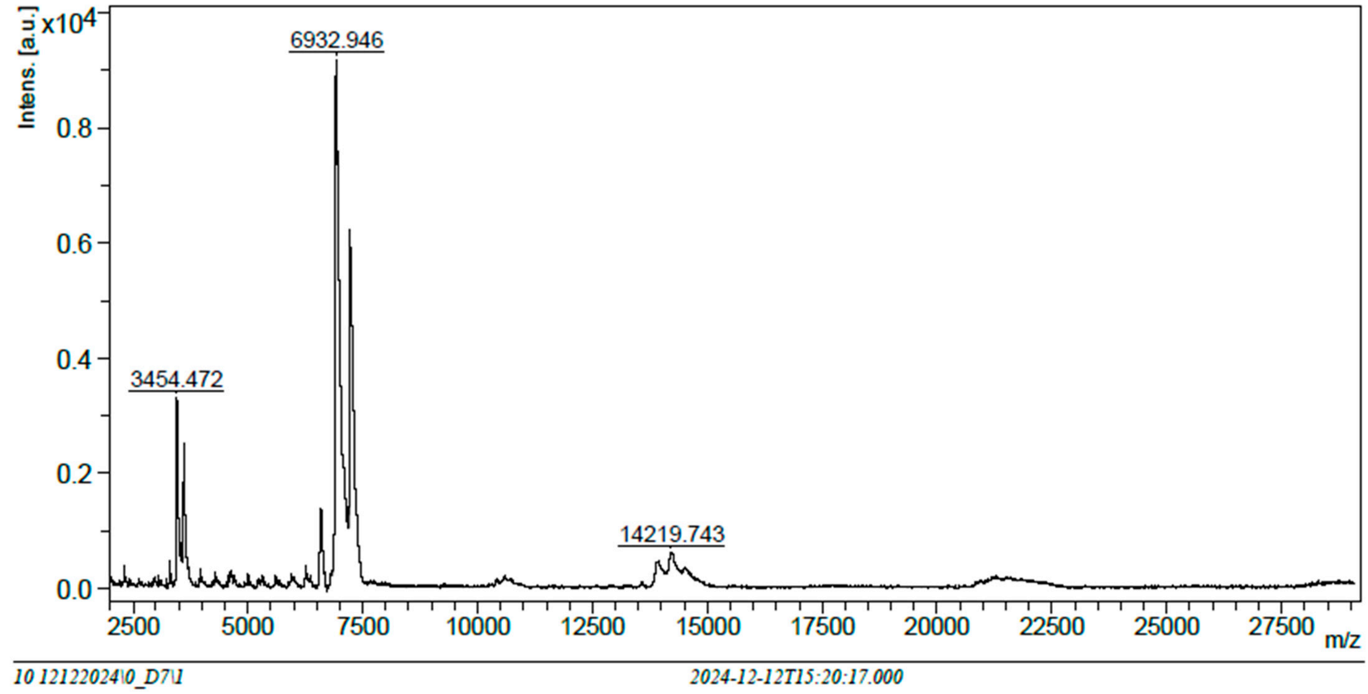


**Figure S59.** MALDI-TOF MS spectrum of siPCSK9_6_8-S

| Name | Sequence (5′-3′) |
|---|---|
| siPCSK9_6_8-S | mCmCmAmAmGmCmAmAmGmCfAfGfAmCmAmUmUmUmAmUmC |
| Calculated Mw (H+ form) | 6921,61 |
| Founded Mw | 6932,95 |

siPCSK9-6-8

| m/z | Rel. Intens. | Area | SN |
|---|---|---|---|
| 2465.583 | 6 | 20845 | 7.8 |
| 3431.421 | 7 | 51778 | 14.9 |
| 6552.944 | 17 | 36560 | 20.5 |
| 6874.622 | 100 | 329240 | 125.1 |
| 7179.000 | 13 | 49443 | 17.1 |
| 13823.485 | 3 | 2672 | 5.8 |

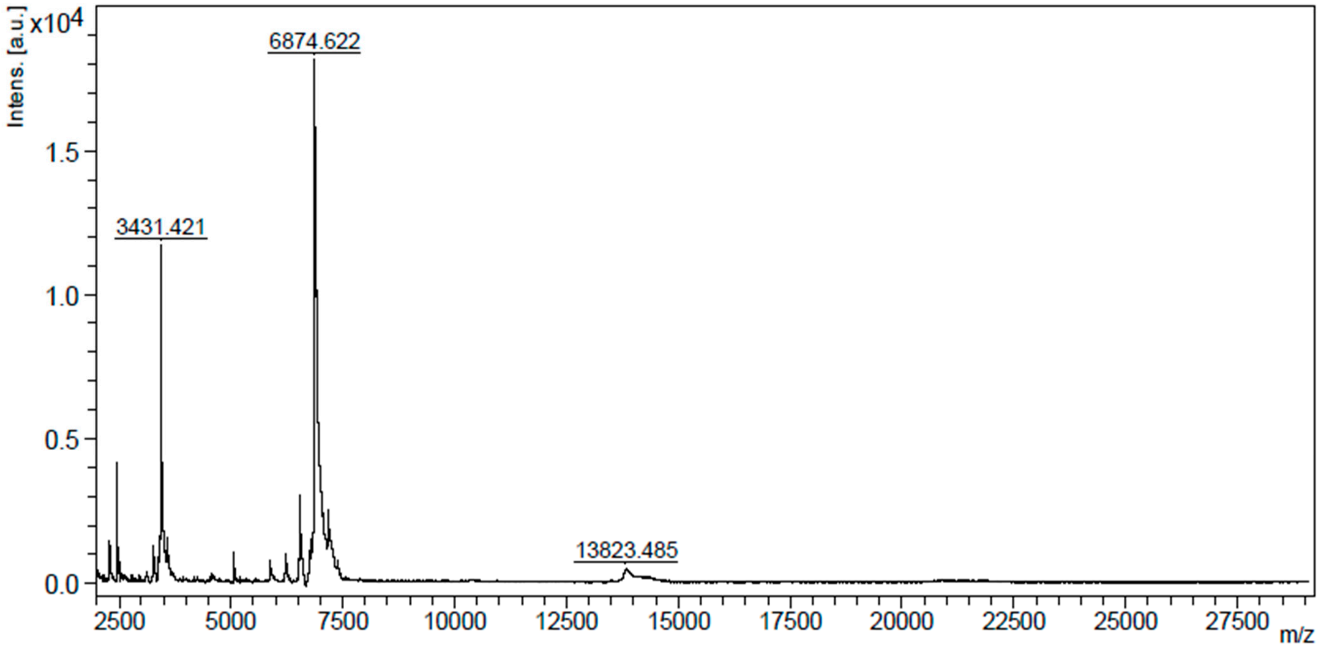


I-9 0512102024\0_H5\1 2024-12-05T16:11:28.000

**Figure S60.** MALDI-TOF MS spectrum of siPCSK9_6_8-aS

| Name | Sequence (5′-3′) |
|---|---|
| siPCSK9_6_8-aS | mUfAmAmAmUfGmUfCmUmGmCfUmUfGmCmUmUmGmGfGmU |
| Calculated Mw (H+ form) | 6890,38 |
| Founded Mw | 6874,62 |

siPCSK9-6-9

| m/z | Rel. Intens. | Area | SN |
|---|---|---|---|
| 2298.435 | 6 | 13348 | 6.3 |
| 3057.118 | 6 | 13407 | 6.5 |
| 3449.542 | 36 | 72624 | 38.2 |
| 3597.338 | 2 | 32521 | 9.2 |
| 6585.528 | 12 | 41720 | 17.8 |
| 6906.058 | 100 | 434406 | 146.7 |
| 7209.950 | 62 | 268546 | 91.9 |
| 14184.655 | 3 | 4968 | 10.0 |

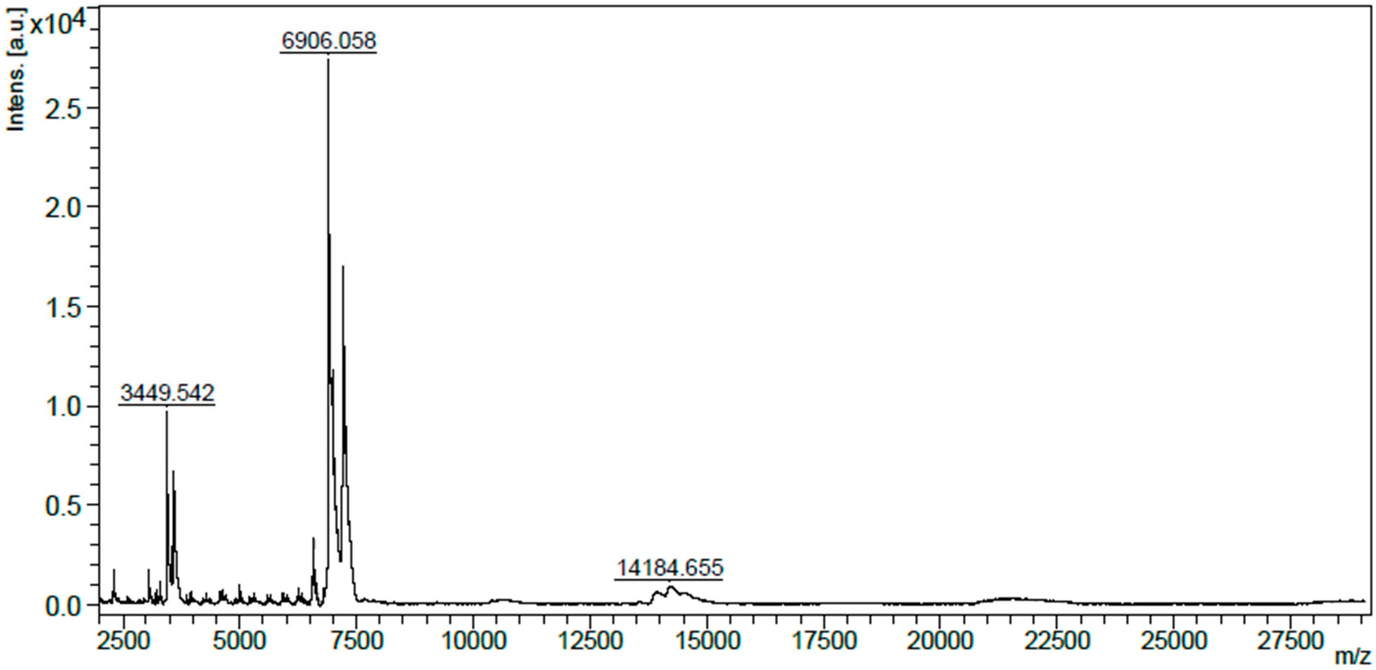


11 12122024\0_D8\1 2024-12-12T15:20:36.000

**Figure S61.** MALDI-TOF MS spectrum of siPCSK9_6_9-S

| Name | Sequence (5′-3′) |
|---|---|
| siPCSK9_6_9-S | mCmCmAmAmGmCmAmAmGmCfAfGfAmCmAmUmUmUmAmUmC |
| Calculated Mw (H+ form) | 6921,61 |
| Founded Mw | 6906,06 |

siPCSK9-6-9

| m/z | Rel. Intens. | Area | SN |
|---|---|---|---|
| 3148.926 | 8 | 12568 | 7.1 |
| 3626.304 | 18 | 10937 | 5.9 |
| 3786.968 | 77 | 40827 | 25.3 |
| 5746.047 | 8 | 2339 | 3.7 |
| 6906.637 | 9 | 3979 | 5.4 |
| 7258.142 | 20 | 11425 | 12.8 |
| 7602.139 | 100 | 67992 | 63.7 |
| 7884.816 | 14 | 3023 | 9.1 |
| 15255.242 | 5 | 1487 | 6.9 |

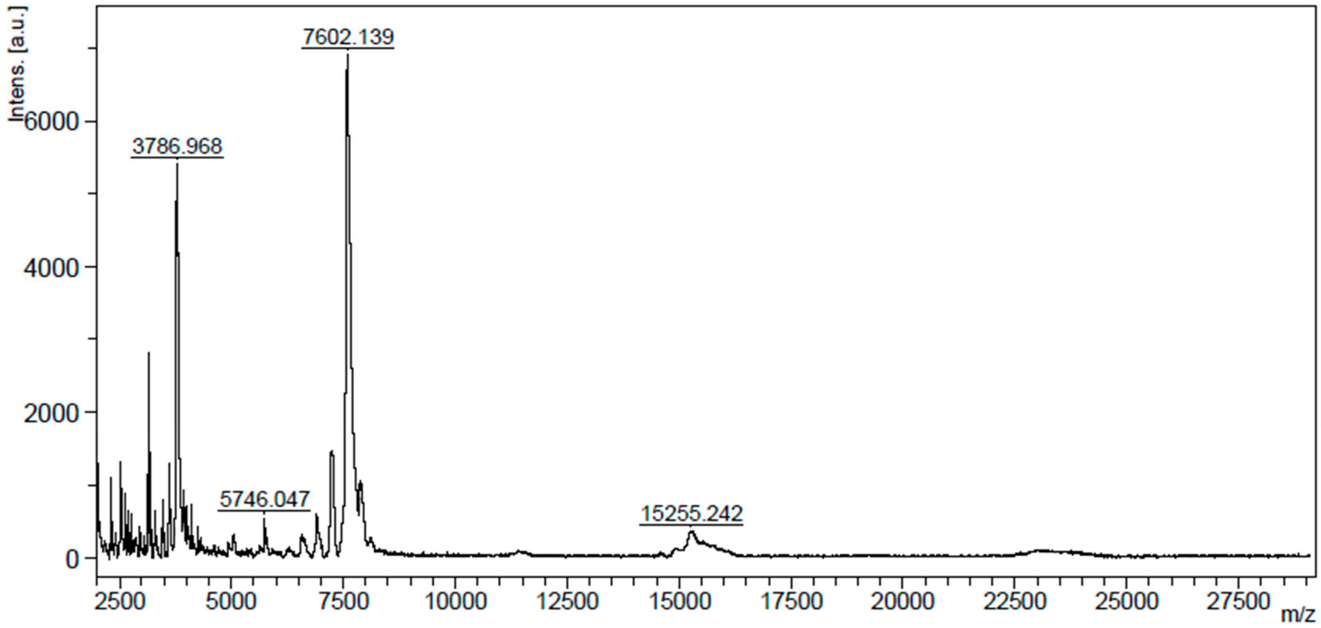


I-2 05121020240_G10\1 2024-12-05T16:08:48.000

**Figure S62.** MALDI-TOF MS spectrum of siPCSK9_6_9-aS

| Name | Sequence (5′-3′) |
|---|---|
| siPCSK9_6_9-aS | mUfAmAmAmUfGmUfCmUmGmCfUmUfGmCmUmUmGmGfGmUmAmA |
| Calculated Mw (H+ form) | 7576,86 |
| Founded Mw | 7602,14 |

siPCSK9-6-10

| m/z | Rel. Intens. | Area | SN |
|---|---|---|---|
| 2298.172 | 15 | 17690 | 10.2 |
| 3056.397 | 8 | 6340 | 5.8 |
| 3289.972 | 8 | 7666 | 6.1 |
| 3445.421 | 8 | 25344 | 33.8 |
| 3596.326 | 4 | 11680 | 17.6 |
| 3615.617 | 2 | 8531 | 8.5 |
| 6585.801 | 15 | 20488 | 19.4 |
| 6906.100 | 100 | 150741 | 126.8 |
| 7203.202 | 1 | 6258 | 24.6 |

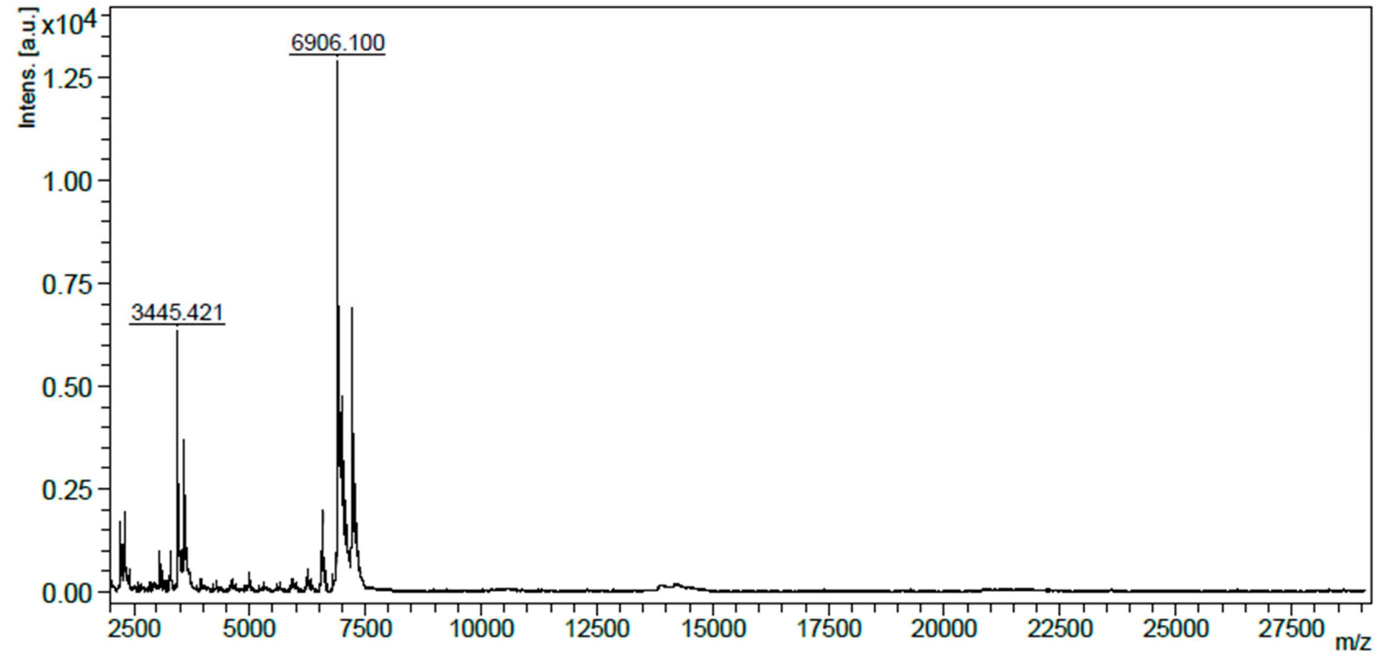


5 12122024\0_D2\1 2024-12-12T15:18:11.000

**Figure S63.** MALDI-TOF MS spectrum of siPCSK9_6_10-S

| Name | Sequence (5′-3′) |
|---|---|
| siPCSK9_6_10-S | mCmCmAmAmGmCmAmAmGmCfAfGfAmCmAmUmUmUmAmUmC |
| Calculated Mw (H+ form) | 6921,61 |
| Founded Mw | 6906,10 |

siPCSK9-6-10

| m/z | Rel. Intens. | Area | SN |
|---|---|---|---|
| 3486.126 | 46 | 20243 | 18.3 |
| 6613.509 | 15 | 9909 | 8.1 |
| 6976.156 | 100 | 97577 | 53.5 |
| 14007.855 | 6 | 933 | 5.3 |

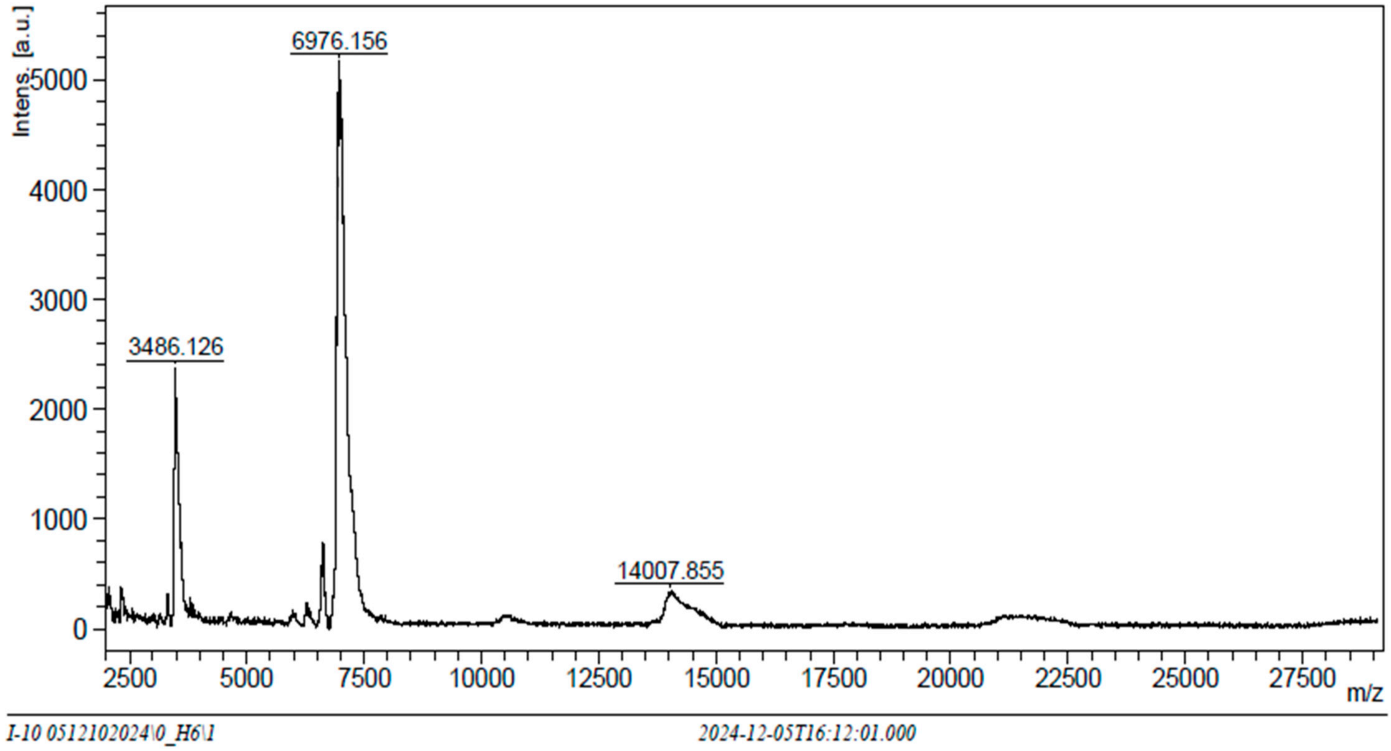


**Figure S64.** MALDI-TOF MS spectrum of siPCSK9_6_10-aS

| Name | Sequence (5′-3′) |
|---|---|
| siPCSK9_6_10-aS | mUmAmAmAmUmGmUmCfUfGfCmUmUmGmCmUmUmGmGmGmU |
| Calculated Mw (H+ form) | 6926,5 |
| Founded Mw | 6976,16 |

siPCSK9-6-11

| m/z | Rel. Intens. | Area | SN |
|---|---|---|---|
| 2298.820 | 8 | 2418 | 8.1 |
| 3446.305 | 8 | 12085 | 44.0 |
| 3597.486 | 4 | 5345 | 20.1 |
| 6588.222 | 10 | 5112 | 15.2 |
| 6908.517 | 100 | 68047 | 160.8 |
| 7205.824 | 1 | 2930 | 45.2 |

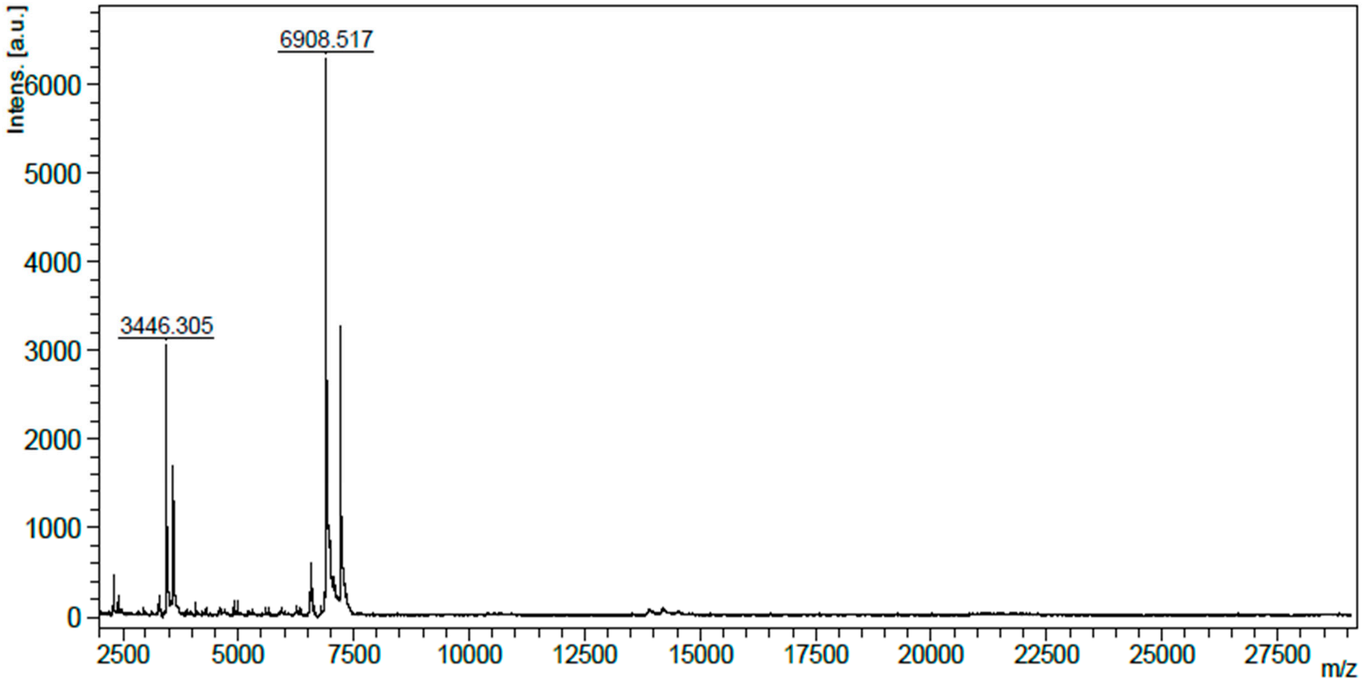


**Figure S65.** MALDI-TOF MS spectrum of siPCSK9_6_11-S

| Name | Sequence (5′-3′) |
|---|---|
| siPCSK9_6_11-aS | mCmCmAmAmGmCfAmAfGfCmAmGmAmCmAmUmUmUmAmUmC |
| Calculated Mw (H+ form) | 6921,61 |
| Founded Mw | 6908,52 |

siPCSK9-6-11

| m/z | Rel. Intens. | Area | SN |
|---|---|---|---|
| 2453.908 | 11 | 20032 | 11.2 |
| 3415.958 | 56 | 39926 | 29.3 |
| 3755.084 | 3 | 6445 | 7.3 |
| 5049.452 | 11 | 8373 | 6.8 |
| 6515.585 | 16 | 19271 | 12.7 |
| 6837.262 | 100 | 186383 | 81.7 |
| 13767.341 | 4 | 1817 | 8.2 |

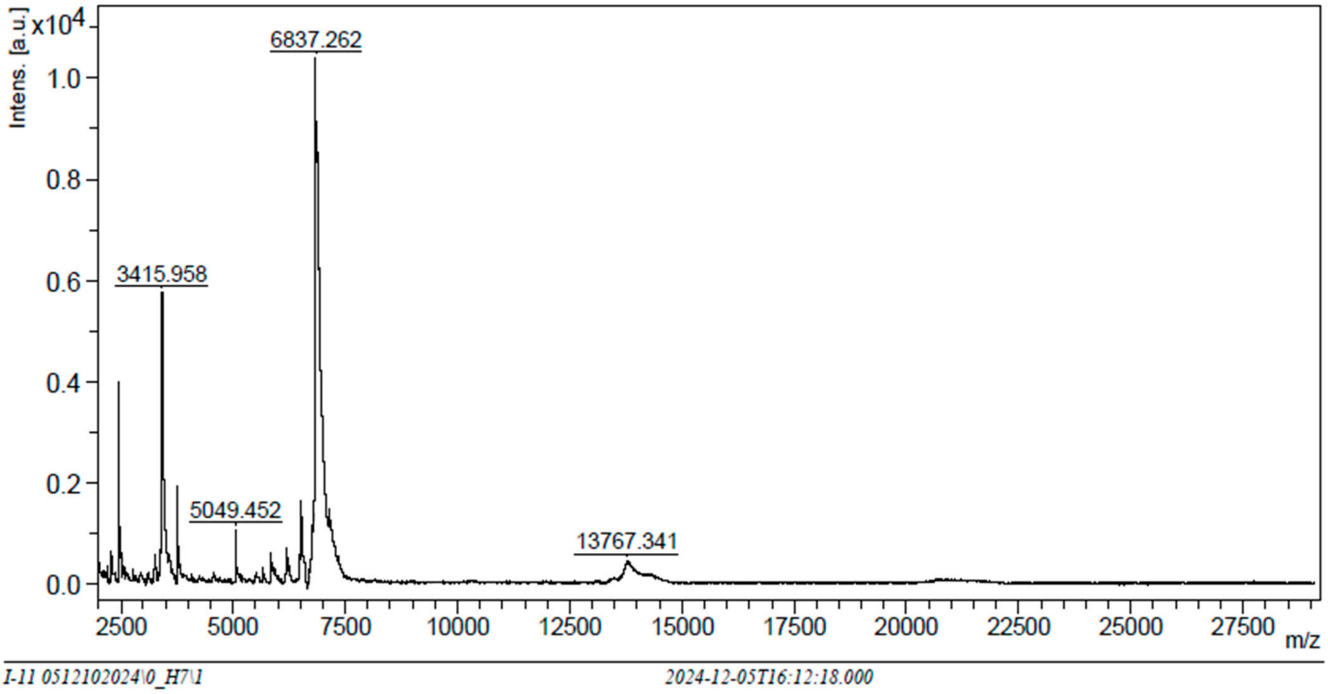


**Figure S66.** MALDI-TOF MS spectrum of siPCSK9_6_11-aS

| Name | Sequence (5′-3′) |
|---|---|
| siPCSK9_6_11-S | mUfAmAfAfUfGmUfCmUfGmCmUmUfGmCfUmUfGmGmGmU |
| Calculated Mw (H+ form) | 6854,26 |
| Founded Mw | 6837,26 |

siPCSK9_6_12

| m/z | Rel. Intens. | Area | SN |
|---|---|---|---|
| 2295.058 | 13 | 5540 | 7.7 |
| 3439.811 | 7 | 21406 | 17.0 |
| 3594.042 | 28 | 15083 | 18.2 |
| 3621.297 | 18 | 12286 | 11.8 |
| 6895.691 | 100 | 77037 | 127.2 |
| 6933.842 | 27 | 23481 | 34.8 |
| 7191.962 | 1 | 3292 | 32.9 |
| 7237.093 | 16 | 14950 | 20.7 |

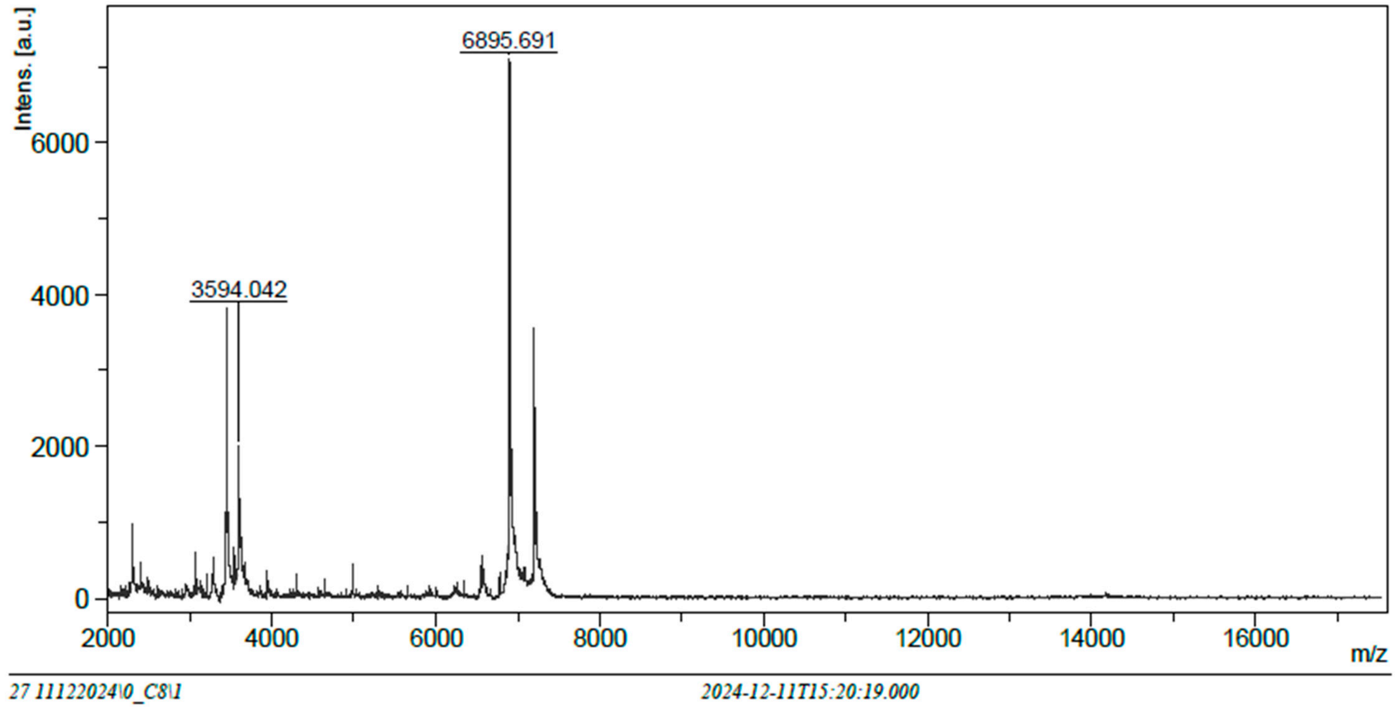


**Figure S67.** MALDI-TOF MS spectrum of siPCSK9_6_12-S

| Name | Sequence (5′-3′) |
|---|---|
| siPCSK9_6_12-S | fCmCmAmAmGmCmAmAmGmCfAfGfAmCmAmUmUmUmAmUmC |
| Calculated Mw (H+ form) | 6909,57 |
| Founded Mw | 6895,69 |

siPCSK9_6_12

| m/z | Rel. Intens. | Area | SN |
|---|---|---|---|
| 2441.907 | 23 | 41945 | 22.1 |
| 3419.485 | 10 | 47627 | 13.1 |
| 5049.101 | 15 | 14435 | 9.8 |
| 5853.465 | 7 | 6920 | 5.3 |
| 6197.457 | 7 | 7831 | 6.1 |
| 6528.071 | 16 | 26095 | 13.8 |
| 6850.851 | 100 | 272749 | 87.4 |
| 7153.216 | 25 | 25798 | 22.7 |
| 13760.866 | 3 | 2091 | 5.8 |

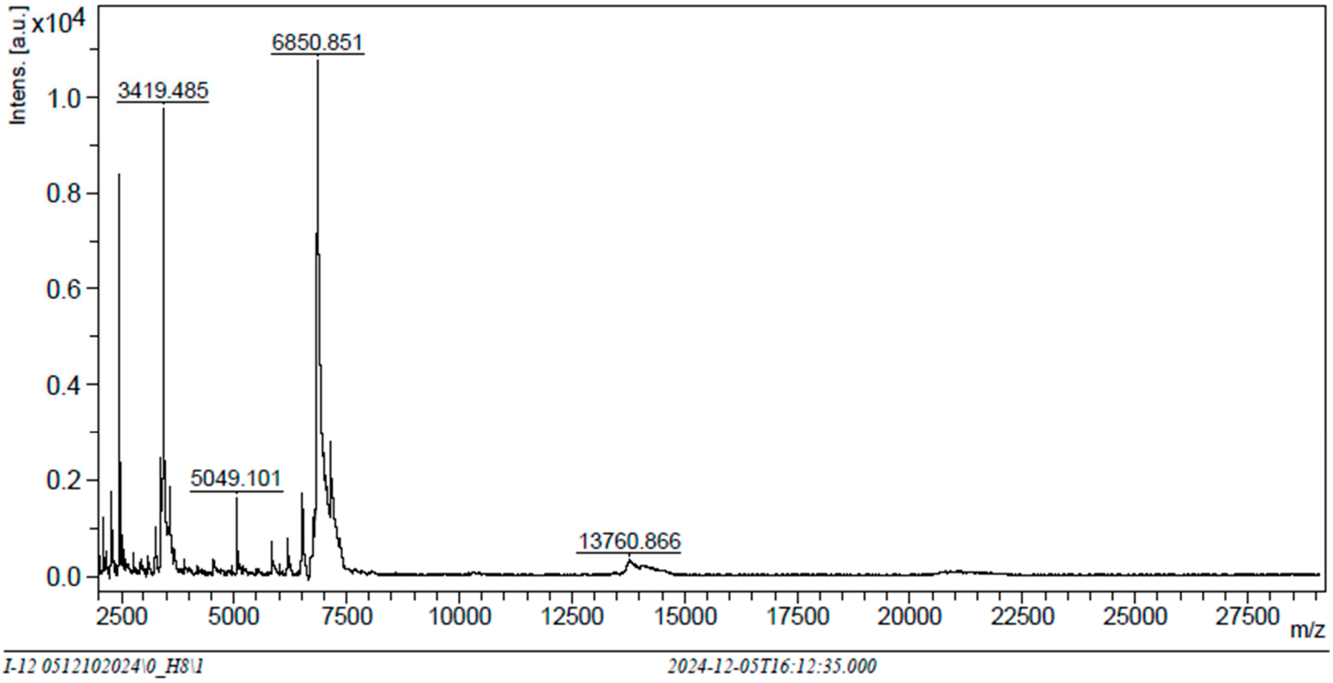


**Figure S68.** MALDI-TOF MS spectrum of siPCSK9_6_12-aS

| Name | Sequence (5′-3′) |
|---|---|
| siPCSK9_6_12-aS | mUfAmAfAmUfGmUmCmUmGmCfUmUfGmCfUmUfGmGfGmU |
| Calculated Mw (H+ form) | 6866,3 |
| Founded Mw | 6850,85 |

siPCSK9_1_C

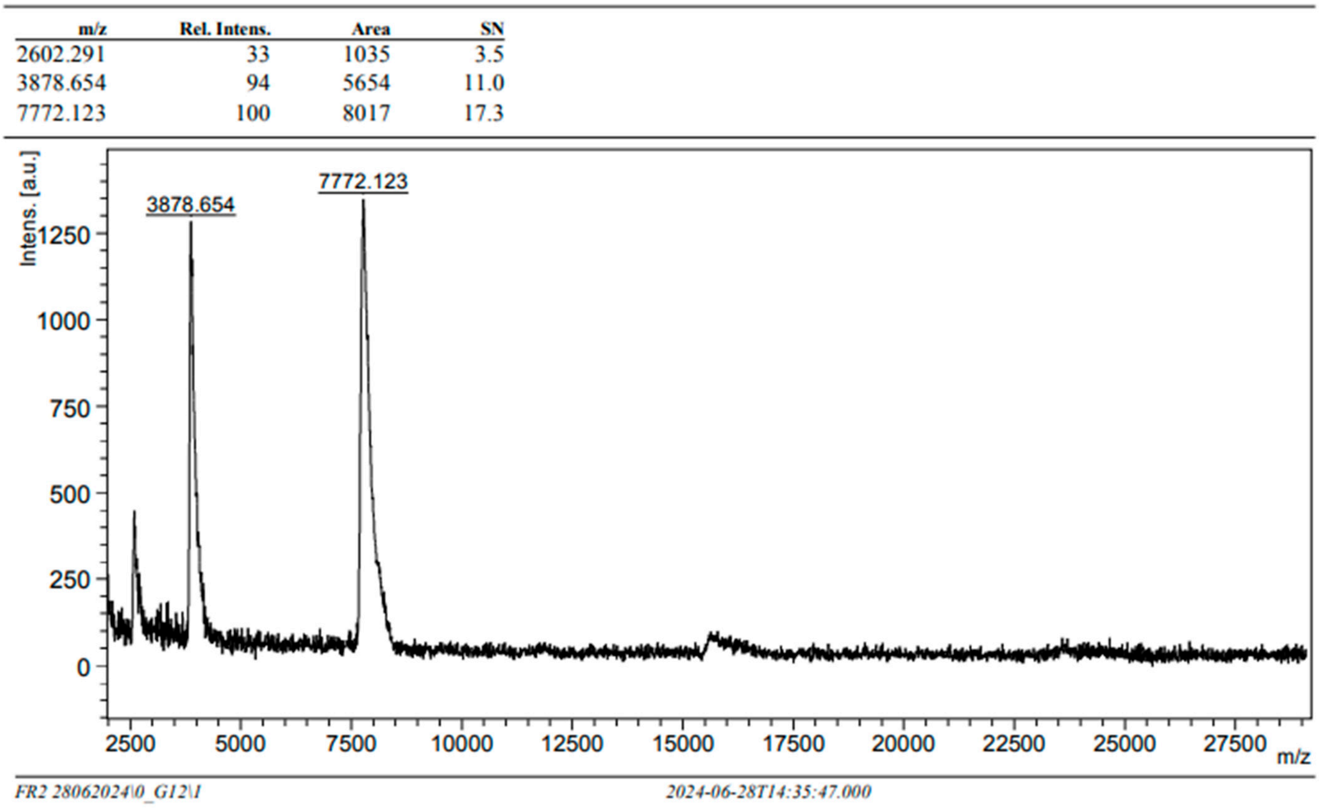


**Figure S69.** MALDI-TOF MS spectrum of siPCSK9_1-C-aS

| Name | Sequence (5′-3′) |
|---|---|
| siPCSK9_1-C-aS | mA(s)fC(s)mAfAfAfAmGfCmAfAmAfAmCfAmGfGmUfCmUmAmG(s)mA(s)mA |
| Calculated Mw (H+ form) | 7710.25 |
| Founded Mw | 7772.123 |

siPCSK9_1_C

| m/z | Rel. Intens. | Area | SN |
|---|---|---|---|
| 2166.980 | 34 | 963 | 3.3 |
| 2873.454 | 68 | 5624 | 7.3 |
| 4310.681 | 100 | 14637 | 12.2 |
| 8631.495 | 45 | 9616 | 9.5 |

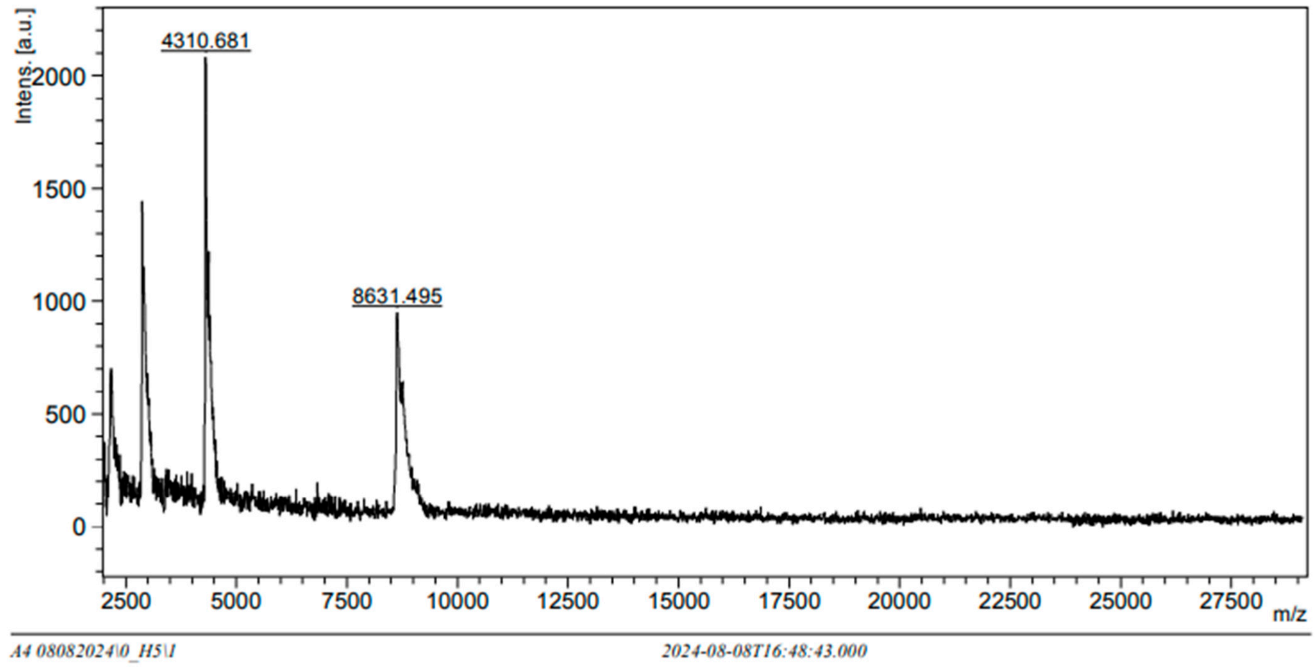


**Figure S70.** MALDI-TOF MS spectrum of siPCSK9_1-C-S

| Name | Sequence (5′-3′) |
|---|---|
| siPCSK9_1-C-S | mC(s)mU(s)mAmGmAmCfCmUfGmUdTmUmUmGmCmUmUmUmUmGmU-L96 |
| Calculated Mw (H+ form) | 8657.57 |
| Founded Mw | 8631.495 |

siPCSK9_2_12-C

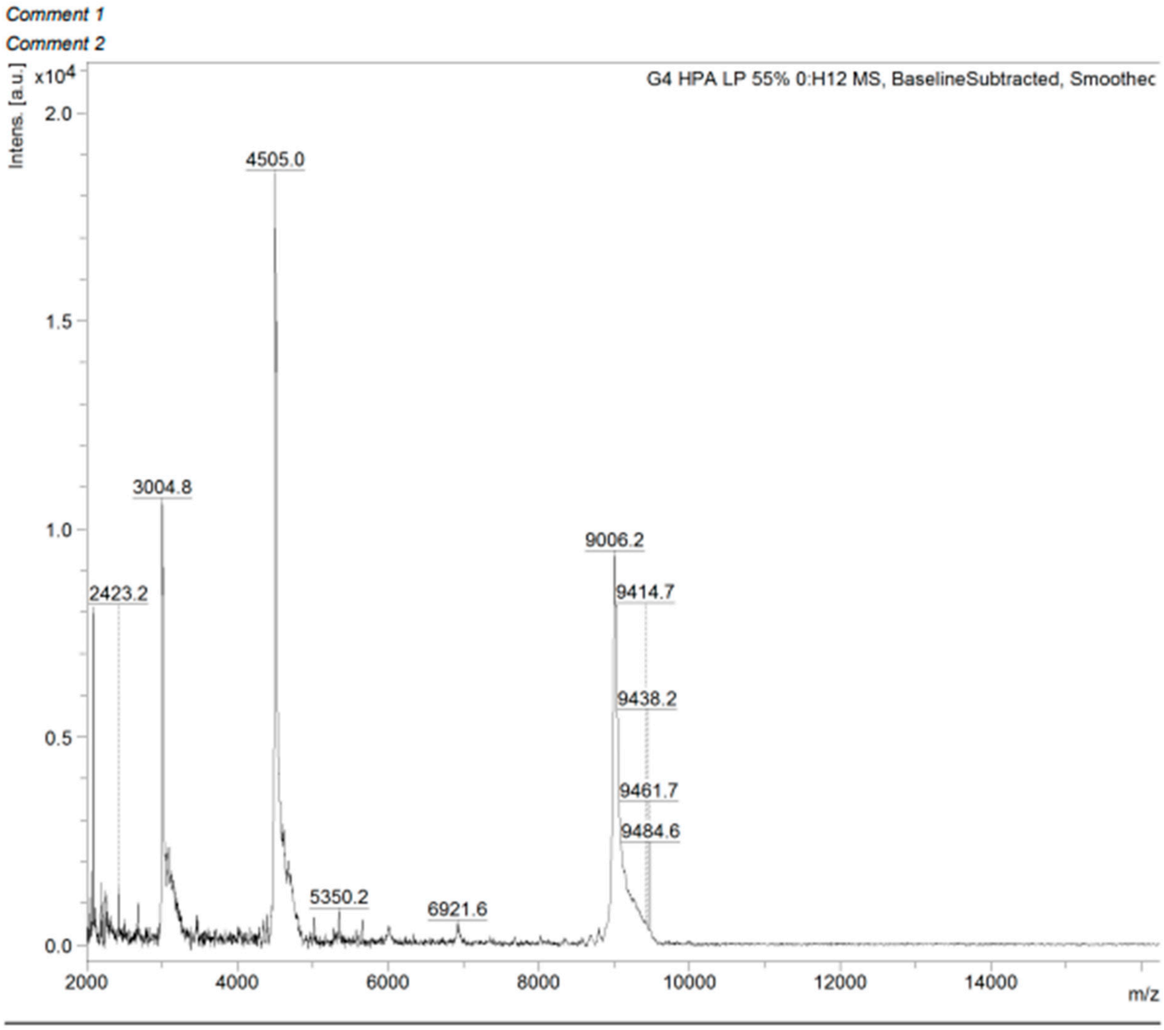


| m/z | S/N | Quality Fac. | Res. | Intens. | Area |
|---|---|---|---|---|---|
| 9006.2 | 123 | | 396 | 9425 | 379676 |
| 9023.0 | 107 | | 349 | 8172 | 401690 |
| 4505.0 | 77 | | 471 | 18571 | 319478 |
| 4515.5 | 62 | | 517 | 14998 | 288916 |
| 3004.8 | 35 | | 444 | 10695 | 119037 |
| 3011.8 | 30 | | 469 | 9083 | 143352 |
| 2087.4 | 25 | | 380 | 8135 | 55407 |
| 9140.4 | 23 | | 508 | 1789 | 72865 |
| 9191.2 | 18 | | 1200 | 1342 | 26780 |
| 4551.4 | 17 | | 1133 | 4133 | 50686 |
| 9210.3 | 16 | | 2231 | 1228 | 11248 |
| 9218.0 | 16 | | 1932 | 1217 | 11943 |
| 9229.8 | 15 | | 1847 | 1132 | 10621 |
| 9240.7 | 15 | | 1312 | 1107 | 19231 |

**Figure S71.** MALDI-TOF MS spectrum of siPCSK9_2_12-C-S

| Name | Sequence (5′-3′) |
|---|---|
| siPCSK9_2_12-C-S | L96-fC(s)mAmAmGmCmAmAmGmCmAfGfAfCmAmUmUmUmAmUmCmU |
| Calculated Mw (H+ form) | 8761.82 |
| Calculated Mw (Na salt) | 9221.82 |
| Founded Mw | 9006.2 |

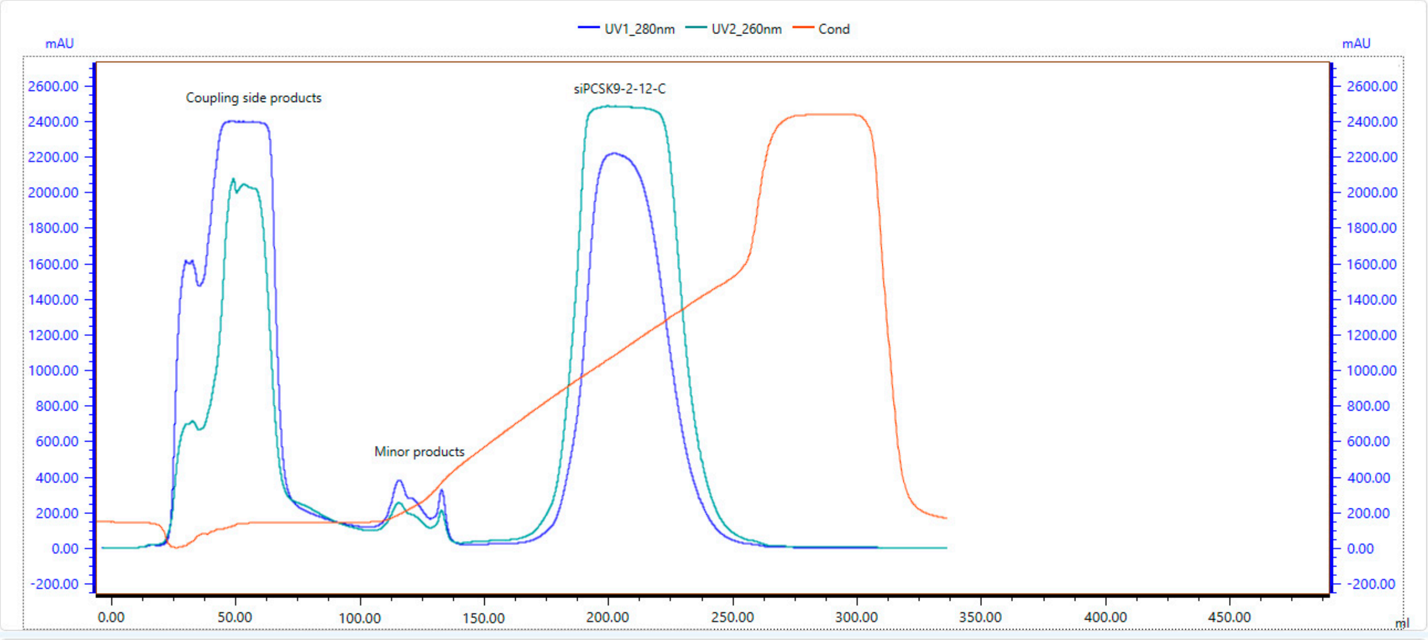


**Figure S72.** Typical preparative-scale AE-IEX chromatogram of siPCSK9-2-12-C-S after conjugation of DMT-on purified benzylic ester by RP HPLC and GalNAc-amine.

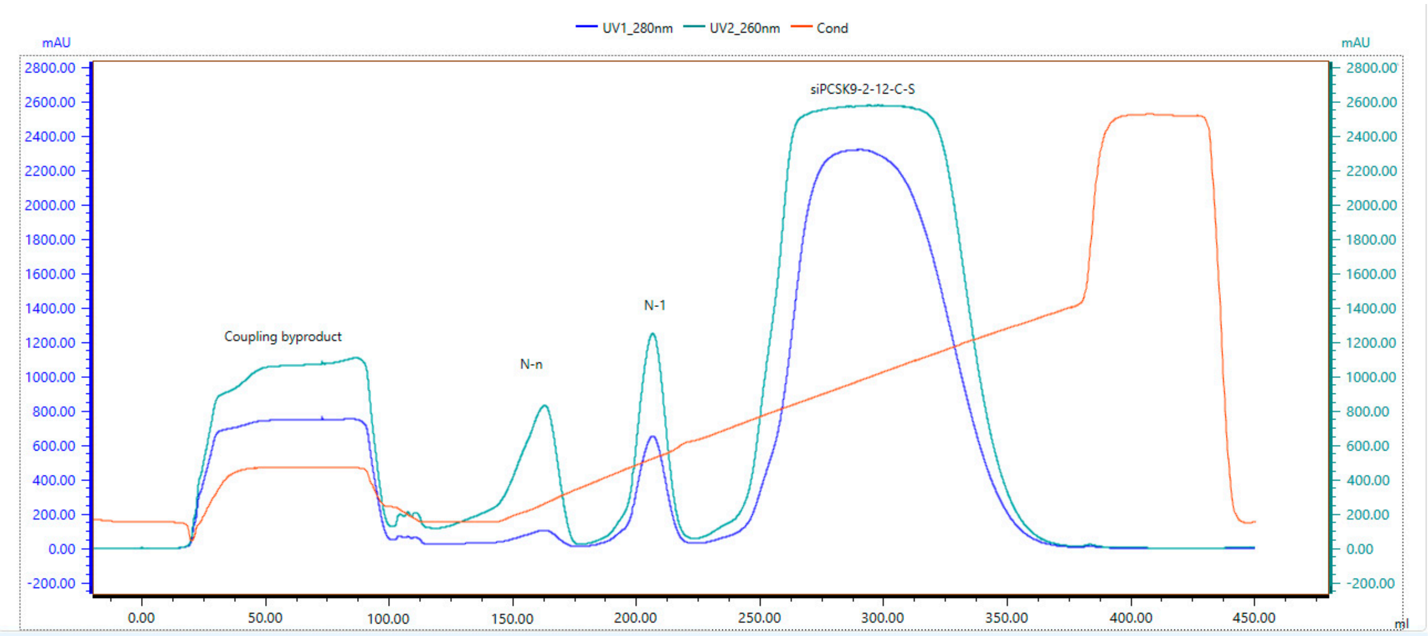


**Figure S73.** Typical preparative-scale AE-IEX chromatogram of siPCSK9-2-12-C-S after conjugation of DMT-off purified benzylic ester by AE-IEX and GalNAc-amine.
